# Induced magnetosphere of Mars responds fast to interplanetary magnetic field rotation

**DOI:** 10.1038/s41467-026-76861-1

**Published:** 2026-08-19

**Authors:** Rentong Lin, Jingyi Zhou, Shiyong Huang, Zhigang Yuan, Yuming Wang, Honghong Wu, Kui Jiang, Sibo Xu, Zhuxuan Zou, Zhenpeng Su, Tielong Zhang, Yue Dong, Qiyang Xiong, Huxing Huang

**Affiliations:** 1 School of Earth and Space Science and Technology, Wuhan University, Wuhan, People’s Republic of China; 2 National Key Laboratory of Deep Space Exploration/School of Earth and Space Sciences, University of Science and Technology of China, Hefei, China; 3 CAS Center for Excellence in Comparative Planetology/CAS Key Laboratory of Geospace Environment/Mengcheng National Geophysical Observatory, University of Science and Technology of China, Hefei, China; 4 Institute of Space Science and Applied Technology, Harbin Institute of Technology, Shenzhen, People’s Republic of China; 5 Space Research Institute, Austrian Academy of Sciences, Graz, Austria

**Keywords:** Inner planets, Magnetospheric physics

## Abstract

Interactions between the ionospheres of unmagnetized bodies and ambient plasma flows form induced magnetospheres. The induced magnetosphere is generally affected by external magnetic fields. Observations of response processes of the induced magnetosphere to external magnetic fields are important for understanding global dynamical processes in unmagnetized planets. Here, using simultaneous observations of Tianwen-1 and Mars Atmosphere and Volatile EvolutioN missions, we show the response of Mars’ induced magnetosphere to interplanetary magnetic field (IMF) rotation. The magnetic field in Mars’ induced magnetosphere rotated within at least 165 seconds following the sudden IMF clock-angle change and gradually stabilized. The convective electric field rotated accordingly, bringing the pickup oxygen-ion plume into the spacecraft’s field of view within 3 minutes. These short timescales demonstrate that Mars’ induced magnetosphere is highly dynamic and sensitive to external magnetic-field variations. IMF changes should therefore be regarded as a common form of Mars’ space weather, highlighting the importance of upstream IMF monitoring and short-term forecasting.

## Abstract (web summary)

Interaction between solar wind and Mars’ ionosphere forms an induced magnetosphere. Here, the authors show that Mars’ induced magnetosphere responds rapidly in minutes as the interplanetary magnetic field rotates.

## Introduction

Since unmagnetized planets or moons, such as Mars, Venus, and Titan, lack global intrinsic magnetic fields^1–3^, the solar wind and its convected interplanetary magnetic field (IMF) interact with the upper atmosphere/ionosphere, which induces currents and the formation of the induced magnetosphere^4^. In a steady state, the induced currents flow at boundary layers. When there are temporal variations in the direction or magnitude of the external magnetic field, electrical currents are induced on the surface, inside the body, or in the ionosphere. The magnetic fields associated with the induced currents generally exclude the external magnetic field from the ionosphere. Additionally, the external magnetic field is generally frozen into the plasmas, and external plasmas can be obstructed by the ionosphere, which results in the draping of the magnetic field around the ionosphere. Thus, the induced magnetosphere of unmagnetized planets or moons is generally affected by an external magnetic field, usually referred to as the IMF^5,6^. The variability of the IMF affects the evolution of planetary pickup ions^7^, the shape of the induced magnetosphere^8–10^, the formation of Mars’ induced magnetosphere boundary^11,12^, the magnetic reconnection at Mars’ induced magnetosphere boundary, which channels the planetary ions to escape from Mars^13^, and the mini magnetosphere over the crustal field^1,14–16^, which directly influences planetary ion escape^17,18^. These findings suggest that the direction of the IMF is significantly involved in the formation and dynamics of the induced magnetosphere and the upper atmosphere/ionosphere.

The induced magnetospheres of the unmagnetized planet/moon (such as Mars, Venus, and Titan) with the atmosphere/ionosphere share similar formation mechanisms, but they have different features because of their different physical parameters and external conditions. Therefore, the response process of the induced magnetosphere to external conditions has been investigated for decades. The evolution of the magnetic field can generally be described by the magnetic induction equation, and the evolution time of the magnetic field depends on the speed of the plasmas and the spatial scale of the region^19^. On the basis of an MHD model of the plasma interaction of Titan, Neubauer et al.^20^. reported deceleration of the plasma bulk speed from ~100 km/s in the incident magnetospheric flow to ~0.1 km/s in the ionosphere of Titan, which corresponds to a drastically increased residence time of the magnetic field lines in the ionosphere. The time scale increases to several hours, indicating that a magnetic field line may remain trapped in the ionosphere of Titan for several hours^21,22^ or even longer, ^23^ after the direction of the external magnetic field changes. These trapped field lines are referred to as fossilized magnetic fields^20^. However, other simulations on the response of Mars’ induced magnetospheres to IMF rotation have shown that the magnetic field and oxygen ion plume rapidly rotate with the IMF and that the recovery timescale of the response process is as short as minutes^24–26^. Simulations on Venus show that the dayside Venusian-induced magnetosphere also responds quickly, in minutes, to IMF rotation^27^. In contrast to the numerous simulation studies, observations of the response process of the induced magnetosphere to the external magnetic field are lacking, despite numerous planetary exploration missions that have been conducted on Mars, Venus, Titan, etc. On the basis of observations by Cassini in the T32 flyby, it was proposed that the magnetic field of the induced magnetosphere of Titan is a fossil field that can memorize the external magnetic field for hours^28^. This study, however, is based on single-orbiter in situ measurements, which are unable to both identify internal changes and detect external changes. Therefore, more in situ observations of the response process of the induced magnetosphere to changes in the external magnetic field are needed.

Although there have been periods of multiple satellites orbiting Mars simultaneously, no two magnetometer-equipped orbiters have operated simultaneously, which implies that it was unable to observe the IMF and magnetic field of the induced magnetosphere simultaneously. Owing to the lack of joint satellite observations that allow simultaneous monitoring of the solar wind and IMF as well as the induced magnetosphere, it was difficult to quantify the response process, including the onset, delay, and possible recovery time of the induced magnetosphere to IMF variability. The arrival of the Tianwen-1 orbiter at Mars provides the opportunity to perform such quantitative studies.

With China’s first Mars exploration mission, Tianwen-1^29,30^, which entered Mars orbit in 2021, as well as the U.S. Mars Atmosphere and Volatile EvolutioN (MAVEN) satellite^31^, which arrived at Mars in 2014 and has been in service since then, it is now available for us to perform simultaneous observations of upstream solar wind and the downstream structure, e.g., the Martian magnetosheath, induced magnetosphere, and pile-up region^32–34^.

In this work, we report two events of the rapid response process of Mars’ induced magnetosphere in response to the rotation of the IMF, using the joint observations from the Tianwen-1 and MAVEN satellites. The first event shows a delayed response and provides a lower limit of 165 seconds for adjustment of magnetic field, although the exact recovery endpoint cannot be determined because of subsequent low-altitude crustal-field interference. The second event shows that the convective electric field deflected in response to the IMF rotation, followed by the pick-up of the oxygen ion plume in less than 3 minutes.

## Results

### Response of the magnetic field to IMF rotation

**Fig. 1:**
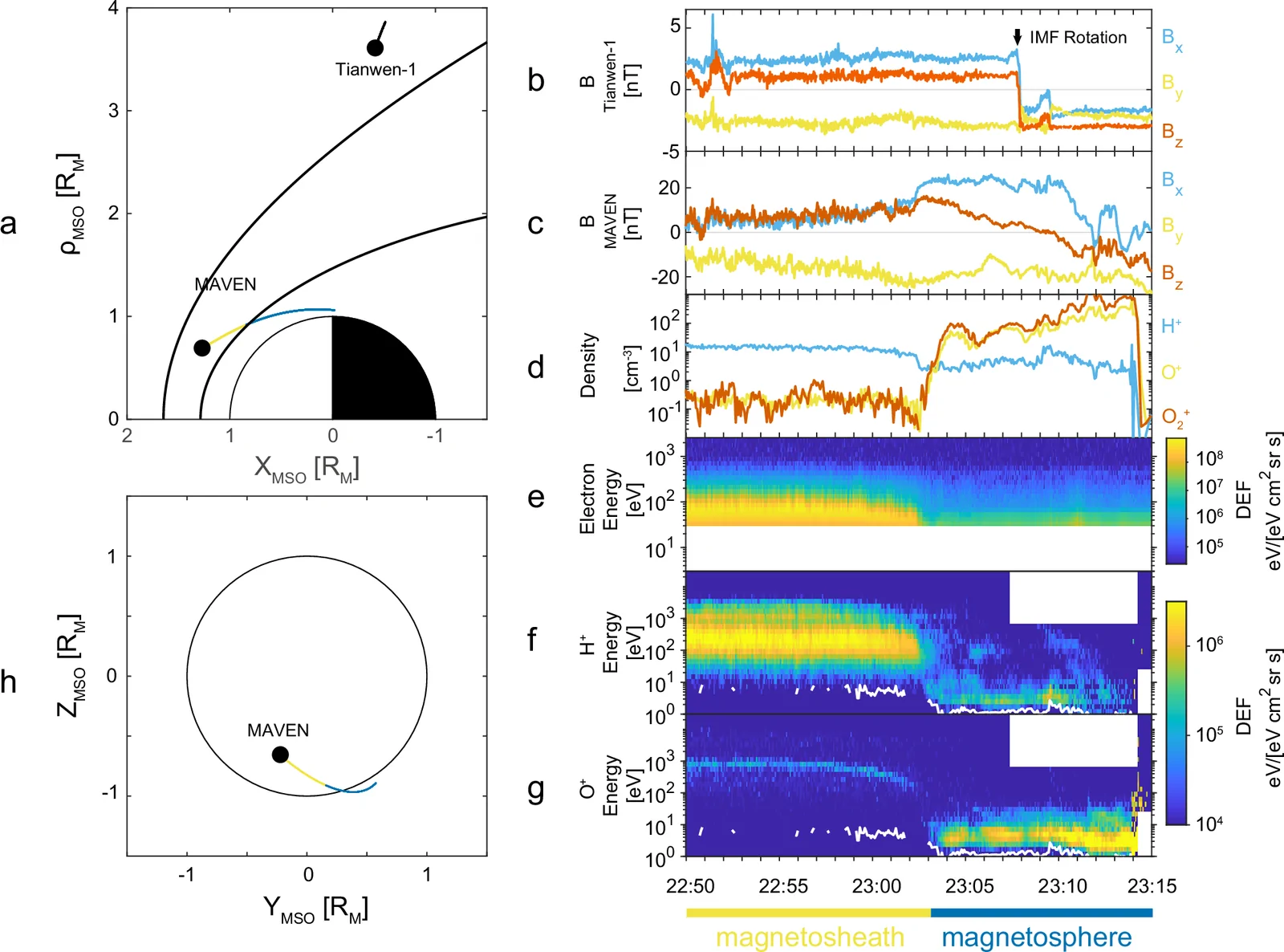
Magnetic field and plasmas measurements by MAVEN and Tianwen-1. **a** Orbits of MAVEN and Tianwen-1 in x-*ρ* plane, where *ρ* is defined as $\sqrt{{y}^{2}+{z}^{2}}$; **b**, **c** magnetic field in MSO coordinates by Tianwen-1 and by MAVEN; **d** ion density; **e** electron spectrogram, **f**, **g** ion spectrograms of H^+^ and O^+^ respectively, and **h** orbit of MAVEN in y-z plane viewed from the sun. MAVEN was crossing the magnetosheath and magnetosphere and Tianwen-1 was in the solar wind on December 23, 2021 during 22:50-23:15 UT. In panel a, the black line represents the orbit in the solar wind, the yellow line represents the orbit in the magnetosheath, and the blue line represents the orbit in the magnetosphere. The two black curved lines represent the bow shock and the induced magnetosphere boundary from the model^71^, respectively. The half of black circle in panel a represents the Mars. Source data are provided as a Source Data file.

Fig. 1 shows magnetic field measurements by Tianwen-1^35,36^ and magnetic field^37^ and plasmas measurements^38,39^ by MAVEN on December 23, 2021, from 22:50–23:15 UT in Mars-solar-orbital (MSO) coordinates. A detailed overview of the instruments on these two orbiters is provided in the “Methods” section. The orbits of MAVEN and Tianwen-1 are also shown in Fig. 1a. In the MSO coordinates, the *X* axis points from Mars toward the Sun, the *Y* axis points opposite to the direction of the orbital velocity component of Mars perpendicular to the *X* axis, and the *Z* axis completes the orthogonal coordinate set. The weak magnetic field observed by Tianwen-1 (Fig. 1b) and the orbit of Tianwen-1 (Fig. 1a) indicate that Tianwen-1 was in the solar wind. Disturbances of the magnetic field and plasma components, such as hot electrons, hot protons and energetic oxygen ions, are observed by MAVEN during 22:50-23:03 UT (Fig. 1c and e–g), indicating that MAVEN was in the magnetosheath (marked as the yellow bar). During 23:03–23:15 UT, the disturbance of the magnetic field was weaker, hot electrons over 50 eV disappeared, the density of the oxygen ions was much greater than that of the protons, and the energy of most of the oxygen ions was under 10 eV (Fig. 1c and e–g), indicating that MAVEN was in the induced magnetosphere (marked as the blue bar).

As shown in Fig. 1b, the IMF remained steady and the magnetic field components [*B*_x_, *B*_y_, *B*_z_] were about [2.30, -2.38, 1.06] nT during 22:50:00–23:07:45 UT; at 23:07:45 UT, the IMF deflected rapidly (the IMF cone angle changed from about 51 degrees to about 67 degrees and the IMF clock angle changed from about 291 degrees to about 213 degrees) and became stable again at and the magnetic field components [*B*_x_, *B*_y_, *B*_z_] were about [-1.94, -1.74, -2.88] nT during 23:08–23:15 UT. When the IMF was rotating (marked as a black arrow in Fig. 1b), the MAVEN orbiter was in Mars’ induced magnetosphere, providing an opportunity to examine its response to the upstream IMF rotation.

Note that two issues should be considered when investigating the response to the upstream IMF rotation. First, the induced magnetosphere is not a simple draped-field system, and the local magnetic-field direction inside the induced magnetosphere is not expected to be identical to the upstream IMF direction even under steady conditions^40–44^. Therefore, the response cannot be determined simply from whether the clock-angle difference between the upstream IMF and the local magnetic field becomes zero in MSO coordinates. Second, this event included regions at relatively low altitude and near the terminator, where local crustal magnetic fields may affect the measured field.

To interpret whether the observed magnetic-field change is consistent with an IMF-driven reconfiguration, we compare the observations with two references.

The first reference is provided by hybrid simulations under the pre- and post-rotation IMF conditions. Two sets of simulations were performed with the RHybrid three-dimensional hybrid simulation platform^45,46^. One set includes the crustal magnetic field, and the other excludes it. Each set consists of two simulation runs corresponding to the pre- and post-IMF rotation states, with the pre-rotation results referring to the period before 23:07:45 UT and the post-rotation results to that after 23:08:00 UT. Detailed descriptions of the hybrid simulation model and initial setups are provided in the “Methods” section.

**Fig. 2:**
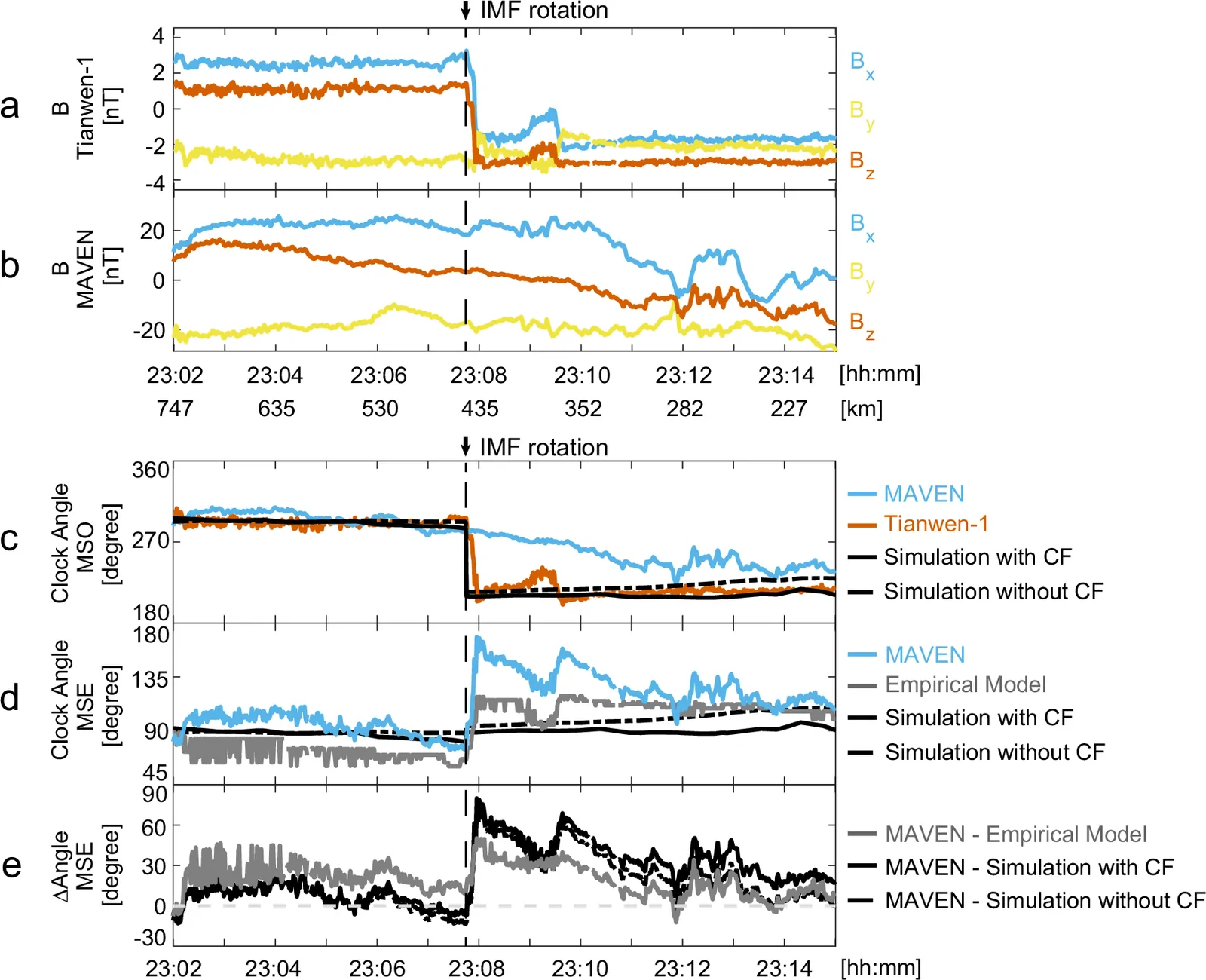
Magnetic field measurements by MAVEN and Tianwen-1 and associated results from the empirical model and the hybrid simulations. MAVEN was in the magnetosphere and Tianwen-1 was in the solar wind. **a**, **b** Magnetic field in MSO coordinates by Tianwen-1 and by MAVEN, **c** clock angle of magnetic field measurements and the hybrid simulations in MSO coordinates, **d** clock angle of MAVEN measurements, the empirical model and the hybrid simulations in MSE coordinates, **e** difference of clock angles in MSE coordinates. In panel c, orange and blue lines represent magnetic field measurements in MSO coordinates by Tianwen-1 and by MAVEN, and the black solid and dashed lines represent magnetic field from the hybrid simulation with crustal fields and that with no crustal fields, respectively, in MSO coordinates. In panel **d** the blue line represents magnetic field measurements by MAVEN in MSE coordinates, the gray line represents magnetic field from the empirical model in MSE coordinates, and the black solid and dashed lines represent magnetic field from the hybrid simulation with crustal fields and that with no crustal fields, respectively, in MSE coordinates. In panel **e** the gray line represents difference of clock angle of magnetic field between MAVEN and the empirical model in MSE coordinates, the black solid and dashed lines represent difference of clock angle of magnetic field between MAVEN and the hybrid simulation with crustal fields and that with no crustal fields, respectively, in MSE coordinates. Source data are provided as a Source Data file.

Fig. 2c shows the clock angles from observations corresponding to the IMF (orange curve) and magnetic field in the induced magnetosphere (blue curve), and from the hybrid simulations (black curves) in MSO coordinates. The clock angle is the azimuthal angle in the y-z plane, starting from the *z* axis in a clockwise direction and ranging from 0 to 360 degrees. A clock angle difference of zero indicates that the magnetic field in the induced magnetosphere is in the same direction as the IMF in the y–z plane. Variations of magnetic field components in each set of simulations are shown in Supplementary Fig. 1. The magnetic field components and the clock angle difference in the simulations all reproduce the change in the magnetospheric magnetic-field direction between the pre- and post-rotation intervals observed by MAVEN (Fig. 2c), indicating that the observed field deflection is consistent with the upstream IMF rotation. The magnetic field lines from simulations in the x-y and x-z planes in MSO coordinates, shown in Supplementary Fig. 2–3, are also consistent with the draping pattern that varies with the IMF rotation. After 23:10:30 UT, the simulation including crustal magnetic fields begins to show a slightly different variation trend from that without crustal magnetic fields (Fig. 2c), indicating that crustal fields can still exert an additional modulation on Mars’ induced magnetosphere. This interpretation is also consistent with the fact that the event occurred over a region with weak crustal magnetic fields (Supplementary Fig. 4).

The second reference is a three-dimensional steady-state empirical magnetic-field model in Mars-solar-electrical (MSE) coordinates, constructed under the statistical constraint of a nearly steady upstream IMF. It provides the expected steady-state magnetic-field configuration of Mars’ induced magnetosphere under a steady upstream IMF orientation. In MSE coordinates, the *X* axis is defined as the direction pointing to the Sun from Mars (assuming that the velocity of solar wind **V**_SW_ points from the Sun to Mars), and the *Z* axis is defined as the direction of the convective electric field vector **E**, where **E** equals to −**V**_SW_ × **B**_IMF_, **B**_IMF_ is the interplanetary magnetic field, and the *Y* axis completes the right-hand system. The details of the model construction and the IMF-dependent sampling procedure are described in the Methods.

Fig. 2d shows comparison between the MAVEN observations, the empirical model and the hybrid simulations in MSE coordinates. When the IMF undergoes rapid variations at 23:07:45 UT, the observed magnetic field in the induced magnetosphere exhibits abrupt changes in MSE coordinates (blue curve in Fig. 2d). This abrupt change does not represent an instantaneous physical reconfiguration of the local magnetospheric magnetic field. Instead, it mainly results from the rapid rotation of the MSE basis vectors associated with the IMF rotation. The steady state of the induced magnetosphere is therefore disrupted. By contrast, the empirical model represents the expected steady-state magnetic field in MSE coordinates and does not reproduce this transient coordinate-driven jump directly (gray curve in Fig. 2d). As the rotated IMF propagates toward Mars, the magnetic field in the induced magnetosphere gradually adjusts toward the new steady-state configuration. This change supports a delayed response of the induced magnetosphere to the upstream IMF rotation. However, since local crustal-field effects cannot be excluded after ~23:10:30 UT, taking the upstream IMF rotation at ~23:07:45 UT as the start, this interval can only provide a lower limit of about 165 seconds for the magnetic recovery time in this case.

In addition, there is a small perturbation in the *B*_x_ component of the IMF between 23:09 and 23:10 UT and a small perturbation in the *B*_x_ component of the magnetic field in the magnetosphere between 23:12 and 23:14 UT. The latter could be the result of the response to the former given the similarity of the two waveforms, which may account for the discrepancy between the model and simulation results and the observations during the interval from 23:12 UT to 23:14 UT.

### Response of the oxygen ion plume to IMF rotation

The oxygen ion plume describes the high-energy fluxes of oxygen ions escaping from Mars over the north pole region in MSE coordinates^7^, where oxygen ions are picked up by solar wind and accelerated in the convective electric field^47^. The convective electric field **E** can be estimated by −**V**_SW_ × **B**_IMF_. The direction of the velocity of solar wind is nearly antiparallel to the *X* axis, whereas the direction of the IMF varies. Thus, the direction of the convective electric field **E** varies mainly with **B**_IMF_, and the direction of the oxygen ion plume follows. The physical picture shows that the direction of the convective electric field changes as the IMF rotates, and an oxygen ion plume appears in the field of view when oxygen ions are picked up in the convective electric field. Thus, the oxygen ion plume is a good indicator of the response of the induced magnetosphere to the rotation of the IMF.

**Fig. 3:**
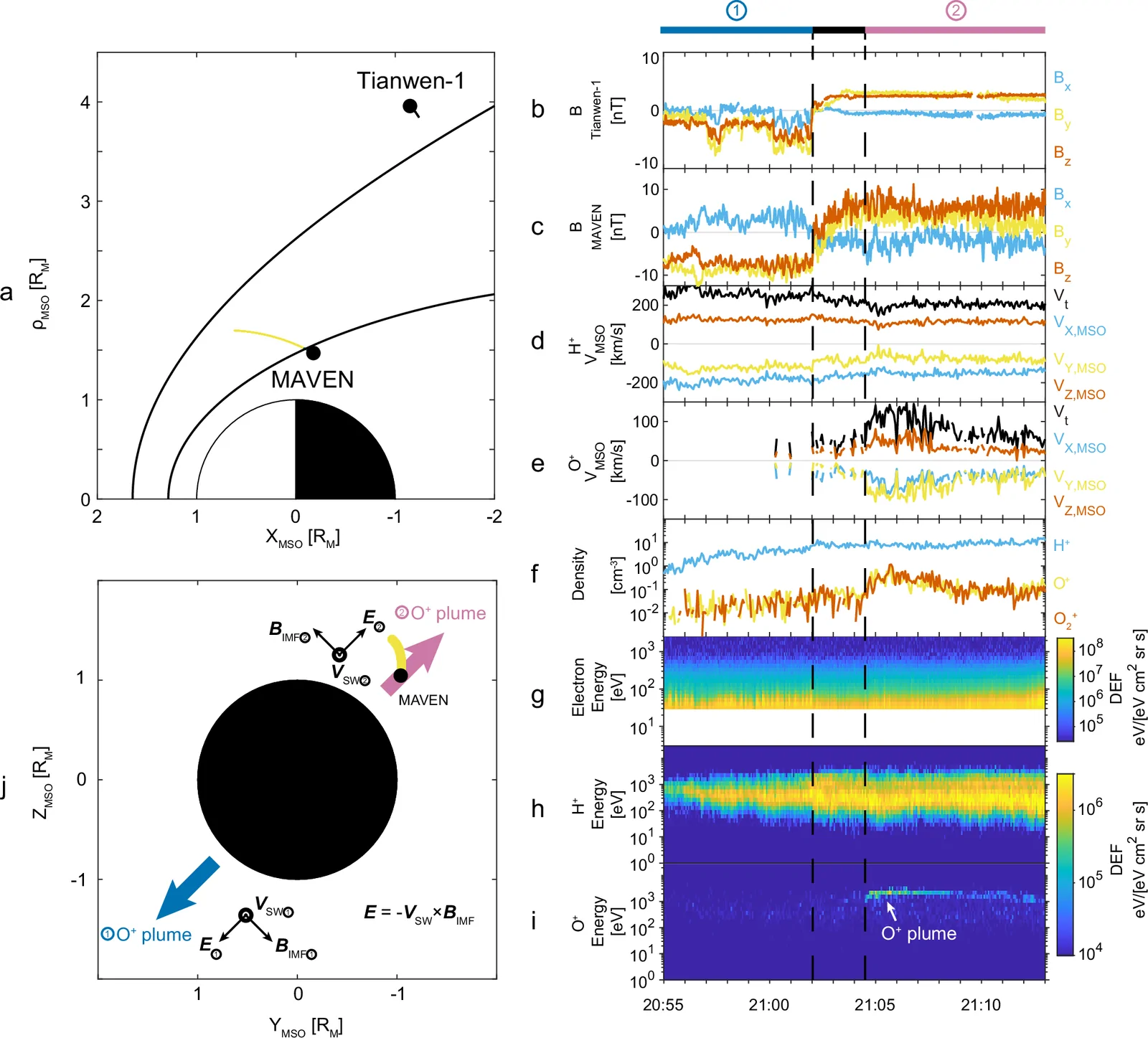
Magnetic field and plasmas measurements by MAVEN and Tianwen-1 on November 16, 2021 during 20:55-21:13 UT. MAVEN was crossing the magnetosheath, and Tianwen-1 was in the solar wind. **a** Orbits of MAVEN and Tianwen-1; **b**, **c** magnetic field in MSO coordinates by Tianwen-1 and by MAVEN; **d**, **e** velocity components of H^+^ and O^+^ in MSO coordinates, **f** ion density; **g** electron spectrogram, **h-i** ion spectrograms of H^+^ and O^+^ respectively, and **j** orbit of MAVEN in y-z plane viewed from the nightside. The colored bars above the figure represent different time periods: the blue one represents period 1, the pink one represents time period 2 and the black one represents the time period in between. Panel j shows theoretically the position of oxygen ion plume resulting from the convective electric field during time period 1 and time period 2, which is colored by blue and pink respectively. In panel **a**, **j** the black line represents the orbit in the solar wind, the yellow line represents the orbit in the magnetosheath, and the blue line represents the orbit in the magnetosphere. Source data are provided as a Source Data file.

Fig. 3 displays an oxygen-ion-plume event that clearly indicates the response of the magnetosphere to the rotation of the IMF on November 16, 2021, from 20:55–21:13 UT. The observations shown in Fig. 3 are in MSO coordinates. The orbit of Tianwen-1 and the observation of a weak magnetic field by Tianwen-1 suggest that Tianwen-1 was present in the solar wind (Fig. 3a–b). The orbit of MAVEN and observations of the hot electrons over 50 eV and the hot protons with energy within 0.1–2.0 keV indicate that MAVEN was in the magnetosheath (Fig. 3a and g–h). During 20:55–21:02 UT, the direction of the IMF observed by Tianwen-1 remains steady at *B*_y_ < 0 & *B*_z_ < 0, and the clock angle is ~222 degrees (marked as period 1; Fig. 3b and j). The direction of the convective electric field here is *E*_y_ > 0 & *E*_z_ < 0 (shown as *E*_1_ in Fig. 3j). The oxygen ion plume should be observed in the region of Y > 0 and Z < 0, which represents the north pole region in MSE coordinates. However, MAVEN was in the southern pole region in MSE coordinates. Therefore, no oxygen ions are detected by MAVEN (Fig. 3i) during 20:55–21:02 UT. At 21:02 UT, the direction of the IMF rapidly changes to *B*_y_ > 0 & *B*_z_ > 0 and becomes steady again where the clock angle is ~45 degrees (marked as period 2; Fig. 3b and j). The direction of the convective electric field here is *E*_y_ < 0 & *E*_z_ > 0 (shown as *E*_2_ in Fig. 3j). The oxygen ion plume should be observed in the region of Y < 0 and Z > 0. Moreover, MAVEN was present in this region, i.e., the north pole region in MSE coordinates. Oxygen ions with typical pick-up energies of ~1 keV were observed from 21:04:30 UT (Fig. 3i), and the velocity direction of the oxygen ions was consistent with that of the convective electric field (*V*_y_ < 0 & *V*_z_ > 0; Fig. 3e).

Since the field of view of STATIC/MAVEN does not cover the full sky, it is necessary to examine whether the observed timing difference of the O^+^ ion plume was caused by limitations of the STATIC viewing geometry. To address this issue, we projected the solar wind electric field direction into the STATIC instrument frame and compared it with the STATIC field of view before and after the IMF rotation (Supplementary Fig. 5). It shows that the solar wind convection electric field direction remained within the STATIC field of view both before and after the IMF rotation. Therefore, the observed plume onset is unlikely to be caused by a change in viewing geometry.

Considering the rotation of the IMF as the start and the occurrence of energetic oxygen ions as the end, the time for the response of the oxygen ion plume to IMF rotation is ~150 s (marked as the yellow bar above Fig. 3b). We determined the start time of the IMF rotation when the clock angle of the IMF started to significantly change (21:01:43 UT). The end of the IMF rotation occurred when the clock angle of the IMF became stable (21:03:23 UT). When the changing IMF reaches the induced magnetosphere boundary, the magnetospheric magnetic field begins to change under the influence of the IMF. Therefore, we took the time when the changing IMF reached the induced magnetosphere boundary as the beginning of the recovery time. The time lag should also be considered between the time of observation of the IMF by Tianwen-1 and the time of IMF arrival at the boundary of Mars’ induced magnetosphere. The distance in the *X* axis between Tianwen-1 and the bow shock at the subsolar point in this case *D*_sw_ is about 2.83 R_M_, where R_M_ is the radius of Mars and is about 3390 km. The time difference is estimated as about 18 seconds. Thus, the response time of the oxygen ion plume is 168 s.

## Discussion

Our results show that Mars’ induced magnetosphere responds rapidly to upstream IMF changes, and the convective electric field rotates with IMF rotation, closely followed by the emergence of a new oxygen ion plume in less than 3 minutes. This suggests that the induced magnetosphere is a rapidly dynamic system that is highly sensitive to changes in the direction of the external magnetic field. Notably, cases of the response of Mars’ induced magnetosphere to a change in radial IMF conditions^34,48,49^ have not yet been identified, and the discussion here is based on the assumption of a large cone angle of the IMF.

A further question is the timescale required for the magnetospheric magnetic field to evolve to a new steady state. The evolution of the magnetic field is described by the magnetic induction equation

1 $$\frac{\partial {{{\bf{B}}}}}{\partial t}=\nabla \times \left({{{\bf{V}}}}\times {{{\bf{B}}}}\right)-\nabla \times \left({\eta }_{m}\nabla \times {{{\bf{B}}}}\right)$$

where **B** is magnetic field vector, **V** is the convection velocity of the plasmas, the magnetic diffusivity *η*_m_ is defined as *μ*_0_^-1^*σ*^-1^, *σ* is the conductivity and *μ*_0_ is the permeability of vacuum. The first term on the right side of the equation is the magnetic transport/convection term, and the second term on the right side is the magnetic diffusion term. The relative importance of the magnetic transport/convection and diffusion terms can be estimated with their characteristic timescales. The transport/convection time constant τ for the magnetic field is given by *LV*^-1^, where *L* is the characteristic length scale and *V* is the speed of plasma flow. The magnetic diffusion time ${\tau }_{m}$ is on the order of *L*^2^*η*_m_^-1^. The magnetic Reynolds numberτ *R*_m_ is defined as the ratio of the diffusion time to the convection/transport time constant, i.e., *τ*_m_*τ*^-1^ (e.g., refs. ^50,51^). The large magnetic Reynolds number indicates that the magnetic field lines can be considered frozen with the plasma flow, whereas the small magnetic Reynolds number indicates that magnetic diffusion plays a major role. A previous study^52^ performed a data-driven analysis of the magnetic frozen boundary and the magnetic diffusion time constant on the basis of MAVEN measurements. According to their results, the magnetic frozen boundary is at an altitude of ~160 km, indicating that magnetic diffusion plays a major role below this altitude and that the magnetic field lines tend to freeze into the bulk plasma flow at higher altitudes. In our case, the lowest altitude reached by the MAVEN orbiter was 200 km, and the range of magnetic diffusion time constants was 10^4^–10^5 ^s, corresponding to an altitude of 200–300 km. Considering the characteristic length scale of 100 km, the magnetic field lines are likely frozen with the plasma flow if the flow speed is greater than 10 m/s, which is generally satisfied at this altitude^53^. Thus, the transport of the magnetic field is of major concern in this case, and magnetic diffusion could be negligible.

In the induced magnetosphere, magnetic diffusion is usually negligible because of the high conductivity, and the induction equation can be approximated as

2 $$\frac{\partial {{{\bf{B}}}}}{\partial t}=\nabla \times \left({{{\bf{V}}}}\times {{{\bf{B}}}}\right)$$

which indicates that the magnetic flux is approximately frozen into the plasma flow. The response of the oxygen ion plume to IMF rotation is controlled by the reconfiguration of the convection electric field. However, the observed plume response is not determined only by the local field adjustment. It also includes the finite time required for planetary O^+^ ions from lower altitudes to be redirected and transported into the observation region. Therefore, the characteristic transport timescale *T* can be estimated as *SV*^-1^, where *S* is the characteristic transport distance and *V* is the O^+^ ion speed. For Mars, we take the subsolar induced magnetosphere boundary altitude as the characteristic distance and *S* equals to 0.29 R_M_. The typical O^+^ ion speed in Mars’ induced magnetosphere is 2–10 km/s^42^, giving a transport timescale of 98–492 s. Considering an additional about 22 s associated with the difference between the subsolar boundary and the terminator boundary, the estimated timescale becomes 98–514 s.

Assuming that the effects of crustal magnetic fields in the first event are neglected, one could estimate the recovery timescale of the induced magnetosphere in response to IMF rotation by comparing the change in the clock angle between the observations, the empirical model, and the hybrid simulations (Fig. 2e). We estimate the response time from the first time when the clock-angle difference between the observed field and the corresponding steady-state reference field first reaches zero, indicating the first alignment with the corresponding steady-state reference. Using the empirical model and the hybrid simulation without crustal magnetic fields as references, the difference in the clock angles (gray solid curve and black dashed curve in Fig. 2e) increase sharply at 23:07:45 UT, when the IMF rotation is first observed by Tianwen-1 and the MSE basis vectors rotate accordingly, gradually decrease and firstly reach zero at 23:11:49 and 23:11:52 UT, respectively. The response times are estimated as 244-247 seconds. For the simulation including crustal magnetic fields, the clock angle difference remains finite throughout the interval. This persistent offset suggests that the crustal magnetic field introduces an additional local or non-local modulation to the field direction, although it does not dominate the overall IMF-driven transition^54^. These results suggest that the response time of the dayside induced magnetosphere near the dawn-dusk sector is on the order of about 4 minutes, confirming that Mars’ induced magnetosphere responds rapidly to upstream IMF changes. Notably, there exists a time lag between the time of observation of the IMF by Tianwen-1 and the time of IMF arrival at the boundary of Mars’ induced magnetosphere. This propagation delay is the main source of uncertainty in the estimated response timescale. MAVEN encountered the undisturbed solar wind at 21:20–22:20 UT and the Martian magnetosheath at 22:50–23:00 UT. Assuming little change in solar wind conditions, the average speeds of protons during these two episodes, which are 339 km/s and 123 km/s, can be regarded as the plasma speed in the solar wind *V*_sw_ and the magnetosheath *V*_sheath_, respectively. The time difference *T*_diff_ can be estimated by

3 $${T}_{{diff}}=\frac{{D}_{{sw}}}{{V}_{{sw}}}-\frac{{D}_{{sheath}}}{{V}_{{sheath}}}$$

where *D*_sw_ is the distance in the *X* axis between Tianwen-1 and the bow shock at the subsolar point, which is about 2.13 R_M_, and *D*_sheath_ is the distance in the *X* axis between the bow shock and the induced magnetosphere boundary at the subsolar point, which is about 0.36 R_M_. The time difference is estimated as about 11 s. Thus, the revised response time is estimated to be 255-258 s. This timescale is consistent with the recovery time inferred from the theoretical analysis above.

Notably, the method used to estimate the timescale applies not only to this case at Mars but also to those at other unmagnetized planets if the physical nature is the same. However, it should be noted that the recovery timescale derived in this work rests on the premise of negligible crustal magnetic field effects. The contribution of crustal magnetic fields to this event cannot be unambiguously excluded. Accordingly, the recovery timescale presented herein serves as a reference value, and its validity requires verification through more thorough investigations in subsequent work.

Like Mars, Venus and Titan have atmospheres but no global intrinsic magnetic field, and both can form induced magnetospheres in interaction with external magnetic fields and plasma flows^3,55^. Although Venus, Titan, and Mars possess different atmospheres, the physical process by which unmagnetized atmospheres interact with an external magnetic field should be the same. On the basis of the results of this study, we verify that in the region above the altitude of the magnetic frozen boundary, the recovery time of the induced magnetosphere depends on the thickness of the induced magnetosphere and the plasma bulk velocity within the induced magnetosphere. This finding leads to the assumption that the recovery time of the magnetic field within the induced magnetosphere of Venus and Titan should also depend on the thickness of the induced magnetosphere and the plasma bulk velocity. The difference is that the atmospheres of Venus and Titan are denser and have different parameters for both the external magnetic field and the upstream plasma flow. If the thickness of the induced magnetosphere and the corresponding plasma flow velocity can be determined, the recovery time of the induced magnetosphere for Titan and Venus can be estimated via the same method.

Since the induced magnetosphere is affected by the IMF, a change in the direction of the IMF should be considered one of the general types of space weather on Mars. When the direction of the IMF changes, the magnetic fields of the induced magnetosphere and of the mini-magnetosphere over the crustal field change^15^. Numerous physical processes occur subsequently or simultaneously, such as magnetic reconnection at the induced magnetosphere boundary^13^ at the flank^17^ and over the crustal field^13,56–58^, the picking up of planetary ions from Mars^7,47^, and the re-establishment of the induced magnetospheric current system^57^. The rapid response of the induced magnetosphere suggests that these physical processes would have occurred in minutes since IMF rotation, which is significant for understanding global dynamical processes in unmagnetized planets. Moreover, monitoring and short-term forecasting of the IMF upstream of Mars are essential.

Most previous studies of single-orbiter measurements are generally based on the assumption that the IMF remains constant within a single orbit^7,57,58^. However, given that the period of satellites is usually several hours, this assumption is obviously very limited. Our results suggest that Mars’ induced magnetosphere is a rapidly dynamic system that is highly sensitive to changes in the direction of the external interplanetary magnetic field. The change in the interplanetary magnetic field should be considered one of the general types of space weather on Mars, and monitoring and short-term forecasting of the interplanetary magnetic field upstream of Mars are essential. This finding indicates that conclusions from previous studies, such as draping and tail twist asymmetries in the magnetotail, should be reconsidered carefully if the effect of IMF rotation is involved and that the MSE coordinates established without simultaneous observations of the magnetic field in solar wind might not be a good reference for case analysis. In future studies involving the response of the magnetosphere, the rotation of the IMF should be considered. In addition, these results can serve as a reference for the response of induced magnetospheric models of Venus, Titan and other planets without intrinsic magnetic fields, and the proposed methods to estimate the response timescale can also be applied.

## Methods

### Instruments

The data analyzed in this study are from the Tianwen-1 and MAVEN missions. The Tianwen-1 data are particularly from the Mars Orbiter Magnetometer (MOMAG^35,36^), and the MAVEN data are from the Magnetometer (MAG^37^), the Solar Wind Electron Analyzer (SWEA^38^) and the Supra-Thermal And Thermal Ion Composition (STATIC^39^). We used the 2C-level data of MOMAG in this study. MOMAG and MAG both provide 3D magnetic fields with a cadence of 32 vectors s^-1^. The reliability of MOMAG has been verified by Zou et al. ^59^ and Wang et al.^60^. The absolute vector accuracy of data from MAG is 0.05%^37^. SWEA provides electron energy spectrogram and pitch angle distribution at a temporal resolution of 2 s. STATIC provides energy spectrogram of different ions species at a temporal resolution of 4 s. Note that the ion density and velocity vectors are derived from STATIC measurements.

The Tianwen-1 orbiter is in an elliptical orbit with a periapsis altitude of about 270 km and apoapsis altitude of about 11,000 km. And the orbit period of Tianwen-1 is about 8 h. The MAVEN spacecraft is also in an elliptical orbit with a periapsis altitude of about 170 km and apoapsis altitude of about 4400 km. The orbit period of MAVEN is about 4.5 h. Therefore, it is advantageous for Tianwen-1 to take long-term measurements of solar wind.

### Steady-state empirical model of the magnetic field

The empirical model is a three-dimensional steady-state magnetic vector field in MSE coordinates, constructed under the statistical constraint of a nearly steady upstream IMF. It provides the expected steady-state magnetic-field configuration of Mars’ induced magnetosphere in the MSE coordinates. The MAG on MAVEN provided 32 magnetic field vectors per second in the MSO coordinates. To obtain the magnetic field measurements in MSE coordinates, the location and direction of the magnetic field measurements in MSO coordinates are rotated around the X-MSO axis by the negative of the IMF clock angle, which is the IMF azimuthal angle in the Y-Z plane in MSO coordinates. By time averaging 6 years (2015-2020) of those data, we obtain a three-dimensional steady-state empirical model of the magnetic field in the Martian magnetosphere in MSE coordinates, with a grid spacing of 0.10 R_M_. The magnetic field components of the steady-state empirical model in equatorial plane, in the day–night plane, and in the dawn–dusk plane are shown in Supplementary Fig. 6. For detailed descriptions of this model, one can refer to the Supplementary Information.

This three-dimensional steady-state empirical model presents the steady-state magnetic fields at different local times and altitudes in Mars’ induced magnetosphere under stable IMF conditions, which incorporates draping asymmetries and other MSE effects^61^. By subtracting the observed magnetic field from the magnetic field of the empirical model, the difference between the magnetic fields in the time-varying case and those from the steady-state model can be obtained. Theoretically, as long as the empirical model of the magnetic field is sufficiently accurate, the difference does not contain draping asymmetries and other MSE effects but only time-varying information about the magnetic field. Thus, one can analyze the response of the Martian magnetosphere to IMF rotation by analyzing the variation in this difference.

In applying this model to the MAVEN observations, a change in the IMF clock angle does not simply rotate the entire empirical magnetic vector field by the same angle at each fixed model point. Instead, for each MAVEN measurement, the observed upstream IMF is first used to define the corresponding MSE coordinate system. The spacecraft position measured in MSO coordinates is then transformed into this IMF-dependent MSE coordinate system, and the empirical magnetic field is obtained by sampling the model at this transformed MSE position. Therefore, the empirical-model variation should not be interpreted as a simple rotation of a magnetic vector at a fixed point. In the first event, the IMF rotates significantly at 23:07:45 UT. Consequently, the MSE coordinate system is rapidly redefined, and the MAVEN position in MSE coordinates changes rapidly from the hemisphere of Y_MSE_ < 0 to that of Y_MSE_ > 0. This change in the sampled model location leads to the component variations (Supplementary Fig. 7). Note that the empirical model here is used only as a reference for the expected steady-state magnetic-field configuration in the MSE coordinates. Identifying the transition from the non-steady state to the new steady state requires a clear reference for the steady-state magnetic field, which the empirical model provides.

### Three-dimensional hybrid simulation

The present study employs the RHybrid three-dimensional hybrid simulation platform^45,62^. In this model, ions are represented as macroparticles, while electrons are treated as a massless, charge-neutralizing fluid, enabling efficient and self-consistent simulation of ion-scale plasma processes. All simulations are conducted in the MSO coordinate system.

The simulation domain is$-4{{\mbox{R}}}_{{\mbox{M}}}\le {\mbox{X}},{\mbox{Y}},{\mbox{Z}}\le 4{{\mbox{R}}}_{{\mbox{M}}}$, where R_M_ is the Mars radius. The whole domain is partitioned into uniform cubic cells with 110 grids in each dimension, and the simulation time step is 10 ms. The upstream solar wind conditions are set as follows: number density of 3.3 cm^-3^, temperature of 1 × 10^5^ K, and bulk velocity of 360 km/s directed along the -X direction. These solar wind parameters are derived from Tianwen-1 observations obtained in the undisturbed solar wind, and are also consistent with typical solar wind conditions at Mars^46,63,64^. Two simulation cases are conducted, corresponding to the conditions before and after the observed IMF rotation shown in Fig. 2a. The upstream IMF components [*B*_x_, *B*_y_, *B*_z_] are set to [2.30, −2.38, 1.06] nT and [−1.94, −1.74, −2.88] nT, respectively. The model incorporates the crustal fields, based on the 110-order spherical harmonic model^65^.

The simulations contain planetary ions, particularly including O^+^ and O_2_^+^ ions from the ionosphere, and H^+^ and O^+^ ions from the exosphere. The ions from the ionosphere are modeled by emitting ions from a spherical inner boundary at a distance of 250 km above the Mars surface. The emission on the dayside has a cosine dependence on the solar zenith angle and that on the nightside remains constant. It peaks at noon and gradually decreases to 10% of its noon-time peak intensity at the terminator. The ions of exosphere origin are generated by photoionization from the exospheric neutral profiles^45^. The global production rates for each ion species are set to the same values in previous work^41^. There is a spherical, absorbing inner boundary 200 km above the Mars surface and particles reaching this boundary are removed from the system. The total simulation duration is 450 s, and the present study focuses on the simulation results at the simulation time of 400 s. This is after the simulations have all reached the quasi-steady phase and the simulation results no longer change significantly.

### Capabilities and limitations of the employed methods

The first method is to infer whether the magnetic field is recovered by whether the clock angle difference of the magnetic field remains constant. This requires simultaneous magnetic field observations from multiple satellites. If the recovery time is to be estimated, the IMF needs to be stable initially and eventually become stable as well. If an ideal steady-state model is available as a reference, the clock angle difference of the magnetic field should become zero as the magnetosphere recovers. Apparently, this requires a considerably accurate model. To a first approximation, the interaction between Mars and solar wind is a Venus-type interaction in which the interplanetary magnetic field drapes around an unmagnetized planet^50,66–68^. The magnetosphere of Mars can be roughly summarized as an induced magnetosphere with a crustal field or dipole field at a fixed location^1,69^. This study focuses on the induced magnetosphere and not on the crustal field or mini-magnetosphere. Therefore, the influence of the crustal field should be avoided as much as possible in this study. The MSE coordinate, which excludes the effect of crustal fields to some extent, is a very suitable coordinate for analyzing the induced magnetosphere^69^. The empirical magnetic model is created in MSE coordinates and excludes the effect of the crustal field by excluding datasets near the crustal field (see more details in Supplementary Information). The existence between the simulation results and the empirical model supports capability of the model.

To further check the capability of the empirical model of predicting response to IMF variation, the empirical-model result was recalculated over the same interval using the averaged pre-rotation IMF condition as a fixed input, i.e., assuming no IMF rotation occurred. The result shows no abrupt change comparable to that observed near 23:08 UT; only a smooth variation associated with the spacecraft motion through the background field structure remains (Supplementary Fig. 8). This confirms that the transition seen in the original model result is indeed related to the change in IMF orientation, rather than being an artifact of the model itself. Besides, we compared the normalized magnetic-field components in MSE coordinates between the MAVEN observations and the empirical model (Supplementary Fig. 7). It should be noted that a comparison of the absolute values of the magnetic-field components is not informative in this case, because the empirical model only incorporates the direction of the upstream IMF through the coordinate transformation and the IMF magnitude is different between this case and the model. Therefore, we focus primarily on the comparison of the normalized magnetic-field components, with each component normalized by the corresponding total magnetic-field magnitude. The comparison shows that the empirical model captures the steady-state trend of the induced magnetospheric magnetic field reasonably well and provides a useful reference for determining when the system approaches its new steady configuration after the IMF rotation. In addition, the variations in the *B*_x_ and *B*_z_ components from the empirical model are associated with the spacecraft traversal between the eastern and western hemispheres in the MSE coordinates^43^, and the looping magnetic field structure^41^, respectively.

The second method is to estimate the recovery time of the induced magnetosphere by observing the appearance or disappearance of the ion plume in the field of view. This method requires simultaneous observations of the IMF in the solar wind and of the planetary ions in the magnetosheath. Second, one of the spacecrafts must be initially or finally located in a region where the oxygen ion plume can be observed, and the interplanetary magnetic field must be rotated by a certain angle so that the ion plume appears or disappears in the field of view of the spacecraft.

## Supplementary information

Supplementary Information

Transparent Peer Review file

## Source data

Source data

## Data Availability

The Tianwen-1 data are publicly available from CNSA Data Release System (https://moon.bao.ac.cn/mall/MarsDATA) or downloaded from the official website of the MOMAG team (http://space.ustc.edu.cn/dreams/tw1_momag/). The MAVEN data used in this study are publicly available at the website: (https://lasp.colorado.edu/maven/sdc/public/data/sci/mag/l2/) and (https://lasp.colorado.edu/maven/sdc/public/data/sci/sta/l2/). The hybrid simulation data are available at the website: (10.5281/zenodo.19247602). Source data are provided with this paper. The data generated in this study are the same as Source data. Source data are provided with this paper.

## Code availability

MAVEN and Tianwen-1 data are loaded, analyzed, and plotted using MATLAB code package which can be are publicly available from Zenodo^70^.

## References

[1] Acuña, M. H. et al. Magnetic field and plasma observations at Mars: Initial results of the Mars Global Surveyor mission. *Science* **279**, 1676–1680 (1998). 9497279 10.1126/science.279.5357.1676

[2] Bridge, H. S. et al. Mariner V: plasma and magnetic fields observed near Venus. *Science* **158**, 1669–1673 (1967). 17749787 10.1126/science.158.3809.1669

[3] Neubauer, F. M., et al*.* Titan’s magnetospheric interaction. (Eds. Gehrels T. and Matthews M.) 760–787 (Univ. of Arizona Press, Tucson, AZ, 1984).

[4] Luhmann, J. G., Ledvina, S. A. & Russell, C. T. Induced magnetospheres. *Adv. Space Res.* **33**, 11 (2004).

[5] Phillips, J. L., Luhmann, J. G. & Russell, C. T. Magnetic configuration of the Venus magnetosheath. *J. Geophys. Res.* **91**, 7931–7938 (1986).

[6] Zhang, T. L. et al. Hemispheric asymmetry of the magnetic field wrapping pattern in the Venusian magnetotail. *Geophys. Res. Lett.* **37**, L14202 (2010).

[7] Dong, Y. et al. Strong plume fluxes at Mars observed by MAVEN: an important planetary ion escape channel. *Geophys. Res. Lett.* **42**, 8942–8950 (2015).

[8] Ogino, T., Walker, R. J. & Ashour-Abdalla, M. A global magnetohydrodynamic simulation of the response of the magnetosphere to a northward turning of the interplanetary magnetic field. *J. Geophys. Res.* **99**, 11027–11042 (1994).

[9] Sakai, S. et al. Effects of the IMF direction on atmospheric escape from a Mars-like planet under weak intrinsic magnetic field conditions. *J. Geophys. Res.: Space Phys.* **126**, e2020JA028485 (2021).

[10] DiBraccio, G. A. et al. Magnetotail dynamics at Mars: Initial MAVEN observations. *Geophys. Res. Lett.* **42**, 8828–8837 (2015).

[11] Wang, J. et al. Plasma and magnetic-field structures near the Martian induced magnetosphere boundary: I. plasma depletion region and tangential discontinuity. *Astron. Astrophys.* **642**, A34 (2020).

[12] Espley, J. R. The Martian magnetosphere: Areas of unsettled terminology. *J. Geophys. Res.: Space Phys.* **123**, 4521–4525 (2018).

[13] Wang, J. et al. MAVEN observations of magnetic reconnection at Martian induced magnetopause. *Geophys. Res. Lett.* **48**, e2021GL095426 (2021).

[14] Wang, M. et al. A magnetohydrodynamic simulation of the dayside magnetic reconnection between the solar wind and the Martian crustal field. *Astron. Astrophys.* **667**, A41 (2022).

[15] Weber, T. et al. The influence of interplanetary magnetic field direction on Martian crustal magnetic field topology. *Geophys. Res. Lett.* **47**, e2020GL087757 (2020).

[16] Langlais, B., Thébault, E., Houliez, A., Purucker, M. E. & Lillis, R. J. A new model of the crustal magnetic field of Mars using MGS and MAVEN. *J. Geophys. Res.: Planets* **124**, 1542–1569 (2019). 35096494 10.1029/2018JE005854 PMC8793354

[17] Sakai, S. et al. Enhanced ion escape rate during IMF rotation under weak intrinsic magnetic field conditions on a Mars-like planet. *J. Geophys. Res.: Space Phys.* **128**, e2022JA030510 (2023).

[18] Lin, R. T. et al. Direct observations of magnetic reconnections at the magnetopause of the Martian mini-magnetosphere. *Geophys. Res. Lett.* **51**, e2024GL108880 (2024).

[19] Maxwell, J. C.et al. *A Dynamical Theory of the Electromagnetic Field*. *Philosophical Transactions of the Royal Society of London*:459 (1865).

[20] Neubauer, F. et al. Titan’s near magnetotail from magnetic field and plasma observations and modelling: Cassini flybys TA, TB and T3. *J. Geophys. Res.* **111**, A10220 (2006).

[21] Ma, Y. et al. Time-dependent global MHD simulations of Cassini T32 flyby: From magnetosphere to magnetosheath. *J. Geophys. Res.* **114**, A03204 (2009).

[22] Snowden, D., Winglee, R. & Kidder, A. Titan at the edge: 2. A global simulation of Titan exiting and reentering Saturn’s magnetosphere at 13:16 Saturn local time. *J. Geophys. Res.* **116**, A08230 (2011).

[23] Simon, S. et al. Titan’s highly dynamic magnetic environment: a systematic survey of Cassini magnetometer observations from flybys TA–T62. *Planet. Space Sci.* **58**, 1230–1251 (2010).

[24] Romanelli, N. et al. Effects of the crustal magnetic fields and changes in the IMF orientation on the magnetosphere of Mars: MAVEN observations and LatHyS results. *J. Geophys. Res.: Space Phys.* **123**, 5315–5333 (2018).

[25] Romanelli, N. et al. Recovery timescales of the dayside Martian magnetosphere to IMF variability. *Geophys. Res. Lett.* **46**, 10977–10986 (2019).

[26] Modolo, R., Chanteur, G. M. & Dubinin, E. Dynamic Martian magnetosphere: Transient twist induced by a rotation of the IMF. *Geophys. Res. Lett.* **39**, L01106 (2012).

[27] Xu, Q. et al. The Dependence of the venusian induced magnetosphere on the interplanetary magnetic field: an MHD study. *Astrophys. J.* **931**, 95 (2022).

[28] Bertucci, C. et al. The magnetic memory of Titan’s ionized atmosphere. *Science* **321**, 1475–1478 (2008). 18787164 10.1126/science.1159780

[29] Wan, W. X., Wang, C., Li, C. L. & Wei, Y. China’s first mission to Mars. *Nat. Astron.* **4**, 721 (2020a).

[30] Wan, W. X., Wang, C., Li, C. L., Wei, Y. & Liu, J. J. The payloads of planetary physics research onboard China’s First Mars Mission (Tianwen-1). *Earth Planet. Phys.* **4**, 331–332 (2020b).

[31] Jakosky, B. M. et al. The Mars Atmosphere and Volatile Evolution (MAVEN) mission. *Space Sci. Rev.* **195**, 3–48 (2015).

[32] Cheng, L. et al. Martian bow shock oscillations driven by solar wind variations: Simultaneous observations from Tianwen-1 and MAVEN. *Geophys. Res. Lett.* **50**, e2023GL104769 (2023).

[33] Wang, Y., Shiyong H., Rentong L. & Zhigang Y. Joint observations by Tianwen-1 and MAVEN: Ushering in a new era of rapid dynamical coupling in the Martian space environment. *Innovation (Camb)* **7**, 101366 (2026). 10.1016/j.xinn.2026.101366 PMC13237833 42254970

[34] Lin, R. T. et al. Mars’ induced magnetosphere can form under radial interplanetary magnetic field. *Innovation* **7**, 101312 (2026). 42254972 10.1016/j.xinn.2026.101312 PMC13237844

[35] Liu, K. et al. Mars orbiter magnetometer of China’s First Mars Mission Tianwen-1. *Earth Planet. Phys.* **4**, 384–389 (2020).

[36] Wang, Y. M. et al. The Mars orbiter magnetometer of Tianwen-1: in-flight performance and first science results. *Earth Planet. Phys.* **7**, 216–228 (2023).

[37] Connerney, J. E. P. et al. The MAVEN magnetic field investigation. *Space Sci. Rev.* **195**, 257–291 (2015).

[38] Mitchell, D. et al. The MAVEN solar wind electron analyzer. *Space Sci. Rev.* **200**, 495–528 (2016).

[39] McFadden, J. P. et al. MAVEN SupraThermal and Thermal Ion Composition (STATIC) instrument. *Space Sci. Rev.* **195**, 199–256 (2015).

[40] Brain, D. A., Mitchell, D. L. & Halekas, J. S. The magnetic field draping direction at Mars from April 1999 through August 2004. *Icarus* **182**, 2 (2006).

[41] Chai, L. et al. The induced global looping magnetic field on Mars. *Astrophys. J.* **871**, L27 (2019).

[42] Dubinin, E. et al. Induced magnetic fields and plasma motions in the inner part of the Martian magnetosphere. *J. Geophys. Res.: Space Phys.* **126**, e2021JA029542 (2021).

[43] Zhang, C. et al. Three-dimensional configuration of induced magnetic fields around Mars. *J. Geophys. Res.: Planets* **127**, e2022JE007334 (2022).

[44] Azari, A. R. et al. Magnetic field draping in induced magnetospheres: evidence from the MAVEN mission to Mars. *J. Geophys. Res.: Space Phys.* **128**, e2023JA031546 (2023).

[45] Jarvinen, R. et al. Oxygen ion energization at Mars: comparison of MAVEN and Mars Express Observations to Global Hybrid Simulation. *J. Geophys. Res.: Space Phys.* **123**, 1678–1689 (2018).

[46] Zhou, J. et al. Influence of the Martian crustal magnetic fields on oxygen ion escape at Mars. *Astron. Astrophys.* **706**, A156 (2026).

[47] Ma, X. et al. Tianwen-1 and MAVEN observations of Martian oxygen ion plumes. *Icarus* **406**, 115758 (2023).

[48] Fowler, C. M. et al. A MAVEN case study of radial IMF at Mars: impacts on the dayside ionosphere. *J. Geophys. Res.: Space Phys.* **127**, e2022JA030726 (2022).

[49] Zhang, Q. et al. Mars’s induced magnetosphere can degenerate. *Nature* **634**, 45–47 (2024). 39294383 10.1038/s41586-024-07959-z PMC11446820

[50] Luhmann, J. G. & Cravens, T. E. Magnetic fields in the ionosphere of Venus. *Space Sci. Rev.* **55**, 201–274 (1991).

[51] Cravens, T. E. et al. Dynamical and magnetic field time constants for Titan’s ionosphere: Empirical estimates and comparisons with Venus. *J. Geophys. Res.* **115**, A08319 (2010).

[52] Cao, Y. T. et al. Characteristic timescales for the dayside martian ionosphere: chemistry, diffusion, and magnetization. *Astrophys. J.* **166**, 264 (2023).

[53] Ma, Y. J., Fang, X., Nagy, A. F., Russell, C. T. & Toth, G. Martian ionospheric responses to dynamic pressure enhancements in the solar wind. *J. Geophys. Res.: Space Phys.* **119**, 1272–1286 (2014).

[54] Dubinin, E. et al. Different faces of the Martian magnetosphere. *J. Geophys. Res.: Space Phys.* **130**, e2025JA033717 (2025.

[55] Phillips, J. L. & Russell, C. T. Upper limit on the intrinsic magnetic field of Venus. *J. Geophys. Res.* **92**, 2253–2263 (1987).

[56] Lin, R. T. et al. Observation of Interchange reconnection in Mars. *Astrophys. J.* **960**, 68 (2024).

[57] Ramstad, R. et al. The global current systems of the Martian induced magnetosphere. *Nat. Astron.* **4**, 979–985 (2020).

[58] Lin, R. T. et al. Characteristics of energetic oxygen ions escaping from Mars: MAVEN observations. *J. Geophys. Res.: Space Phys.* **126**, e2021JA029507 (2021).

[59] Zou, Z. et al. In-flight calibration of the magnetometer on the Mars orbiter of Tianwen-1. *Sci. China Technol. Sci.* **66**, 2396–2405 (2023).

[60] Wang, G. et al. Calibration of the zero offset of the Fluxgate Magnetometer on board the Tianwen-1 orbiter in the Martian Magnetosheath. *J. Geophys. Res.: Space Phys.* **129**, e2023JA031757 (2024).

[61] Liemohn, M. W. et al. Ionospheric control of the dawn-dusk asymmetry of the Mars magnetotail current sheet. *J. Geophys. Res.: Space Phys.* **122**, 6397–6414 (2017).

[62] Kallio, E. et al. Ultra-low frequency waves in the Hermean magnetosphere: on the role of the morphology of the magnetic field and the foreshock. *Geophys. Res. Lett.* **49**, e2022GL101850 (2022).

[63] Ma, Y. J. et al. Importance of ambipolar electric field in driving ion loss from Mars: results from a multifluid MHD model with the electron pressure equation included. *J. Geophys. Res.: Space Phys.* **124**, 9040–9057 (2019).

[64] Liu, D. et al. Statistical properties of solar wind upstream of Mars: MAVEN observations. *Astrophys. J.* **911**, 113 (2021).

[65] Gao, J. W. et al. A spherical harmonic martian crustal magnetic field model combining data sets of MAVEN and MGS. *Earth Space Sci.* **8**, e2021EA001860 (2021).

[66] Crider, D. H. et al. Mars Global Surveyor observations of solar wind magnetic field draping around Mars. *Space Sci. Rev.* **111**, 203–221 (2004).

[67] Luhmann, J. G. Comparative studies of the solar wind interation with weakly magnetized planets. *Adv. Space Res.* **12**, 191–203 (1992).11538138

[68] Luhmann, J. G. & Brace, L. H. Near-Mars space. *Rev. Geophys.* **29**, 121–140 (1991).

[69] Dubinin, E. et al. Magnetic fields and plasma motions in a hybrid Martian magnetosphere. *J. Geophys. Res.: Space Phys.* **128**, e2022JA030575 (2023).

[70] Lin, R. et al. Direct Observations of Rapid Response of Mars’ Induced Magnetosphere to Interplanetary Magnetic Field Rotation, marc-lin97/NC_observations-of-rapid-response-of-Mars-to-IMF, 10.5281/zenodo.21768250, (2026).

[71] Vignes, D. et al. The solar wind interaction with Mars: locations and shapes of the bow shock and the magnetic pile-up boundary from the observations of the MAG/ER Experiment onboard Mars Global Surveyor. *Geophys. Res. Lett.* **27**, 49–52 (2000).

