# Supplementary material for: Induced magnetosphere of Mars responds fast to interplanetary magnetic field rotation
Source: Nat Commun. 2026 Aug 19;17:8727. doi: 10.1038/s41467-026-76861-1 (PMC13494003; doi:10.1038/s41467-026-76861-1)
Supplement: Supplementary file 1 [file 41467_2026_76861_MOESM1_ESM.pdf]

Supplementary Information for

Induced magnetosphere of Mars responds fast to interplanetary magnetic field rotation

Rentong Lin[1,2,3], Jingyi Zhou[2,3], Shiyong Huang[1†], Zhigang Yuan[1], Yuming Wang[2,3], Honghong Wu[1], Kui Jiang[1], Sibo Xu[1], Zhuxuan Zou[2,3], Zhenpeng Su[2,3], Tielong Zhang[4,5], Yue Dong[1], Qiyang Xiong[1], Huxing Huang[1]

[1]School of Earth and Space Science and Technology, Wuhan University, Wuhan, 430072, People's Republic of China

[2]National Key Laboratory of Deep Space Exploration/School of Earth and Space Sciences, University of Science and Technology of China, Hefei, 230026, China

[3]CAS Center for Excellence in Comparative Planetology/CAS Key Laboratory of Geospace Environment/Mengcheng National Geophysical Observatory, University of Science and Technology of China, Hefei, 230026, China

[4]Institute of Space Science and Applied Technology, Harbin Institute of Technology, Shenzhen 518000, People's Republic of China

[5]Space Research Institute, Austrian Academy of Sciences, A-8042 Graz, Austria

[†]Corresponding author: S. Y. Huang (shiyonghuang@whu.edu.cn)

This PDF file includes:

Description of empirical model

IMF-dependent sampling of the empirical model

Supplementary Figure 1

Supplementary Figure 2

Supplementary Figure 3

Supplementary Figure 4

Supplementary Figure 5

Supplementary Figure 6

Supplementary Figure 7

Supplementary Figure 8

References

Description of the model

We use the magnetic field measurements from the MAVEN orbiter during 2014-2022 as the dataset, divide the Martian space into grids and collect the magnetic field measurements within each grid, and compute the average magnetic field values within each grid in the MSE coordinate, and these average magnetic field vectors constitute the empirical magnetic field model. The grid spacing is 0.10 R_M. It is worth noting that the magnetic field measurements from the MAVEN orbiter are in the MSO coordinate, and we need to convert the MSO coordinate to the MSE coordinate when collecting the magnetic field measurements. The descriptions about MSO coordinate and the MSE coordinate are provided in the section Method in the main text. In order to exclude the effect of crustal fields, the empirical model is obtained in which the magnetic field measurements are removed where the crustal magnetic field magnitude by model[1] at altitude of 185 km (Supplementary Fig. 4) is larger than 50 nT. Measurements of the small cone angle (less than 30 degrees) of IMF degrees are removed from the dataset.

To obtain the MSE coordinate, one needs to obtain the direction of the IMF and that of the velocity of solar wind. The direction of the solar wind is assumed to be that of the sun pointing toward Mars, opposite to the direction of the X-axis. We use the magnetic field vector measurements outside the IMB to estimate the direction of the IMF. The locations of the IMB are provided by Vignes et al. (2000)[2]. The magnetic field measurements outside the IMB are low-pass filtered to avoid small-scale distortions of the draped magnetic field in the magnetosheath as well as the effects of short-term variations in the IMF on the global magnetospheric response. Measurements are identified as collected in the magnetosheath or in the undisturbed

IMF using the MAVEN magnetometer and MAVEN STATIC instruments. The weighted average clock angle of the IMF measured in the solar wind and the magnetosheath is taken as the clock angle of the IMF during the corresponding inbound or outbound orbit. Measurements in the undisturbed IMF are weighted 20 times as much as measurements in the magnetosheath. Besides, measurements outside the nominal IMB are identified as measurements in the undisturbed IMF if the proton bulk velocity is higher than 200 km/s and if the cone angle of proton bulk velocity vector is less than 10 degrees. In order to exclude turbulent sheath regions, the 10-point moving standard deviation of the magnetic field is required to be less than 10% of the moving average values. To improve the accuracy of the map of average magnetic field, magnetic field measurements are excluded from the dataset if the time difference from the most recent low-pass filtered IMF estimate is more than ten minutes and the clock angle difference between the two most recent estimates is also greater than 90 degrees.

There are two typical models of magnetic field in the induced magnetosphere. One is dominant by draping field when the cone angle of IMF is large and the other is dominant by induced magnetic field when the cone angle of IMF is small[3]. It is totally different between these two models and the associated IMF conditions are different. As for the case in our study, the averaged IMF cone angle is 54 degrees, which indicates the draping field is dominant. And it refers to the former model. Therefore, cases of small cone angle or radial IMF should be excluded from the statistical MSE map for that these cases are not consistent with the case in our study. Theoretically, the IMF cone angle in the dataset should be close to 54 degrees as much as possible. However, it is impossible since there are no sufficient data available if we set up a narrow range of IMF cone angle, for example, from 50 to 60 degrees. To compromise, a lower limit of IMF cone angle, i.e., 30 degrees, is set up in this study to exclude the radial IMF cases. We have compared cases with different lower limit of IMF cone angle (not shown here) and there is no much difference of the lower limit of IMF cone angle to be 20 degrees, 30 degrees, or 40 degrees. The statistical results are mostly the same since the number of cases of small IMF cone angle is much smaller than that of large IMF cone angle. The magnetic field measurements are in quite good agreement with the empirical model that excludes the effect of the crustal field, both before and after the IMF deflection. It suggests that the empirical model descripts the steady-state induced magnetosphere well.

IMF-Dependent Sampling of the Empirical MSE Model

When the IMF clock angle changes, two effects occur simultaneously. First, the orientation of the MSE coordinate system changes. Second, the same spacecraft position in MSO coordinates corresponds to a different location in the MSE model. As a result, the model samples different regions of the three-dimensional steady-state magnetic-field structure. The modeled magnetic variation is therefore not equivalent to a simple rotation of the magnetic vector at a fixed point, and the modeled clock angle is not expected to exactly follow the IMF clock angle. This also explains why variations can appear in all three modeled components, including B_x . If one only rotated a magnetic vector at a fixed MSE position, an IMF clock-angle change would mainly affect the B_y and B_z components. However, in the empirical-model comparison used here, the sampled MSE position changes with the IMF direction, and the empirical magnetic field has spatial variations in all three components. Thus, $B_{x,model}$, $B_{y,model}$, and $B_{z,model}$ may all vary depending on the local three-dimensional structure of the model field.

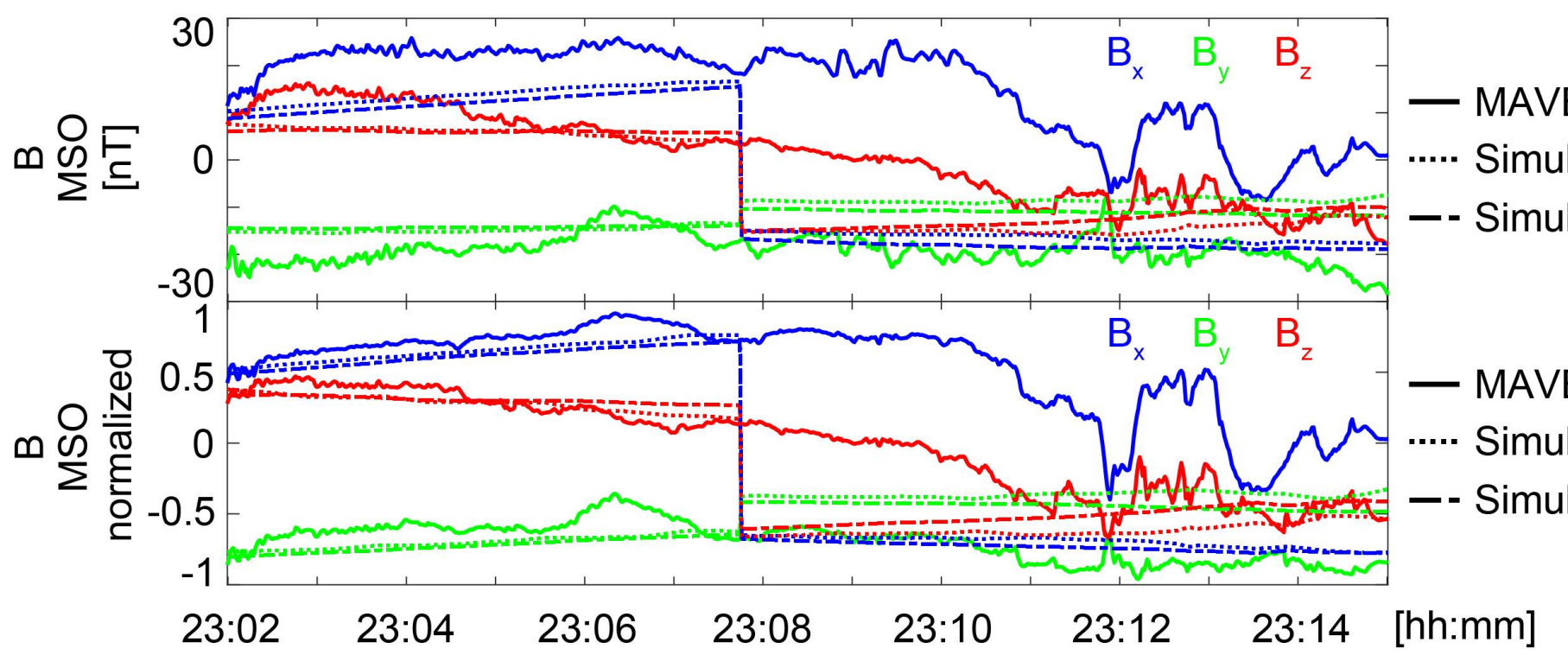

Supplementary Figure 1. Comparison of magnetic field and normalized magnetic field components in MSO coordinates between MAVEN observations and the simulations. The solid curves represent the magnetic field components of MAVEN observations. The dotted curves represent the magnetic field components of the simulation with the crustal magnetic fields. The dashed curves represent the magnetic field components of the simulation without the crustal magnetic fields.

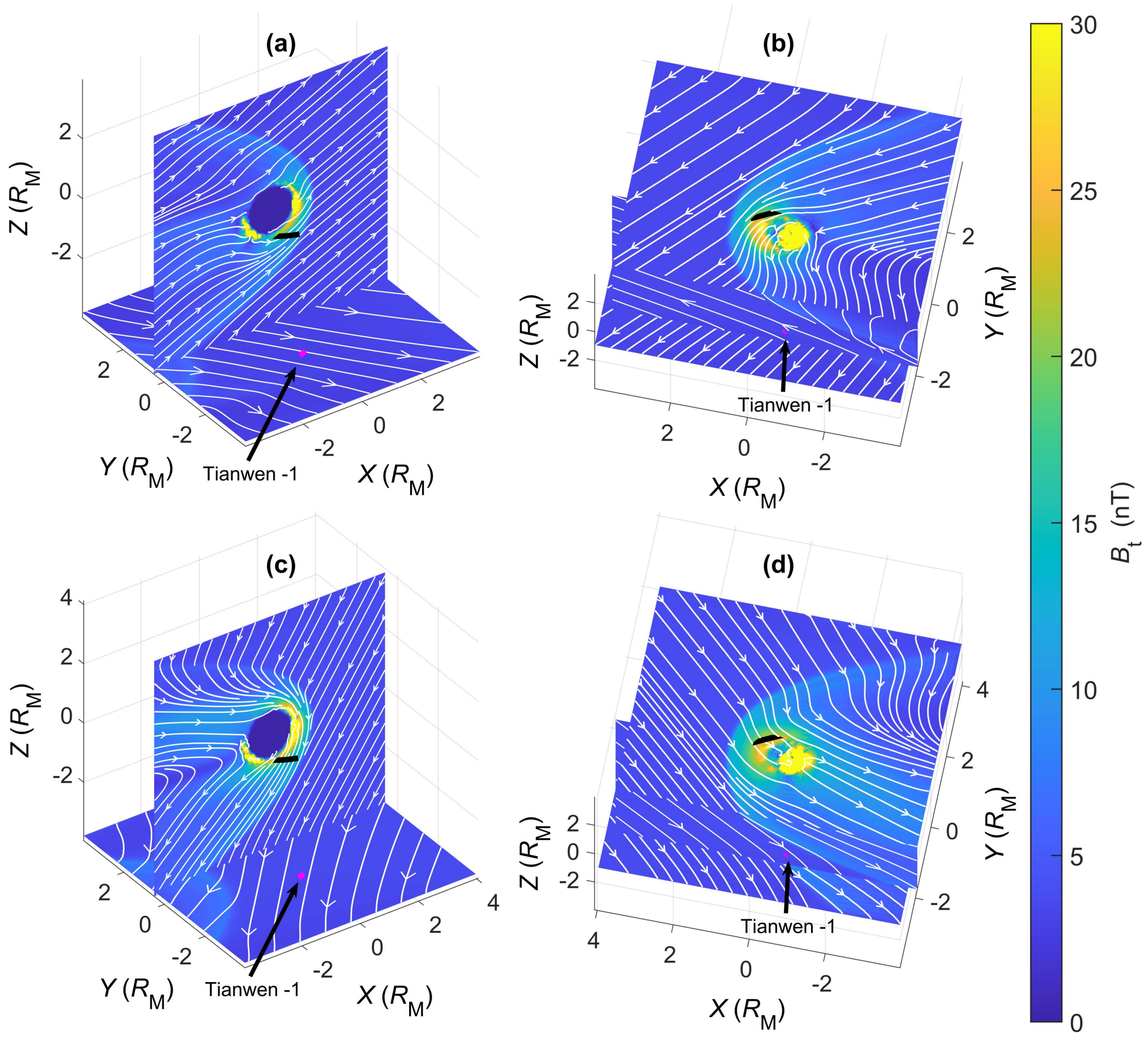

Supplementary Figure 2. **Slices of magnetic field magnitude in the XY and XZ planes in 3D view under two upstream IMF conditions in MSO coordinates. a–b** [B_x, B_y, B_z] equal to [2.30, -2.38, 1.06] nT; **c–d** [B_x, B_y, B_z] equal to [-1.94, -1.74, -2.88] nT. The black and red curves in each panel denote the trajectories of MAVEN and Tianwen-1, respectively. The position of Tianwen-1 is indicated by the black arrow. The white streamlines represent the magnetic field lines. The crustal magnetic field is included in the simulations.

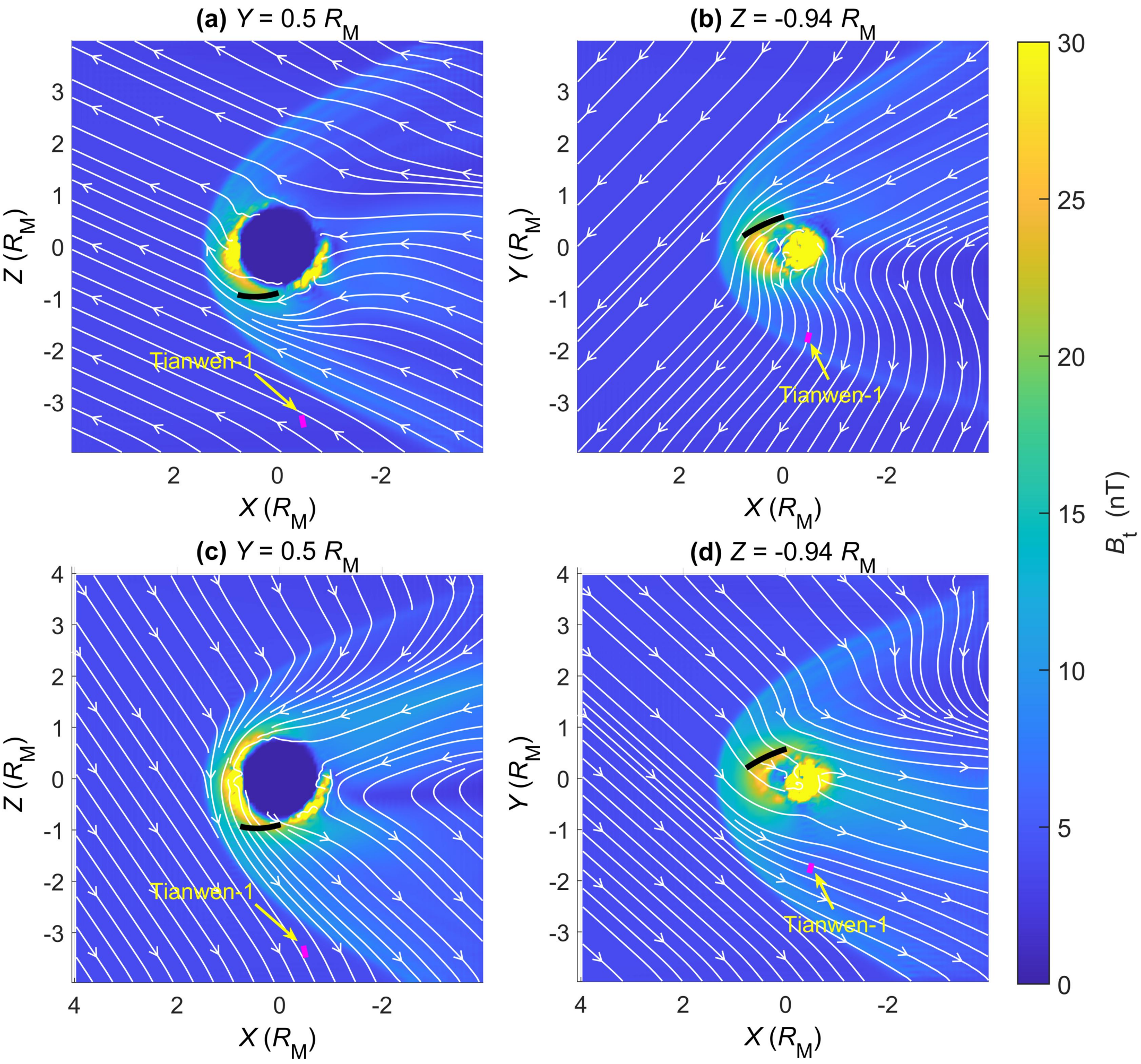

Supplementary Figure 3. **Slices of magnetic field magnitude in the XZ and XY planes under two upstream IMF conditions in MSO coordinates. a–b** $[B_x, B_y, B_z]$ = [2.30, -2.38, 1.06] nT and (c–d) $[B_x, B_y, B_z]$ = [-1.94, -1.74, -2.88] nT. The black and magenta curves in each panel denote the projections of the MAVEN and Tianwen-1 trajectories onto the corresponding planes, respectively. The Tianwen-1 trajectory is indicated by yellow arrows. The white streamlines represent the magnetic field lines. The crustal magnetic field is included in the simulations.

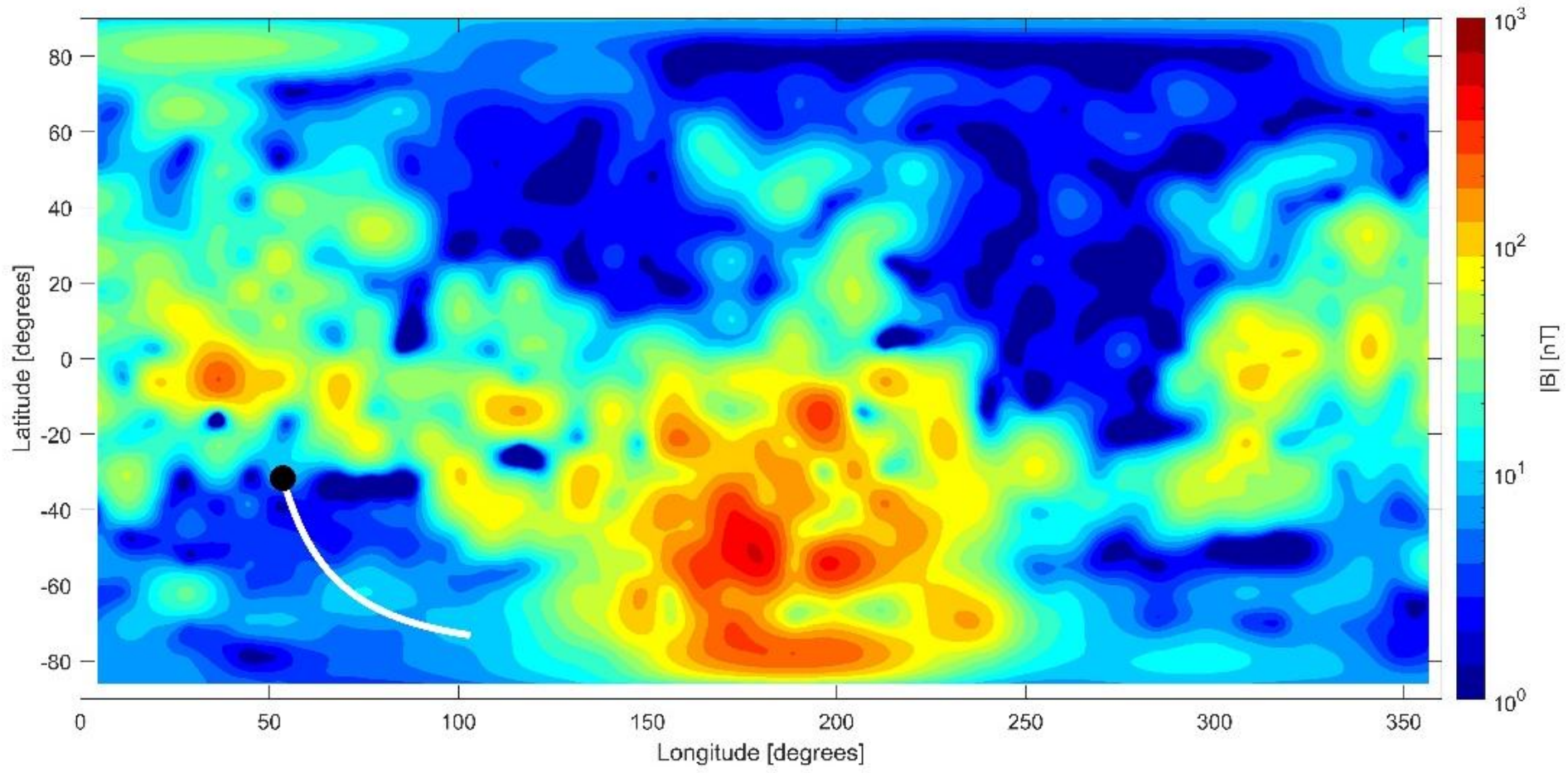

Supplementary Figure 4. **The orbit of MAVEN in the geographic coordinate and the crustal magnetic field magnitude in the time interval of 23:00-23:15 UT on December 23, 2021.** The contour map represents the magnitude of crustal magnetic field at altitude of 185 km by model[2]. The black dot represents the location of MAVEN at 23:00 UT and the white line represents the orbit in the time interval of 23:00-23:15 UT on December 23, 2021.

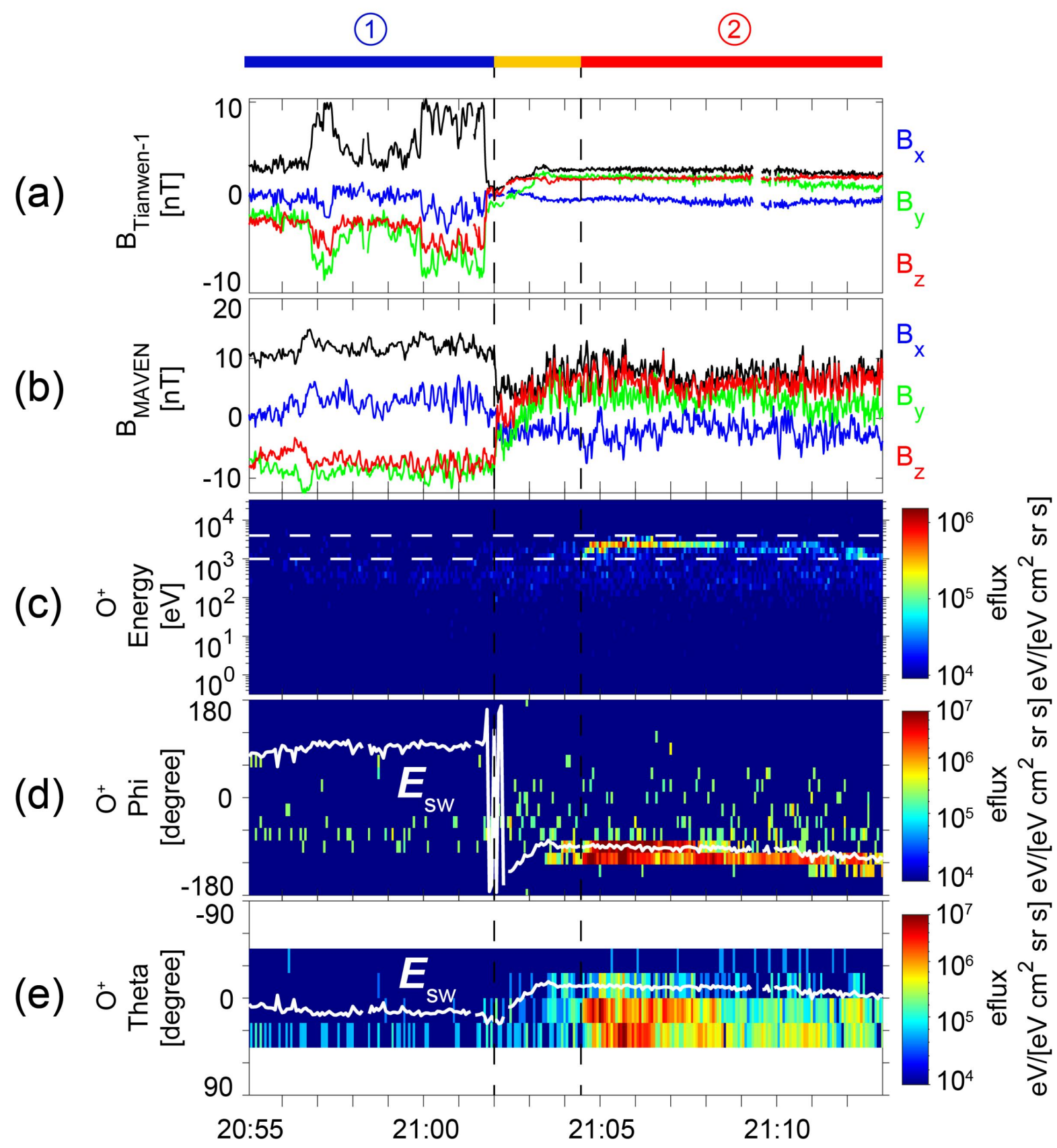

Supplementary Figure 5. The field of view of STATIC/MAVEN and solar wind convective electric field in the time interval of 20:55-21:13 UT on November 16, 2021. The White curves in panel d and e represent the solar wind electric field direction in the STATIC instrument frame.

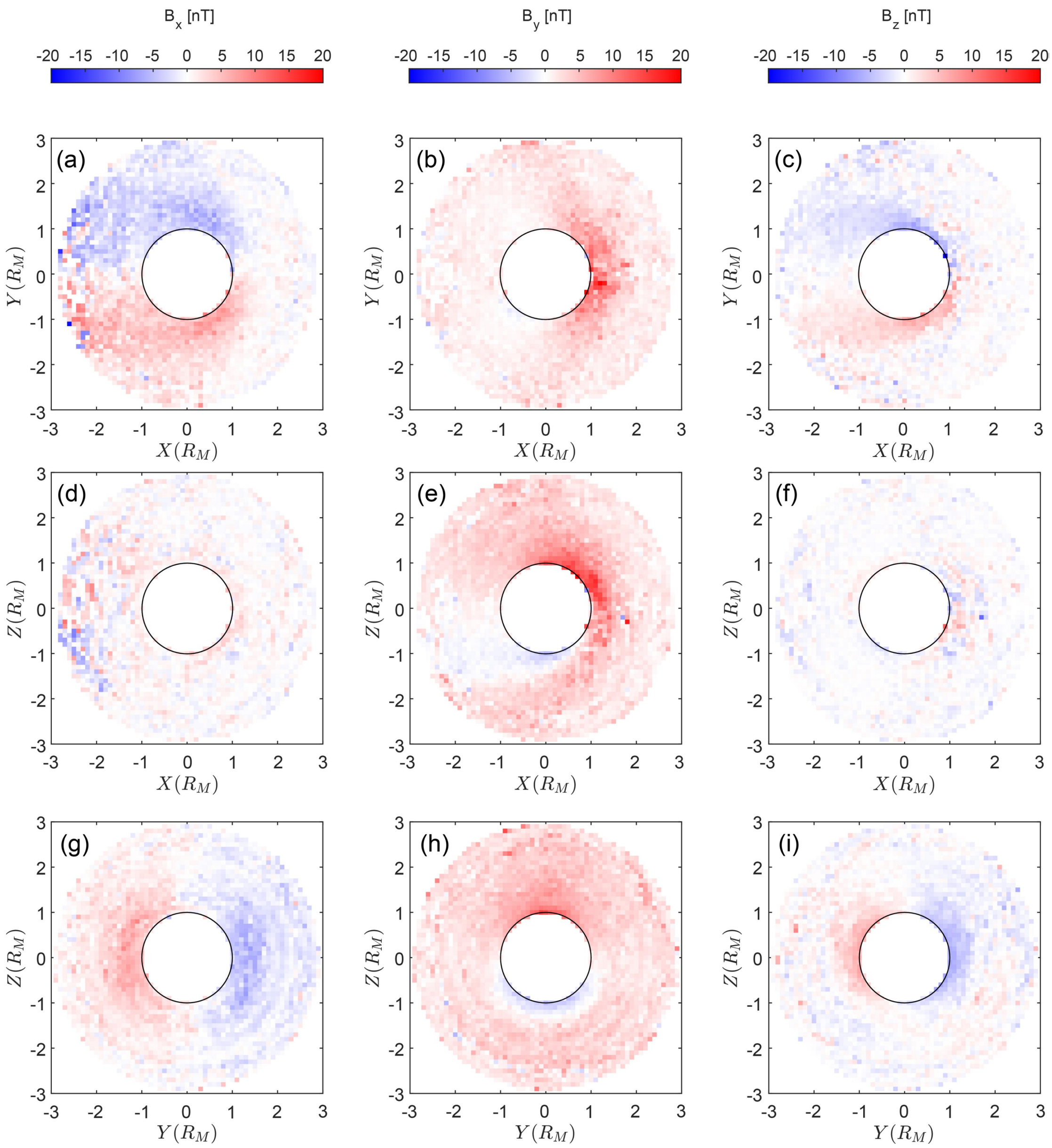

Supplementary Figure 6. **Slices through the steady-state empirical model of magnetic field in the MSE coordinates at Mars.** Three components of magnetic field are shown in equatorial plane (a–c), in the day–night plane (d–f), and in the dawn–dusk plane (g–i). The grid spacing is 0.10 R_M. All slices are 0.10 R_M thick. Each column of panels is colored by the components of the magnetic field in MSE coordinates, respectively. The thin black circle in each panel represents the Mars. The magnetic field measurements were removed from the dataset where the crustal magnetic field magnitude by model[2] at altitude of 185 km is larger than 50 nT.

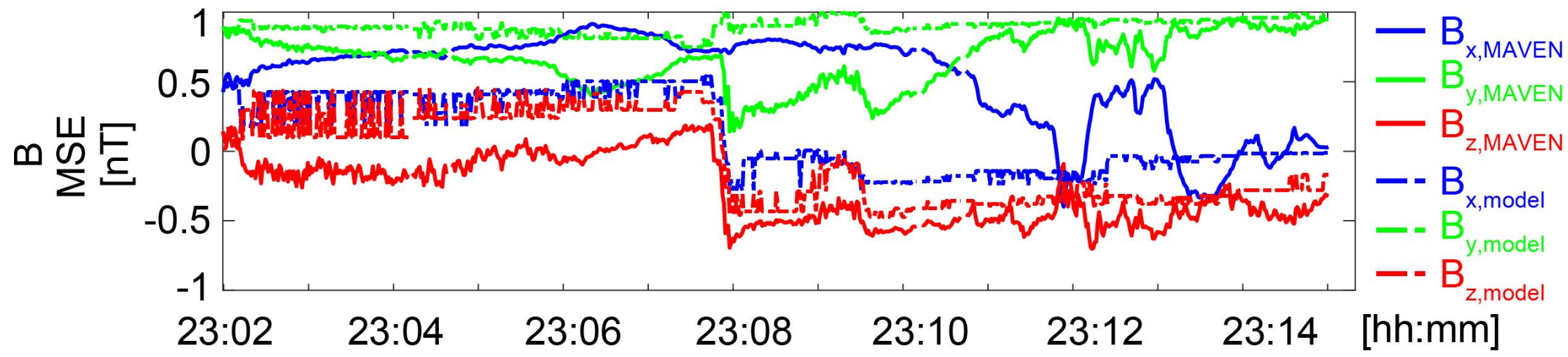

Supplementary Figure 7. Comparison of normalized magnetic field components in MSE coordinates between MAVEN observations and the empirical model. The solid curves represent the magnetic field components of MAVEN observations. The dashed curves represent the magnetic field components of the empirical model.

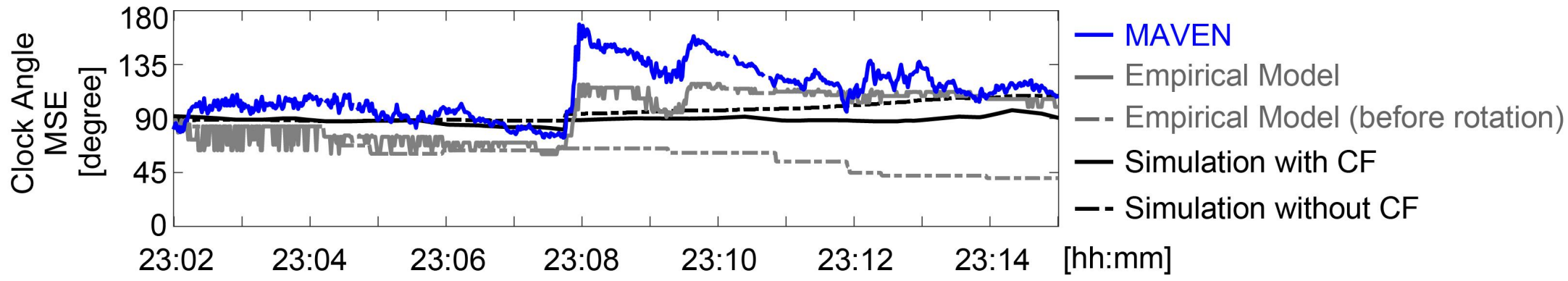

Supplementary Figure 8. Clock angles of magnetic field of MAVEN observations, the empirical model and the hybrid simulations. The blue curve represents MAVEN observations. The grey solid curve represents the empirical model and the grey dashed curve represents the empirical model assuming no IMF rotation. The black solid curve represents the hybrid simulation with crustal magnetic fields and the black dashed curve represents the hybrid simulation with no crustal magnetic fields.

References

1. Morschhauser, A. et al. A spherical harmonic model of the lithospheric magnetic field of Mars. *Journal of Geophysical Research: Planets* 119, 1162–1188 (2014).

2. Vignes, D. et al. The solar wind interaction with Mars: Locations and shapes of the bow shock and the magnetic pile-up boundary from the observations of the MAG/ER Experiment onboard Mars Global Surveyor. *Geophysical Research Letters* 27, 49–52 (2000).

3. Dubinin, E. et al. Toroidal and poloidal magnetic fields at Venus. Venus Express observations. *Planetary and Space Science* 87, 19-29 (2013).
