# Supplementary material for: Induced magnetosphere of Mars responds fast to interplanetary magnetic field rotation
Source: Nat Commun. 2026 Aug 19;17:8727. doi: 10.1038/s41467-026-76861-1 (PMC13494003; doi:10.1038/s41467-026-76861-1)
Supplement: Supplementary file 2 [file 41467_2026_76861_MOESM2_ESM.pdf]

nature portfolio

Peer Review File

Induced magnetosphere of Mars responds fast to interplanetary magnetic field rotation

Corresponding Author: Professor Shiyong Huang

This file contains all reviewer reports in order by version, followed by all author rebuttals in order by version.

Version 0:

Reviewer comments:

Reviewer #1

(Remarks to the Author)
Review of 'Direct Observations of Rapid Response of Martian Induced Magnetosphere to Interplanetary Magnetic Field Rotation' by Rentong Lin et al.,

The study addresses some of the responses of the Martian magnetosphere to changes in the Interplanetary Magnetic Field (IMF), making use of simultaneous observations provided by MAVEN and Tianwen-1 missions. The recovery timescales of the planetary magnetosphere are estimated for two case studies, based on different methodologies: correlation factor, average magnetosphere model in Mars Solar Electric coordinates and the observation of oxygen pick-up ions. I find the manuscript interesting and relevant for the space physics community. Moreover, I think the authors have identified two very useful case studies to investigate the dynamic response of Mars' s magnetosphere to changes in the IMF. However, I also think the manuscript does not sufficiently justify the employed methodology, and therefore the conclusions of this study are questionable. I also think the manuscript would benefit from a more comprehensive discussion comparing the derived conclusions with previous numerical and data analysis studies. Please find below my specific comments for the authors' consideration.

Line 22: I suggest using a different word instead of modulated. Maybe 'affected', 'partly defined'?

Line 22: The interaction is not only with the atmosphere. I suggest adding a mention to the ionosphere too.

Line 29: I suggest adding a bit more information about the IMF rotation. Around what axis does the IMF rotation take place? Does it imply changes in the IMF cone angle, clock angle or both?

Line 29: I am not sure deflected is the right word here. Please check.

Line 32: 'emerged' -> Seen in the spacecraft's field of view?

Line 41: accompanied -> The IMF is convected by the solar wind. I suggest rewriting.

Lines 42: Same comment as for line 22. I would mention the ionosphere too.

Line 46 and rest of the manuscript: 'magnetopause'. The paper cited here mentions the induced magnetosphere boundary, instead of the magnetopause. Also, induced magnetopause is a term that is not used very often in the Martian community. Please see Espley et al 2018 for a discussion on the terminology associated with this boundary (https://doi.org/10.1029/2018JA025278).

Lines 56-63: I think it would be worth adding results from MHD simulations of the Martian magnetosphere too, in addition to the papers mentioned in this paragraph that are focused on Titan.

Line 77: 'in service at' -> orbiting?

Line 82: IMF -> IMF variability.

Line 83: I do not quite understand this sentence. I suggest rewriting it.

Lines 132-137 and Figure 2: I do not think the method involving linear correlation is sufficiently well justified. The correlation factor will be influenced by several factors, not only the IMF rotation. For instance: the magnetic field inside the Martian magnetosphere changes as MAVEN samples different regions inside the induced magnetosphere boundary. Also, I do not think one should necessarily correlate only the Bx MSO component. Is there a justification for this choice? The result will also depend on the size of the window considered to compute the correlation factor. What are the selection criteria on this point? Moreover, what is the interpretation of the other negative correlation factor (local minimum) in panel 2c?

Lines 146-149 and Figure 2: The MSE average employed to compute the steady-state empirical model of magnetic field does not appear to consider other factors, such as variability due crustal field rotation. What about the effects due to local time? Should not be these considered when analyzing two case studies? What is the average Bx of the IMF for all cases used in this averaged picture? Is it consistent with the Bx component shown in Figure 1?

Discussion and conclusions section:
I think there should be more discussion on the capabilities and limitations of the employed methodology. Also, magnetic field diffusion takes place in the collisional ionosphere, increasing recovery timescales and ultimately possibly justifying long diffusion timescales that could affect the recovery timescales. The manuscript would benefit from a discussion on this matter making also reference to other studies.

Also, the sentence in lines 271-275 states that the MSE coordinate is not a good reference frame without simultaneous measurements of the solar wind. However, it seems the authors used a model based on MSE averages derived only from MAVEN data to compute a recovery timescale in Figure 2. This averaged model likely assumes IMF stationary for each MAVEN orbit. Is this correct? If so, please justify why these two arguments are not in contradiction.

Line 185: ‘rises’ -> appears in the field of view.

Line 234: magnetic diffusion could be important at a low altitude.

Figure 2e: The fact that you see changes in the clock angle difference before the IMF rotation takes place appears to be due to MAVEN changing location inside the Martian magnetosphere (panel 2e, red curve, before 23.08 UT). This shows that there are other factors that can affect the linear correlation computed in panel 2c, in addition to the IMF rotation. Please justify why the correlation factor is still reliable despite this and other limitations mentioned in my previous comments.

Figure 2e: The clock angle difference (red curve) never reaches zero in the explored time interval. Could the authors provide a justification for this?

Reviewer #2

(Remarks to the Author)
Summary of review:

The manuscript presents two case studies of Interplanetary Magnetic Field (IMF) rotations upstream of Mars based on MAVEN and Tianwen-1 magnetometer and plasma measurements. The analysis provides estimates respectively for the response time of the dayside induced magnetosphere (<3.5 min), as well as for the energetic plume of ionospheric O^+ scavenged from the ionosphere and picked-up by the solar wind (<3 min).

The study is interesting, the methodology is valid and makes an unusual combined use of multipoint in situ measurements in the Martian system, although it is questionable how big the impact is of a single case study for each phenomenon (magnetosphere and plume), particularly as these mostly appear to confirm previous model results.

Editorially, the writing is mostly concise, though unclear in some places. There are unfortunately too many grammatical errors to be corrected in a scientific review, the manuscript thus requires dedicated proof-reading.

General comments:

The physics of the magnetosphere induction process is largely glossed over in the introduction and simply said to be “modulated” by an external magnetic field. A physical description would provide the wide readership of this journal with the background they need to understand the results.

The readers of this journal would also greatly benefit from a brief overview of the instruments, their capabilities, measured quantities, and the precision/limitations of the measurements, rather than to have to read through the referenced papers.

Along the same topic as above, changes in the magnetic field generated by spacecraft electrical systems can in some cases appear similar to fast IMF rotations in measurements. Have the variable spacecraft field been subtracted from all of the datasets?

The introduction has a lengthy description of Titan and Venus, I am not sure why this is relevant, if there is a reason it should

be clearly stated for the reader.

The reader is referred to the Supplementary Materials for a description of the empirical magnetic field model used in the analysis. However, the supplementary materials document only contains (besides a title page) a figure with slices through the model and a brief caption that mostly describes the figure, not the model. The reader is left to guess e.g. how this model was created, which “empirical” measurements were used, whether it is based on mean values, medians, or another reduction method, whether crustal fields are excluded, as well as whether upstream conditions have been constrained to be similar to the period of the case study. The description needs to be greatly expanded to be useful to the reader. Also, I cannot find any units for the values shown in the supplementary figure.

Considering that the IMF rotation appears to be far from an ideal discontinuity in the November 16 case, the manuscript should clarify for the reader how the time of IMF rotation was determined, as this is crucial for determining the response time.

Line comments:

Line 22: What is a “planet wind”? At first, I thought it could be atmospheric winds, but this is supposed to be something interacting with the atmosphere. A more fitting word should probably be found.

Line 42: What does “directly” mean in this context? The solar wind interaction is generally not collisional, hence this phrasing could be misleading. A physical description would be much clearer.

Line 43: in -> of

Line 78: This phrasing is a bit unclear, several spacecraft have carried magnetometers (Mars probes, Phobos-2, MGS, etc.), but no two magnetometer equipped orbiters have operated simultaneously.

Lines 81-82: “clearly clarify” should be rephrased. Quantify?

Line 83: What is the controversy (disagreement) on this topic? It would be more precise to say that the arrival of Tianwen-1 has enabled such studies.

Line 12: time-dependent

Lines 167-170: There is no need to assume speeds in the solar wind and sheath. These were measured by MAVEN, the data are publicly available and should be used to avoid needless errors.

Line 171 and others similarly: “subsolar” is an adjective. Here, perhaps this is referring to the subsolar point?

Figure 3j: I appreciate the color coding the authors used in this figure, also adding labels with 1 and 2 circled (as above the time-series panels) to the blue and red arrows would also help guide the readers between the panels.

Reviewer #3

(Remarks to the Author)
Please see the attached PDF for the reviewer report.

Version 1:

Reviewer comments:

Reviewer #1

(Remarks to the Author)
I thank the authors for addressing my comments. Although the manuscript is definitely interesting, I find the employed methodology is still not sufficiently justified. Please find below my additional comments.

‘ Line 29: I suggest adding a bit more information about the IMF rotation. Around what axis does the IMF rotation take place? Does it imply changes in the IMF cone angle, clock angle or both? The IMF rotation took place on the X axis and it means the change of IMF clock angle. We referred it as ‘the IMF clock angle changed’ in the revised manuscript.’

Thank you for the added information. However, I believe this sentence is incorrect. The IMF primarily changed in the Bx and Bz components (see Figure 1b). Therefore, the main rotation seems to occur around the Y-axis, which is associated with changes in both the IMF cone and clock angles.

’...And the variation of the Bx component depends mainly on the changing characteristics of the IMF.’

I do not agree with this statement. Bx, just like By and Bz display variability as MAVEN traverses different regions of the Martian magnetosphere. I still think the manuscript does not provide a justification for the employed methodology. Thus, I

find the interpretation and conclusions questionable.

'Lines 146-149 and Figure 2: The MSE average employed to compute the steady-state empirical model of magnetic field does not appear to consider other factors, such as variability due crustal field rotation. What about the effects due to local time? Should not be these considered when analyzing two case studies? What is the average Bx of the IMF for all cases used in this averaged
picture? Is it consistent with the Bx component shown in Figure 1?
Magnetic field measurements above the crustal fields are mostly removed from the dataset of the empirical magnetic field model. About the first case on December 23, 2021, we checked the geographic coordinates corresponding to the event and found that this event occurred over the southern hemisphere but with a very weak crustal field (as shown in the Figure A4). As for the other case on November 16, 2021, the orbiters are at much higher altitude than the areas that are influenced by crustal fields. Thus, the crustal field effect does not affect either the empirical magnetic field model or the results. There are separate datasets for each local time and each altitude. The average of magnetic field in each dataset makes up the model. Therefore, the empirical magnetic field model contains local time effect. We used the average cone angle to represent the average Bx of the IMF. Measurements of the small cone angle (less than 30 degrees) of IMF degrees are removed from the dataset. In the first case shown in the Figure 1, the cone angles of IMF are mostly larger than 45 degrees and the average cone angle is about 54.'

In my opinion, the use of the MSE average map is not sufficiently justified. Firstly, crustal magnetic fields can globally influence the Martian magnetosphere (e.g., Dubinin et al. 2023, DiBraccio et al. 2022). Additionally, there is no clear justification for excluding observations with IMF cone angles smaller than 30 degrees when creating the MSE map. While I understand that foreshock processes might impact the results, this exclusion seems arbitrary. Furthermore, the manuscript lacks a thorough discussion of asymmetries observable in the MSO coordinate system, such as draping or tail twist, that would be missed in the MSE coordinate system.

Reviewer #2

(Remarks to the Author)
The authors have addressed all of the scientific comments from my previous review. Although there are still numerous grammatical errors and some statements with unclear allusion, the paper is scientifically ready for publication upon careful proof-reading and appropriate editorial corrections.

Minor additional comment:

Line 45-47: Note that this only describes the AC component of the electrodynamic interaction, however, only the DC component will be left when averaging over time (such as the "steady-state" magnetic field structure in the empirical model used in the manuscript).

Reviewer #3

(Remarks to the Author)
See attached PDF.

Version 2:

Reviewer comments:

Reviewer #4

(Remarks to the Author)
This study provides two case studies of how the Mars magnetosphere and ion plume respond to the upstream IMF rotation. One case was to characterize the magnetic field response at 300-500 km altitude (dayside ionosphere), and the other case was to characterize the ion plume at >2000 km (magnetosheath). This study concludes the response time for the two cases is about 160-200 s.

Main comments:
Regarding the first case, I have a couple of concerns.
(1) The manuscript states that because this orbit is over a very weak crustal field, the crustal field effect can be ignored. This is not necessarily true when the region of interest is down to 300 km altitude. The figure below is the comparison of the measured magnetic field and the magnetic field components from the MAVEN observations (solid lines) and a crustal field model [Morschhauser et al, 2014, JGRE] (dashed lines). Particularly, in panel 2, the Bz component from the observation (red-solid) matches decently with the modeled Bz component (red-dashed) from 23:10 and onward. The clock angle (tan-

1By/Bz) variation in the data is mainly caused by the change in Bz, as By remains roughly constant at -20 nT for the time of interest. In this case, the Bz change might be mainly because the crustal field starts to dominate, rather than the upstream IMF rotation.

(2) L164-165, L172-173, and Figure 2c: Why does the modeled clock angle in Fig 2c have ~30 deg change in clock angle while the upstream clock angle changed ~100 deg? There are two things to check: (1) what does the model predict for this segment if there is no IMF rotation, i.e., using the IMF values before the rotation for 23:02-23:15; (2) It would be also informative to compare the actual magnetic components in MSE, not just the angles, for both MAVEN and the model.

L257-258: The response time should/could be different for different regions of the Mars magnetosphere, as shown in model results [Romanelli et al., 2018, JGRA]. This statement is too generalized.

L292-294: Not sure why the plume response time is calculated using the magnetic induction equation. MAVEN MAG data show that the magnetic field "stabilizes" within ~1 min in the sheath. If the ion plume does not originate from in situ production but rather from the ionosphere, the time for ions being accelerated from the ionosphere to the observational altitude also needs to be considered. More clarification is needed.

As pointed out by previous reviewers, the manuscript has quite a few grammatical errors.

Regarding Response to Previous Reviewers' Comments:
(1) The discussion using perturbations in Bx to estimate the recovery time, which I assume is about the paragraph in L196-202, is not quite convincing to me either. If to talk about "similar wave forms", the variations in Bx actually have Bx flip signs (Fig2b, or the 2nd panel in the figure above) a couple of times, while the upstream IMF Bx only decreases from -2 nT to ~0 nT and then back to -2 nT, no sign change involved at all (Figure 2a). Also, I assume this is a typo: "a small perturbation in the Bx component of the magnetic field in the magnetosphere between 23:09 and 23:10 UT." It should be "between 23:12 and 23:14 UT"?
(2) Regarding the discussion on crustal field effects, I have mixed feelings about both the reviewer's comments and the authors' responses. Here are my takes. I think some of Reviewer 1's questions/comments are a little nitpicking. But I also disagree with the authors' argument that crustal field effects only take place at locations at or near crustal fields. Dubinin et al. [2023] show crustal fields have a global effect, which could be minimized in MSE coordinates. This is why there is no tail twist in MSE but only in MSO. Furthermore, lots of referenced papers in the response are either pre-MAVEN or mainly focus on the global-scale structures, while low-altitude results are not thoroughly examined. I'm not against using the averaged magnetic field in MSE to approximate the induced field at Mars, but I'm concerned about its validity in low altitudes. Just as pointed out in my main point (1), it is very likely that the variation in Bz is because of crustal fields.

(Figure and more comments included in the attachment)

Reviewer #5

(Remarks to the Author)
The manuscript titled "Direct Observations of the Rapid Response of the Mars' Induced Magnetosphere to an Interplanetary Magnetic Field Rotation" uses simultaneous observations from MAVEN and Tianwen-1 to estimate the response time of Mars's magnetosphere to rotations in the interplanetary magnetic field (IMF). They focus on two case studies in which Tianwen-1 was observing the solar wind and MAVEN was either within the induced magnetosphere or the magnetosheath. In both case studies, Tianwen-1 observed a rotation in the IMF, and MAVEN observed a delayed response. The first case study focuses on the magnetospheric response to this rotation by comparing MAVEN measurements to an empirical model of the induced magnetosphere in the MSE coordinate system. The second case study focused on the O+ plume response measured by MAVEN, as the rotation in the upstream IMF shifted the direction of the convective electric field, and, as a result, rotated the O+ plume into STATIC's field of view.

Reviewer 1:
In the previous iteration of the manuscript, Reviewer 1 voiced concerns mainly regarding the methodology of the empirical model of Mars' induced magnetosphere. They argue that by averaging the magnetic field observations in the MSE coordinate system in order to generate the empirical model, this methodology ignores the global impact of crustal magnetic fields on Mars' induced magnetosphere. In response to this concern, the authors have provided an argument for the use of the MSE coordinate system and the construction of the steady-state empirical model, including showing multiple global empirical maps in which measurements above strong crustal fields have been removed.
After considering both arguments, it is not clear to me that the authors have adequately addressed this concern from Reviewer 1, and I share similar reservations about this methodology. As Reviewer 1 points out, the crustal fields have a global impact of Mars' magnetosphere extends beyond their local sphere of influence (Dubinin et al., 2023, Dibraccio et al., 2022, and Guo et al., 2025 (https://doi.org/10.1029/2024GL114441). The authors have shown that their empirical maps in the MSE coordinate system do not change when "the magnetic field measurements are removed where the crustal magnetic field magnitude by model (Morschhauser et al., 2014, doi:10.1002/2013JE004555) at altitude of 185 km (Figure A2) is larger than 50 nT." If I understand this correctly, the authors have removed all MAVEN MAG measurement that fall directly above these strong crustal field regions. However, the influence of crustal fields also extends in latitude and longitude (Guo et al., 2025, de Oliveira et al., 2021 (https://doi.org/10.1029/2021JA029242)., as well as out in the magnetotail (DiBraccio et al., 2022).

The authors argue that, according the Dubinin et al., (2023), "the crustal field is reconnected to the IMF and thus affects the

original draping field, which means that the effect of the crustal field on the Martian magnetosphere is limited to regions close to the crustal field and, as an important prerequisite, it must be reconnected to the IMF." I would argue that because crustal fields reconnect with the IMF, this ensures that they have an explicitly non-local effect on the draping, since the generation of open field lines, which also drape around the planet, must have one end attached to the crust of the planet, altering the draping pattern of the field lines.
In short, I also have concerns regarding the use of this steady-state empirical model, and do not think this methodology is properly justified. In the current form of this study, the estimate of the response time of the magnetosphere heavily depends on the "agreement vs. disagreement" between MAVEN MAG measurements and this empirical model. This agreement is based on a model that ignores some key physics of the system, and I would argue the difference between measurement and model is likely quite sensitive to factors other than upstream IMF rotation. For instance, even in the small time series shown in Figure 2d, the clock-angular difference between MAVEN and the model exceeds 20 degrees at ~23:05, which is only slightly less than the change measured at 23:08, which theoretically is the solely the result of the IMF rotation. One suggestion to improve the study could be a comparison between MAVEN measurements and an MHD simulation of Mars' magnetosphere under these two different IMF orientations (pre-and post- rotation). This would ensure that the local time and crustal field effects are captured in the study and would enable more convincing methodology.

Reviewer 3:
Reviewer 3 had concerns about syntax. The examples this reviewer provided have been essentially fixed in the new manuscript, but some issues in syntax and language still persist. The paper is readable, so this may be able to be fixed by the editor, but it is still worth noting that there are still several syntax issues that should be resolved.
Reviewer 3 also argued "The solar wind is still disturbed (IMF Bx spike) at 23:09 and MAVEN seems to respond to this at 23:12. This is worth additional text as it's one of the most obvious 'by-eye' correlations."
The authors address this in Lines 198—204. However, I believe there is still a mistake here. The perturbation in the Bx component in the magnetosphere occurs at 23:12-23:13 , not 23:09 as the current manuscript claims.

Version 3:

Reviewer comments:

Reviewer #4

(Remarks to the Author)

Reviewer #5

(Remarks to the Author)
The latest version of this manuscript is an improvement to the previous versions. The inclusion of the hybrid simulations provides a useful reference for the impact of crustal magnetic fields on the low-altitude measurements. Based on these simulations, the authors provide a more convincing argument that the magnetic field rotation measured by MAVEN is the result of the upstream IMF rotation detected by Tianwen-1, rather than an encounter with a crustal field. Furthermore, the inclusion of the magnetic field trace for an unperturbed IMF (Supplementary Figure 10) also demonstrates that the transition in the clock angle in the MSE coordinates predicted by the empirical model is a shift in IMF orientation, rather than being an artifact of the model itself.
That being said, there are still some concerns that remain. These should be considered minor suggestions, but definitely need to be addressed before I would recommend this study for publication.

(1) The details of the global hybrid simulation and the results of the simulations are lacking clarity. Lines 658-659 discuss some of the inputs into the simulation, but these values (solar wind velocity, density, temperature) require some justification. How long was the simulation run for (in simulation time?). Did the simulation reach stability? Was it first run in steady-state before time-accurate mode was introduced? These details should be included in the methodology section.

Furthermore, a figure showing the global simulation output would be useful for readers to check the accuracy and plausibility of the draped magnetic field. This current manuscript only contains spacecraft traces through the simulation, but a global picture of the output for the two IMF cases with a sample MAVEN and Tianwen-1 trajectory should also be included, perhaps in the supplementary information. This could also help demonstrate visually that the crustal fields are not the dominant source of magnetic field within the simulation domain.

(2) The pointing direction/field-of-view of STATIC should be accounted for in the plume example. Given that the inferred response timescale relies on the apparent onset of the plume, the authors should more rigorously demonstrate that this onset is not driven by changes in viewing geometry. For example, a comparison between the expected plume direction and the instrument field of view before and after the IMF rotation could demonstrate that the plume onset was the result of the change in convectional electric field, not a change in viewing geometry.

Line 177: Not sure what you mean by "recovery" here. Should be more specific like "recovery of the draped magnetic field to this rotation"

Lines 601-612: Some typos with "ref" instead of just placing the references.

Version 4:

Reviewer comments:

Reviewer #4

(Remarks to the Author)

Reviewer #5

(Remarks to the Author)
I appreciate the authors' continued efforts to address the my concerns. My specific questions have now been largely answered, and the manuscript contains a substantial amount of additional analysis. However, in addressing my comments and those of the other reviewers, the manuscript has become difficult to follow. In its current form, the main scientific argument regarding the magnetospheric response to the IMF rotation has been obscured by such a large number of qualifications and supplementary analysis and figures.
The current presentation makes the main result more difficult to navigate. In several places, references in the main manuscript to supplementary figures do not provide enough context to explain how those figures support the overall argument being made in the main text. For example, it is not clear to me what Supplementary Figure 6 represents. The vague caption and brief reference in the main text make the figure difficult to interpret, and similar issues arise elsewhere in the Supplementary Information. The Supplementary Information now contains 16 figures, many of which deserve more explanation in the main text, or or require clearer justification for their inclusion as supplementary material.
My concern is now whether the manuscript in its current form presents a coherent and accessible case for its main conclusions. I think the work would benefit from a more substantial restructuring, in which the authors identify the more essential evidence needed to support the central claim, which should be included more in the main text rather than briefly described in the Supplementary Information.
Given the extent of restructuring required, I cannot recommend publication in its current form. I would support either a major revision focused specifically on clarity and prioritization of evidence, or consideration of resubmission to a journal with a format better suited to the length of the study.

Version 5:

Reviewer comments:

Reviewer #4

(Remarks to the Author)
The authors have addressed all of my comments. I have no further comments.

Reviewer #5

(Remarks to the Author)
I would like to reiterate my appreciation for the authors' efforts to address my concerns regarding the structure of the manuscript. This latest version presents a much clearer and more focused argument, and the authors have clearly gone to considerable effort to improve the manuscript's readability. In particular, the removal of the numerous qualifications concerning the influence of crustal fields on the MAVEN measurements has substantially strengthened the study.

I now have only one minor comment regarding a supplementary figure, as described below.

Once this comment has been addressed, I would be happy to recommend the manuscript for publication.

Supplementary Figure 2: The caption states that the red curve represents the Tianwen-1 trajectory, but the trajectory is very difficult to identify in the figure. The figure could be improved by zooming in closer to Mars and more clearly indicating the spacecraft's location. I suggest including multiple 2D slices of the modeled magnetosphere, similar to Supplementary Figure 5, showing the XZ and XY planes with streamlines indicating the magnetic field direction. These views should be provided both before and after the IMF rotation. This would make the simulation results significantly clearer and easier for the reader to interpret.

Open Access This Peer Review File is licensed under a Creative Commons Attribution 4.0 International License, which permits use, sharing, adaptation, distribution and reproduction in any medium or format, as long as you give appropriate credit to the original author(s) and the source, provide a link to the Creative Commons license, and indicate if changes were made.
In cases where reviewers are anonymous, credit should be given to 'Anonymous Referee' and the source.
The images or other third party material in this Peer Review File are included in the article's Creative Commons license, unless indicated otherwise in a credit line to the material. If material is not included in the article's Creative Commons license and your intended use is not permitted by statutory regulation or exceeds the permitted use, you will need to obtain permission directly from the copyright holder.
To view a copy of this license, visit https://creativecommons.org/licenses/by/4.0/

Reply to the Reviewers

We greatly thank the reviewers for the valuable comments/suggestions that we considered in the resubmitted manuscript. All revised parts are marked in red in the text. Detailed answers to the comments are given below:

Reply to Reviewer #1:

Review of 'Direct Observations of Rapid Response of Martian Induced Magnetosphere to Interplanetary Magnetic Field Rotation' by Rentong Lin et al.,

The study addresses some of the responses of the Martian magnetosphere to changes in the Interplanetary Magnetic Field (IMF), making use of simultaneous observations provided by MAVEN and Tianwen-1 missions. The recovery timescales of the planetary magnetosphere are estimated for two case studies, based on different methodologies: correlation factor, average magnetosphere model in Mars Solar Electric coordinates and the observation of oxygen pick-up ions. I find the manuscript interesting and relevant for the space physics community. Moreover, I think the authors have identified two very useful case studies to investigate the dynamic response of Mars' s magnetosphere to changes in the IMF. However, I also think the manuscript does not sufficiently justify the employed methodology, and therefore the conclusions of this study are questionable. I also think the manuscript would benefit from a more comprehensive discussion comparing the derived conclusions with previous numerical and data analysis studies. Please find below my specific comments for the authors' consideration.

Thanks for the referee's suggestions and comments. We added extensive notes to illustrate the details of the employed method, its strengths and limitations, and its applicability to make it clearer in the revised manuscript. And we added detailed discussions comparing the conclusions with previous studies in the revised manuscript.

Line 22: I suggest using a different word instead of modulated. Maybe 'affected', 'partly defined'?

We replaced "modulated" with "affected" in the revised manuscript.

Line 22: The interaction is not only with the atmosphere. I suggest adding a mention to the ionosphere too.

We added ionosphere in the revised manuscript.

Line 29: I suggest adding a bit more information about the IMF rotation. Around what axis does the IMF rotation take place? Does it imply changes in the IMF cone angle, clock angle or both?

The IMF rotation took place on the X axis and it means the change of IMF clock angle. We referred it as 'the IMF clock angle changed' in the revised manuscript.

Line 29: I am not sure deflected is the right word here. Please check.

We replaced 'deflected' with 'rotated' in the manuscript.

Line 32: 'emerged' -> Seen in the spacecraft's field of view?

We replaced "emerged" with "was seen in the spacecraft's field of view" in the revised manuscript.

Line 41: accompanied -> The IMF is convected by the solar wind. I suggest rewriting.

We rewrote the sentence in the revised manuscript.

Lines 42: Same comment as for line 22. I would mention the ionosphere too.

We added ionosphere in the revised manuscript.

Line 46 and rest of the manuscript: 'magnetopause'. The paper cited here mentions the induced magnetosphere boundary, instead of the magnetopause. Also, induced magnetopause is a term that is not used very often in the Martian community. Please see Espley et al 2018 for a discussion on the terminology associated with this boundary (https://doi.org/10.1029/2018JA025278).

We replaced it with 'induced magnetosphere boundary' in the revised manuscript accordingly.

Lines 56-63: I think it would be worth adding results from MHD simulations of the Martian magnetosphere too, in addition to the papers mentioned in this paragraph that are focused on Titan.

We would like to add more results from MHD simulations but we did not find any more previous studies of the Martian magnetosphere on this topic.

Line 77: 'in service at' -> orbiting?

We replaced "in service at" with "orbiting" in the revised manuscript.

Line 82: IMF -> IMF variability.

We replaced "IMF" with "IMF variability" in the revised manuscript.

Line 83: I do not quite understand this sentence. I suggest rewriting it.

We rewrote this sentence in the revised manuscript.

Lines 132-137 and Figure 2: I do not think the method involving linear correlation is sufficiently well justified. The correlation factor will be influenced by several factors, not only the IMF rotation. For instance: the magnetic field inside the Martian magnetosphere changes as MAVEN samples different regions inside the induced magnetosphere boundary. Also, I do not think one should necessarily correlate only the Bx MSO component. Is there a justification for this choice? The result will also depend on the size of the window considered to compute the correlation factor. What are the selection criteria on this point? Moreover, what is the interpretation of the other negative correlation factor (local minimum) in panel 2c?

Detailed descriptions of the linear correlation method are shown below. The true cross-correlation sequence of two jointly stationary random processes, x_n and y_n, is given by

$$R_{xy}(l) = E\{x_{n+l}y_n^*\} = E\{x_n y_{n-l}^*\}$$

where $-\infty < n < \infty$, the asterisk (*) denotes complex conjugation, and E is the expected value operator. One can only estimate the sequence for that, in practice, only a finite segment of one realization of the infinite-length random process is available.

The correlations with no normalization are computed by

$$\hat{R}_{xy}(l) = \begin{cases} \sum_{n=0}^{N-l-1} x_{n+l} y_n^*, l \geq 0 \\ \hat{R}^*{}_{yx}(-l), \quad l < 0 \end{cases}$$

where $l = -(N-1), \cdots, 0, \cdots, N-1$. In general, the correlation function requires normalization to produce an accurate estimate. Normalizes the sequence so that the autocorrelations at zero lag equal one:

$$\hat{R}_{xy,normalized}(l) = \frac{\hat{R}_{xy}(l)}{\sqrt{\hat{R}_{xx}(0)\hat{R}_{yy}(0)}}$$

In this study, x_n is the data series of magnetic field measured by Tianwen-1 and y_n is the data series of magnetic field measured by MAVEN in the time interval of 23:02-23:15 UT on December 23, 2021. The length of both the two data series of magnetic field is 782, i.e., N=782, and $l = -781, \cdots, 0, \cdots, 781$, which represents that the time lag ranges from -781 seconds to +781 seconds.

We use the linear correlation method to illustrate two points. One is that changes in the magnetospheric magnetic field are related to changes in the IMF; the other is a rough estimate of the response time of the magnetospheric field to changes in the IMF. We do not expect to obtain accurate correlation coefficients or recovery times by this method. It is because, as the referee commented, changes in the magnetospheric magnetic field are affected by many factors. The most important factor is that in-situ observations from orbiters have significant temporal and spatial variations over this time scale. This is why we have used a second approach to analyze this event, and in this way a more precise recovery time is estimated. Although the linear correlation method has many drawbacks here, it is still a very effective means of intuitively determining whether there is a correlation between different data sets.

As shown in Figs. 1b and 1c, the main varying components of the IMF are B_x and B_z, while both the B_x and B_z components of the magnetospheric magnetic field have corresponding variations, with the B_x variation being more significant. It should be noted that there exist the interconnected changes in the B_z and B_y components as the satellite traverses different positions in the magnetosphere. The changes in both ultimately reflect the changes in the clock angle. And the variation of the B_x component depends mainly on the changing characteristics of the IMF. Therefore, in the MSO coordinate system, if we want to analyze the correlation between different magnetic field components in terms of waveform characteristics, the B_x component is a more suitable candidate.

There is a lower limit on the size of the time window, and the data within the time window need to include at least the interval of the IMF rotation (e.g., 23:07:45-23:10 UT) and the interval of the magnetospheric magnetic field (e.g., 23:09-23:15 UT). With this condition satisfied, the local maximum of the correlation coefficient appears in the results for different sizes of time windows, and the corresponding time delays are essentially the same (Figure A1-A3). Therefore, although the

value of the correlation coefficient is affected by the size of the time window, the final conclusions are not affected by this.

The local minimum in Figure 2c suggests that there may be a correlation between these two data in terms of waveform characteristics but it requires one of the data series to be inverted. However, the corresponding positive time delay, i.e. $l > 0$, means that the magnetospheric data (measurements from MAVEN) needs to be shifted forward on the time axis. Physically, any correlation coefficient with a positive time lag is meaningless, since it is clear that the magnetospheric magnetic field is influenced by the IMF and not the other way around. We did not consider this in the first-version manuscript and hadn't thought that this would cause misunderstandings. Thus, we decided to delete the results with positive time lag in the revised manuscript in order not to confuse readers.

In the revised manuscript, we added more descriptions about the purpose and analysis of linear correlation method, the reason why we did not correlate the B_y or B_z component in Line 144-152, the selection criteria on the size of window in Line 612-617. The limitations of the method were added in the section Method in the revised manuscript.

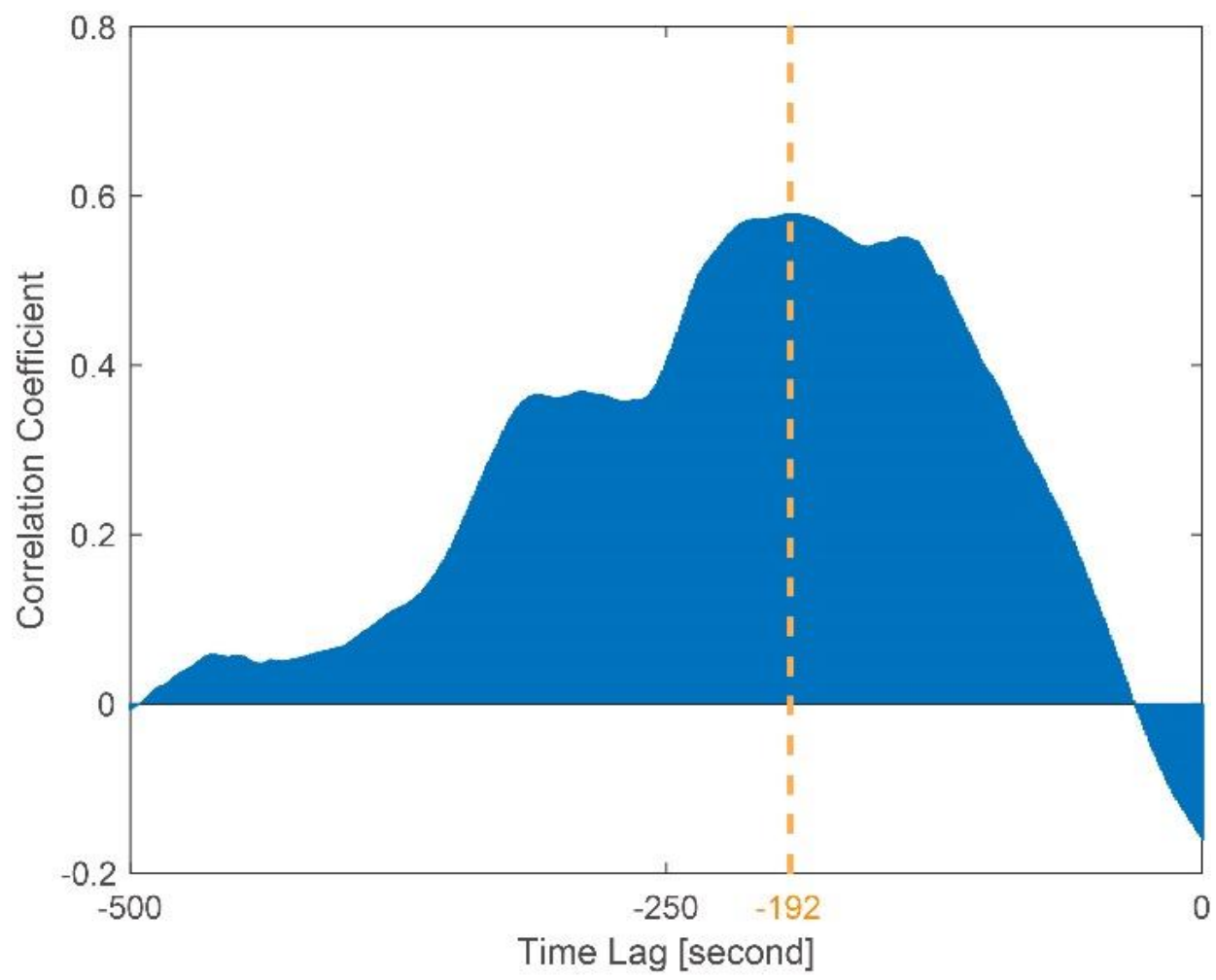

Figure A1. Correlation coefficient between the two magnetic field measurements depended on time lag with the size of window 500 s (i.e., magnetic field measurements by Tianwen-1 from 23:04:31 to 23:12:50 UT and by MAVEN from 23:06:41 to 23:15:00 UT). The orange dashed line marks the peak of correlation coefficient.

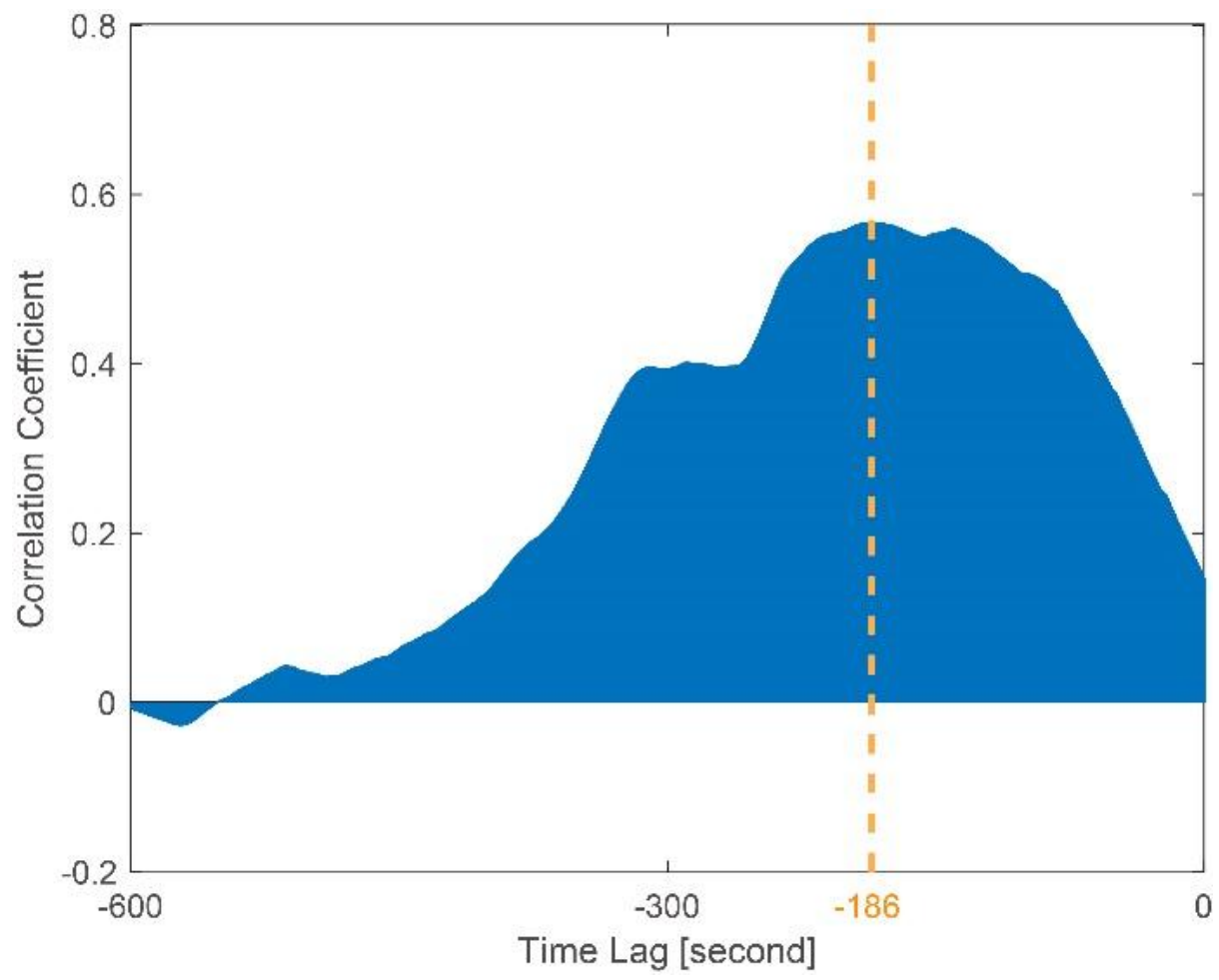

Figure A2. Correlation coefficient between the two magnetic field measurements depended on time lag with the size of window 600 s (i.e., magnetic field measurements by Tianwen-1 from 23:03:41 to 23:13:40 UT and by MAVEN from 23:05:01 to 23:15:00 UT). The orange dashed line marks the peak of correlation coefficient.

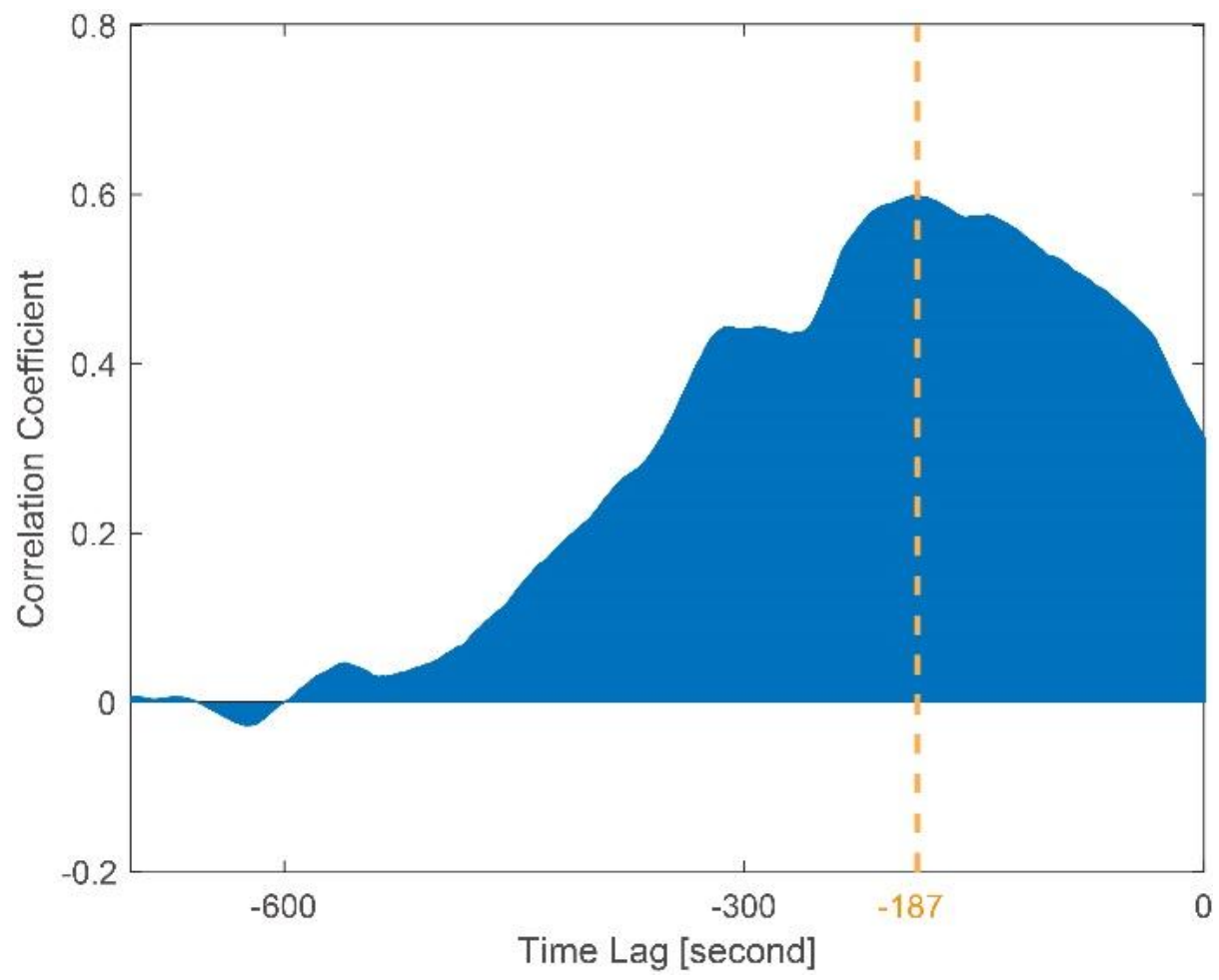

Figure A3. Correlation coefficient between the two magnetic field measurements depended on time lag with the size of window 700 s (i.e., magnetic field measurements by Tianwen-1 from 23:02:51 to 23:14:30 UT and by MAVEN from 23:03:21 to 23:15:00 UT). The orange dashed line marks the peak of correlation coefficient.

References

1. Buck, John R., Michael M. Daniel, and Andrew C. Singer. Computer Explorations in Signals and Systems Using MATLAB®. 2nd Edition. Upper Saddle River, NJ: Prentice Hall, 2002.
2. Stoica, Petre, & Randolph Moses. Spectral Analysis of Signals. Upper Saddle River, NJ: Prentice Hall, 2005.

Lines 146-149 and Figure 2: The MSE average employed to compute the steady-state empirical model of magnetic field does not appear to consider other factors, such as variability due crustal field rotation. What about the effects due to local time? Should not be these considered when analyzing two case studies? What is the average Bx of the IMF for all cases used in this averaged

picture? Is it consistent with the Bx component shown in Figure 1?

Magnetic field measurements above the crustal fields are mostly removed from the dataset of the empirical magnetic field model. About the first case on December 23, 2021, we checked the geographic coordinates corresponding to the event and found that this event occurred over the southern hemisphere but with a very weak crustal field (as shown in the Figure A4). As for the other case on November 16, 2021, the orbiters are at much higher altitude than the areas that are influenced by crustal fields. Thus, the crustal field effect does not affect either the empirical magnetic field model or the results.

There are separate datasets for each local time and each altitude. The average of magnetic field in each dataset makes up the model. Therefore, the empirical magnetic field model contains local time effect.

We used the average cone angle to represent the average B_x of the IMF. Measurements of the small cone angle (less than 30 degrees) of IMF degrees are removed from the dataset. In the first case shown in the Figure 1, the cone angles of IMF are mostly larger than 45 degrees and the average cone angle is about 54 degrees.

More descriptions about the empirical model of magnetic field are provided in section Method and in the Supplementary Information.

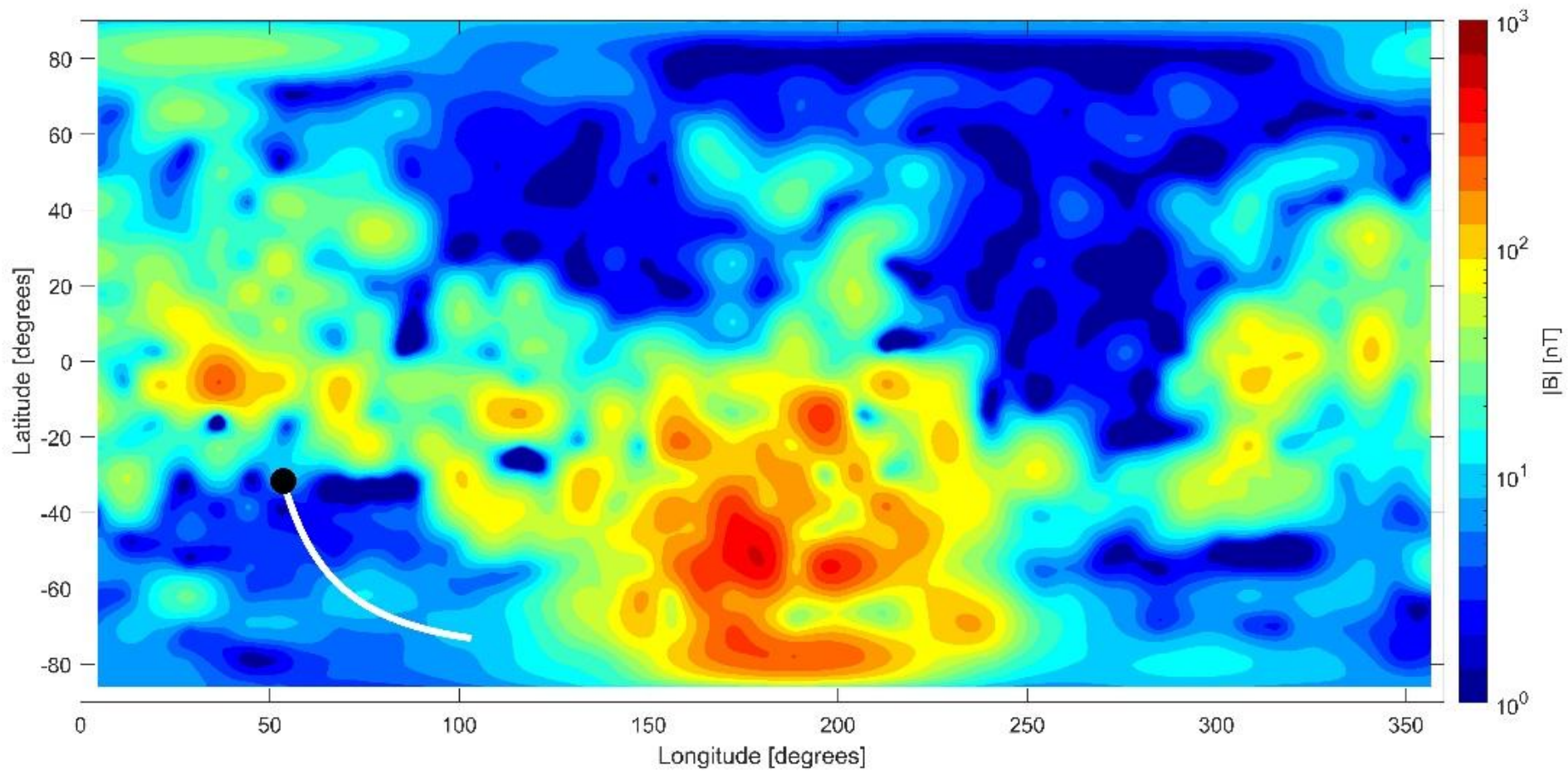

Figure A4. The orbit of MAVEN in the geographic coordinate and the crustal magnetic field magnitude. The contour map represents the magnitude of crustal magnetic field at altitude of 185 km by model (Morschhauser et al. (2014), doi:10.1002/2013JE004555). The black dot represents the location of MAVEN at 23:00 UT and the white line represents the orbit in the time interval of 23:00-23:15 UT on December 23, 2021.

Discussion and conclusions section:

I think there should be more discussion on the capabilities and limitations of the employed methodology. Also, magnetic field diffusion takes place in the collisional ionosphere, increasing recovery timescales and ultimately possibly justifying long diffusion timescales that could affect the

recovery timescales. The manuscript would benefit from a discussion on this matter making also reference to other studies.

We added discussions on the capabilities and limitations of the employed methodology in the section Method and added discussions on the magnetic diffusion in Line 281-304 in the revised manuscript.

Also, the sentence in lines 271-275 states that the MSE coordinate is not a good reference frame without simultaneous measurements of the solar wind. However, it seems the authors used a model based on MSE averages derived only from MAVEN data to compute a recovery timescale in Figure 2. This averaged model likely assumes IMF stationary for each MAVEN orbit. Is this correct? If so, please justify why these two arguments are not in contradiction.

The MSE coordinate is a very effective coordinate system when implementing statistical studies. By transforming measurements to the MSE coordinate, we can see significant large-scale features, e.g., oxygen plume in the +E hemisphere (Dong et al., 2015, doi:10.1002/2015GL065346), looping magnetic field in the magnetotail (Chai et al., 2019, doi:10.3847/2041-8213/aaff6e), current systems in the Martian induced magnetosphere (Ramstad et al., 2020, doi:10.1038/s41550-020-1099-y). These statistical features can be well reproduced by MHD or hybrid simulation results (e.g., Li et al., 2024, doi:10.1029/2024GL109186). But in case analysis, MSE is difficult to use as a good reference, especially when there are only measurements from a single orbiter, for that it is unknown whether the IMF had changed or not when the orbiter was in the ionosphere/ induced magnetosphere. We added more descriptions on this topic in the revised manuscript.

In our study, the empirical magnetic field model is based on the datasets from a single satellite. And these datasets have many limitations, such as removing data of small IMF cone angle (as mentioned above), and the stability of the IMF clock angle within the former and latter orbits. The selection criteria of the datasets are basically the same as Ramstad et al. (2020).

Line 185: 'rises'-> appears in the field of view.

Thanks for the referee's suggestion. We replaced "rises" with "appears in the field of view" in the revised manuscript.

Line 234: magnetic diffusion could be important at a low altitude.

Discussions about magnetic diffusion was added in the revised manuscript.

Figure 2e: The fact that you see changes in the clock angle difference before the IMF rotation takes place appears to be due to MAVEN changing location inside the Martian magnetosphere (panel 2e, red curve, before 23.08 UT). This shows that there are other factors that can affect the linear correlation computed in panel 2c, in addition to the IMF rotation. Please justify why the correlation factor is still reliable despite this and other limitations mentioned in my previous comments.

Thanks for this comment. Detailed explanations can be found in the previous response of the comment of *Line 132-137 and Figure 2*.

Figure 2e: The clock angle difference (red curve) never reaches zero in the explored time interval. Could the authors provide a justification for this?

The difference in clock angles is calculated from the IMF and the magnetic field within the induced

magnetosphere. A clock angle difference equal to zero means that the magnetic field in the induced magnetosphere is in the same direction as the IMF in the y-z plane. Assuming that the induced magnetosphere is a simple draped field model, the magnetic field in the induced magnetosphere on the dayside, with the constant-direction IMF, should always have a clock angle difference of zero. However, previous observations and simulations have shown that the induced magnetosphere is not a simple draped field model (e.g., Brain et al., 2006, doi:10.1016/j.icarus.2005.09.023; Chai et al., 2019, doi:10.3847/2041-8213/aaff6e; Dubinin et al., 2021, doi:10.1029/2021JA029542; Zhang et al., 2022, doi:10.1029/2022JE007334; Li et al., 2024, doi:10.1029/2024GL109186). These studies suggest that there exists angular difference between the direction of the magnetic field in the induced magnetosphere and that of the IMF, implying that it is common and reasonable for the clock angle difference not to be equal to zero. Therefore, it is not possible to determine whether the magnetic field has fully recovered by the clock angle difference being strictly equal to zero. Another factor to take into account is that, as the referee mentioned above, there are also differences between magnetic fields at different spatial locations. To minimize the impact of this factor, we introduce the empirical magnetic field model and more illustrations on this factor and the empirical model have been present in the responses above. In this study, we consider the magnetic field to have recovered when the clock angle difference between the empirical model and the magnetospheric magnetic field remains essentially unchanged (black curve in the time interval of 23:12-23:15 UT).

Reply to Reviewer #2:

Summary of review:

The manuscript presents two case studies of Interplanetary Magnetic Field (IMF) rotations upstream of Mars based on MAVEN and Tianwen-1 magnetometer and plasma measurements. The analysis provides estimates respectively for the response time of the dayside induced magnetosphere (<3.5 min), as well as for the energetic plume of ionospheric O+ scavenged from the ionosphere and picked-up by the solar wind (<3 min).

The study is interesting, the methodology is valid and makes an unusual combined use of multipoint in situ measurements in the Martian system, although it is questionable how big the impact is of a single case study for each phenomenon (magnetosphere and plume), particularly as these mostly appear to confirm previous model results.

Editorially, the writing is mostly concise, though unclear in some places. There are unfortunately too many grammatical errors to be corrected in a scientific review, the manuscript thus requires dedicated proof-reading.

Thanks for the referee's comments. The previous study by Romanelli et al. (2019, doi:10.1029/2019GL084151) determined recovery timescales of the dayside Martian magnetosphere normalized by the IMF variability timescale, mainly based on the time-dependent LATMOS Hybrid Simulation. However, observations of response process of the induced

magnetosphere to external magnetic field are lacking to date. Our work presents the first multi-spacecraft estimation of response time of the Martian magnetosphere to the IMF rotation, based on simultaneous observations by MAVEN and Tianwen-1 orbiters. Our study suggests that the Martian induced magnetosphere is a rapidly dynamic system that is highly sensitive to change of direction of external interplanetary magnetic field. It cautions us that change of interplanetary magnetic field should be considered as one of the general types of space weather on Mars, and it is essential of monitoring and short-term forecasting of interplanetary magnetic field upstream of Mars. These results can serve as a reference for response of induced magnetospheric models of Venus, Titan and other planets without intrinsic magnetic fields. Moreover, it also should be noted that the proposed methods to estimate response timescale applies not only to this case at Mars but also to the ones at other non-magnetized planets.

General comments:

The physics of the magnetosphere induction process is largely glossed over in the introduction and simply said to be "modulated" by an external magnetic field. A physical description would provide the wide readership of this journal with the background they need to understand the results.

Thanks for this suggestion. The physical description of the magnetosphere induction process was added in the introduction in Line 45-49 in the revised manuscript.

The readers of this journal would also greatly benefit from a brief overview of the instruments, their capabilities, measured quantities, and the precision/limitations of the measurements, rather than to have to read through the referenced papers.

The detailed introduction of the instruments has been provided in the section Method-Instruments. We added more details about the precision/limitations of the measurements in the revised manuscript.

Along the same topic as above, changes in the magnetic field generated by spacecraft electrical systems can in some cases appear similar to fast IMF rotations in measurements. Have the variable spacecraft field been subtracted from all of the datasets?

The Tianwen-1/MOMAG team (PI: Wang, Y., team member: Zou, Z. as the co-authors of this manuscript) confirmed that the released MOMAG data are well calibrated. All the interferences from the spacecraft have been removed. If there was any uncertainty, it is at the level of 0.1 nT. Figures B1-B2 below show the data from the inner (darker blue) and outer (lighter blue) sensors during the two episodes investigated in this study. Note that these data are in spacecraft coordinates and their zero-offset are not corrected yet but corrected in the released data. If there was any artificial magnetic field caused by spacecraft electrical system, the data profiles from the inner sensor will be fully aligned with those from the outer sensor. The plots below show that the data profiles from the two sensors are almost exactly overlapped, suggesting all the variations in these data are not from the spacecraft. More details can be found in the Wang et al. (2023; doi:10.26464/epp2023028) and Zou et al. (2023; doi:10.1007/s11431-023-2401-2). In this revision of manuscript, we updated the results of magnetic field using the latest-version data.

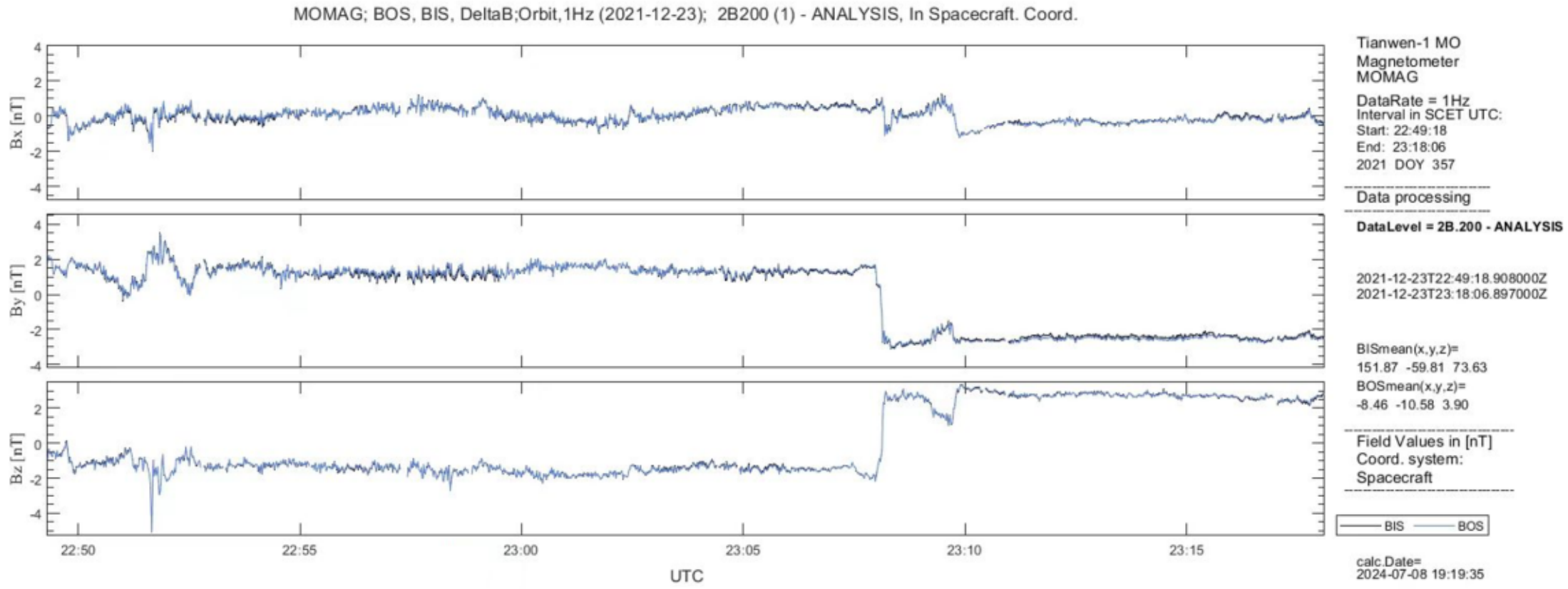

Figure B1. Tianwen-1 MOMAG data from the inner (darker blue) and outer (lighter blue) sensors during 22:50-23:15 UT on December 23, 2021.

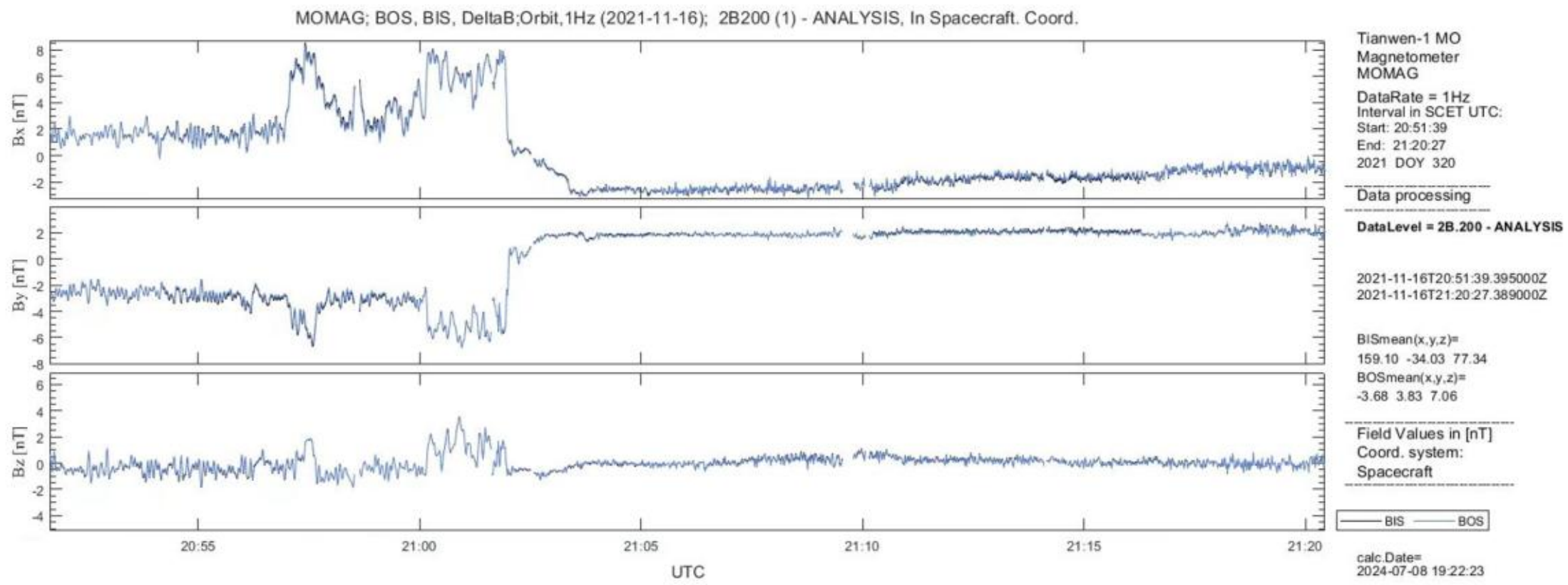

Figure B2. Tianwen-1 MOMAG data from the inner (darker blue) and outer (lighter blue) sensors during 20:55-21:13 UT on November 16, 2021.

In addition, the absolute vector accuracy of magnetic field from MAVEN/MAG is 0.05% (Connerney, Espley, Lawton, et al., 2015, doi:10.1007/s11214-015-0169-4; Connerney, Espley, DiBraccio, et al., 2015, doi:10.1002/2015GL065366).

The introduction has a lengthy description of Titan and Venus, I am not sure why this is relevant, if there is a reason it should be clearly stated for the reader.

Thanks for the referee's comments! Since the evolution of magnetic field is described by the same equation, i.e., magnetic induction equation, the theory of induced magnetosphere in the unmagnetized planets with atmosphere/ionosphere should be the same but it is expected that there exist different features since different planets/stars have different physical parameters and probably encounter external plasma environments. Previous studies showed different response or recovery timescales of Mars, Venus and Titan's induced magnetosphere (e.g., Modolo et al., 2012, doi:10.1029/2011GL049895; Romanelli et al., 2019, doi:10.1029/2019GL084151; Ma et al., 2020, doi:10.1029/2020GL087593; Bertucci et al., 2008, doi:10.1126/science.1159780). However, these studies are mostly based on simulations and relative observations are still lacking. Due to the capability of simultaneous observations in the solar wind and in the induced magnetosphere, it is now available for us to directly investigate the response process of induced magnetosphere and we

wanted to discuss the implications of these results for other planets/stars. In this manuscript, we discussed the response/recovery timescales at different spatial regions of different planets, using magnetic induction equation and combining it with analysis on the results. We added more introductions and discussions of significance of our study to other unmagnetized planets like Venus and Titan in Line 327-341 in the revised manuscript.

The reader is referred to the Supplementary Materials for a description of the empirical magnetic field model used in the analysis. However, the supplementary materials document only contains (besides a title page) a figure with slices through the model and a brief caption that mostly describes the figure, not the model. The reader is left to guess e.g. how this model was created, which "empirical" measurements were used, whether it is based on mean values, medians, or another reduction method, whether crustal fields are excluded, as well as whether upstream conditions have been constrained to be similar to the period of the case study. The description needs to be greatly expanded to be useful to the reader. Also, I cannot find any units for the values shown in the supplementary figure.

Thanks very much for this suggestion. The detailed descriptions about the model were added in the revised supplementary materials document and the units for the values were also added in the Supplementary Figures.

Considering that the IMF rotation appears to be far from an ideal discontinuity in the November 16 case, the manuscript should clarify for the reader how the time of IMF rotation was determined, as this is crucial for determining the response time.

We determined the start time of IMF rotation when the clock angle of IMF started to significantly change (21:01:43 UT). And the end of IMF rotation was when the clock angle of IMF turned to be stable (21:03:23 UT). When the changing IMF reaches the induced magnetosphere boundary, the magnetospheric magnetic field begins to change under the influence by the IMF. Therefore, we took the time when the changing IMF reaches the induced magnetosphere boundary as the beginning of the recovery time.

Line comments:

Line 22: What is a "planet wind"? At first, I thought it could be atmospheric winds, but this is supposed to be something interacting with the atmosphere. A more fitting word should probably be found.

We replaced it with 'planetary plasma flows'.

Line 42: What does "directly" mean in this context? The solar wind interaction is generally not collisional, hence this phrasing could be misleading. A physical description would be much clearer.

We rewrote this sentence and provided a more physical description.

Line 43: in -> of

We corrected it in the revised manuscript.

Line 78: This phrasing is a bit unclear, several spacecrafts have carried magnetometers (Mars

probes, Phobos-2, MGS, etc.), but no two magnetometer equipped orbiters have operated simultaneously.

Thanks for the referee's suggestion. We have replaced it with 'no two magnetometer equipped orbiters have operated simultaneously' in the manuscript.

Lines 81-82: "clearly clarify" should be rephrased. Quantify?

Thanks for the referee's reminding. We replaced "clearly clarify" with "quantify" in the revised manuscript.

Line 83: What is the controversy (disagreement) on this topic? It would be more precise to say that the arrival of Tianwen-1 has enabled such studies.

Thanks for the referee's suggestion. We replaced it with "The arrival of Tianwen-1 orbiter at Mars provides the opportunity to perform such quantitative studies" in the revised manuscript.

Line 125: time-dependent

We corrected it in the revised manuscript.

Lines 167-170: There is no need to assume speeds in the solar wind and sheath. These were measured by MAVEN, the data are publicly available and should be used to avoid needless errors.

Thanks for the referee's suggestion. MAVEN encountered the undisturbed solar wind in the time interval of 21:20-22:20 UT and the Martian magnetosheath in the time interval of 22:50-23:00 UT. Assuming little change in solar wind conditions, the average speeds of protons during these two episodes, which are 339 km/s and 123 km/s, can be regarded as the plasma speed in the solar wind V_{sw} and the magnetosheath V_{sheath}, respectively. The time difference and the recovery time were recalculated with these two speeds in the revised manuscript.

Line 171 and others similarly: "subsolar" is an adjective. Here, perhaps this is referring to the subsolar point?

We replaced "subsolar" with "subsolar point" in the revised manuscript.

Figure 3j: I appreciate the color coding the authors used in this figure, also adding labels with 1 and 2 circled (as above the time-series panels) to the blue and red arrows would also help guide the readers between the panels.

Thanks for the referee's suggestion. The labels are added in the Figure 3j in the revised manuscript.

Reply to Reviewer #3:

Review of Lin et al., 2024 "Direct Observations of Rapid Response of Martian Induced Magnetosphere to Interplanetary Magnetic Field Rotation"

1. Key results

This manuscript presents a novel study that investigates a comparison between MAVEN and Tianwen-1 data. This is a worthwhile pursuit; comparisons studies at Mars are rare even though multiple spacecraft obtaining plasma and magnetic field data are present. The manuscript aims to combine these two datasets to estimate various global responses (planetary ion plume, magnetic field draping) from MAVEN to an upstream IMF rotation as measured by Tianwen-1. A main argument of this paper is the relevance of these results to both Venus, and Titan studies. It is argued that these findings are to some extent, universal in induced magnetospheres. This reviewer understands the challenges inherent in cross-mission studies and finds that this undertaking provides a useful comparison for the field. As written however, the manuscript could be improved in: validity, approach, and clarity to enhance the accuracy, impact, and accessibility of these findings to the wider Mars and induced magnetospheres communities.

Thanks for the referee's suggestions and comments. We added more descriptions on the Validity of the methodology, the applicability of the method and significance of the results to the wider Mars and induced magnetospheres communities in the revised manuscript. The relevance of these results to Venus and Titan studies is also illustrated in the detailed response below and updated in the revised manuscript.

2. Validity

In general, approaching estimations of IMF response with multiple spacecraft is appropriate. However, there are limitations in the approach that raise significant doubts as to the interpretation of the findings. For example:

• ***Crustal Field and MSE:*** *At Mars it is well known that the crustal fields can influence observed magnetic fields at high (e.g. ~1000 km) altitudes and in particular will alter observations over the southern hemisphere as compared to the northern hemisphere. In addition, much has been written about the global draping morphology as unexpected, or hemi-spherically dichotomous due to either polar differences or hemi-spherically dependent draping fields in the magnetosheath. The approach of a 'mean background field' is a good idea, however using MSE obfuscates hemispheric differences including crustal fields and draping differences; IMF asymmetries, and low altitudes over the southern hemisphere are always circumspect to the crustal fields.*

- *It is also worth noting, that basing analyses' in MSE makes assumptions about the upstream IMF. It is unstated if these are mitigated in the author's approach.*
- *Taken in aggregate, it is unclear if the results presented here are truly dependent on the upstream IMF, or a result of crustal fields or other MSE effects.*

• ***Assumptions of Draping:*** *Throughout the manuscript it is assumed that the IMF upstream maps directly to the draped orientation. It is known that this is a limited assumption, especially under radial (or significant IMF Bx) conditions and this manuscript could benefit from additional discussion of how this limitation is addressed.*

- *For example, the manuscript focuses on IMF along X but uses the clock angle in Y and Z, there is an unstated assumption as to this interpretation.*

Relevant (non-exhaustive) references for the above items:

§ Chai et al., 2019 https://iopscience.iop.org/article/10.3847/2041-8213/aaff6e/meta and relevant citations within incl. Dubinin about polar field effects from (assumably) plume dynamics

§ Zhang et al., 2022
https://agupubs.onlinelibrary.wiley.com/doi/abs/10.1029/2022JE007334, a more complete MSE based understanding of global magnetic fields
§ Azari et al., 2023
https://agupubs.onlinelibrary.wiley.com/doi/full/10.1029/2023JA031546, quantification of crustal fields effects at high altitudes, demonstration of non-direct draping
§ Liemohn et al. 2017
https://agupubs.onlinelibrary.wiley.com/doi/full/10.1002/2016JA023707 discussion of the effective limitations of both MSO and MSE
§ Langlais et al., 2019
https://agupubs.onlinelibrary.wiley.com/doi/full/10.1029/2018JE005854, crustal field model (one of a few including several previously)
§ Brain+2006
https://www.sciencedirect.com/science/article/abs/pii/S0019103505004902?via%3Dihub, demonstration of potential IMF based draping asymmetries
§ Fowler+2022,
https://agupubs.onlinelibrary.wiley.com/doi/abs/10.1029/2022JA030726 discussion of radial IMF

Based on the referee's comments, we further analyzed whether the event on December 23, 2021 were influenced by the crustal field and MSE effects. And the relevant references are cited in the revised manuscript.

We checked the geographic coordinates corresponding to the event and found that this event occurred over the southern hemisphere but with a weak crustal field (as shown in the Figure C1). Besides, the magnetic fields observed by the MAVEN orbiter were quite disturbed and the magnitude was less than 30 nT, which is typical of non-crustal fields (23:00-23:15 UT in Figure 1c). These suggest that the rotation of magnetic field observed by MAVEN is not from crustal field effect.

We checked the spatial location of the event and found that this event was in the +E hemisphere both before and after the IMF rotation. Draping asymmetries are usually considered in the -E hemisphere (Zhang et al., 2022); whereas in the +E hemisphere, the ideal draped model is acceptable (Zhang et al., 2022). In order to remove the influence of draping asymmetries or other MSE effects, we introduce a mean background field model. This average background field model represents the steady-state magnetic field at different locations in the Martian magnetosphere under a stable IMF condition, which incorporates draping asymmetries and other MSE effects. By subtracting the observed magnetic field from the magnetic field of the mean background model, the difference between the magnetic fields in the time-varying case and those from the steady-state model can be obtained; theoretically, as long as the mean background field model is sufficiently accurate, this difference does not contain draping asymmetries and other MSE effects but only time-varying information about the magnetic field. Thus, we can analyze the response of the Martian magnetosphere to IMF rotation by analyzing the variation of this difference.

Using single-satellite observations, the analysis in the MSE coordinate system requires assumptions about the IMF, because it is not possible to observe the IMF and the magnetospheric magnetic field

at the same time. However, multi-satellite observations can enable such simultaneous observations, and therefore no assumptions about the IMF are required. In this paper, multi-satellite observations are utilized, and no assumptions about the IMF are required.

The cases of radial IMF are very interesting and, as the reviewer commented, should be considered. However, we surveyed the MAVEN data about the cone angle of IMF at Mars. It showed that the cases of radial IMF are much less than those of large cone angle of IMF. Also, we surveyed data from Tianwen-1 and we have not yet found an event of radial IMF in the response process of Martian induced magnetosphere. Thus, we would like to continue this research once we find the relative observations in the future. In the revised manuscript, we further clarified this assumption in Line 277-279.

As shown in Figs. 1b and 1c, the main varying components of the IMF are B_x and B_z, while both the B_x and B_z components of the magnetospheric magnetic field have corresponding variations, with the B_x variation being more significant. It should be noted that there exist the interconnected changes in the B_z and B_y components as the satellite traverses different positions in the magnetosphere. The changes in both ultimately reflect the changes in the clock angle. And the variation of the B_x component depends mainly on the changing characteristics of the IMF. Therefore, in the MSO coordinate system, if we want to analyze the correlation between different magnetic field components in terms of waveform characteristics, the B_x component is a more suitable candidate.

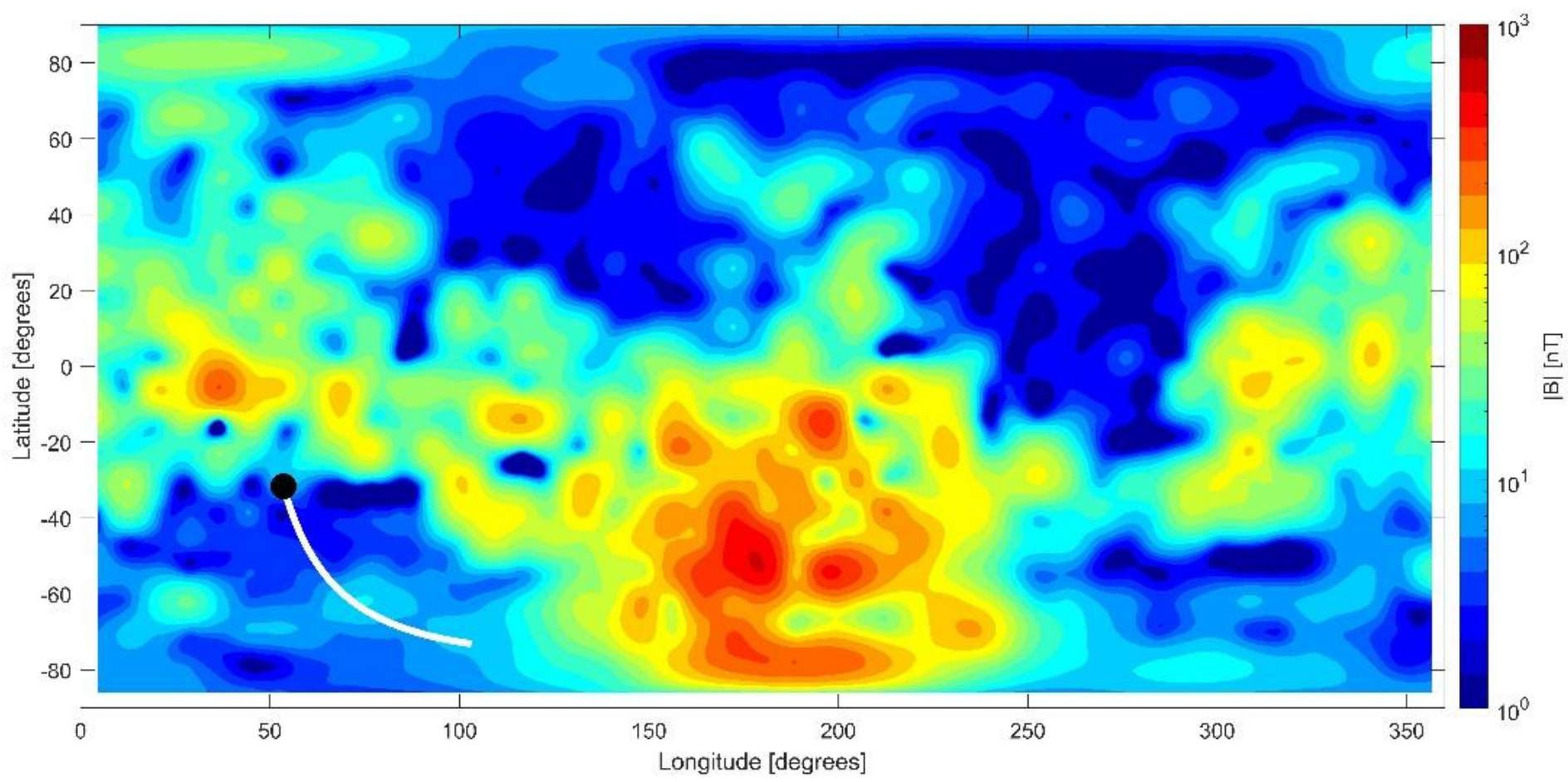

Figure C1. The orbit of MAVEN in the geographic coordinate and the crustal magnetic field magnitude. The contour map represents the magnitude of crustal magnetic field at altitude of 185 km by model (Morschhauser et al. (2014), doi:10.1002/2013JE004555). The black dot represents the location of MAVEN at 23:00 UT and the white line represents the orbit in the time interval of 23:00-23:15 UT on December 23, 2021.

3. Significance

➢ *This study provides (to the reviewer's understanding) the first multi-spacecraft estimation of response rate of the IMF at Mars. However previous studies*

https://agupubs.onlinelibrary.wiley.com/doi/full/10.1029/2019GL084151 have provided such estimations and these are only superficially compared to the results.

- *In addition, there are severe limitations for comparison to other planets (e.g. Venus, Titan) given the significantly different atmospheric constituents (and ionospheric compositions), densities, and upstream conditions (e.g. Titan is in Saturn's magnetosphere). It is unclear how the author's considered such limitations and casts doubt as to the claim regarding unified theories of induced magnetospheric response rates to upstream conditions.*

The previous study by Romanelli et al. (2019, doi:10.1029/2019GL084151) determined recovery timescales of the dayside Martian magnetosphere normalized by the IMF variability timescale, mainly based on the time-dependent LATMOS Hybrid Simulation. However, observations of response process of the induced magnetosphere to external magnetic field are lacking to date. Our work presents the first multi-spacecraft estimation of response time of the Martian magnetosphere to the IMF rotation, based on simultaneous *in-situ* observations by MAVEN and Tianwen-1 orbiters.

Like Mars, Venus and Titan have atmospheres but no global intrinsic magnetic field, and both can form induced magnetospheres in interaction with external magnetic fields and plasma flows. Despite the fact that Venus, Titan, and Mars possess different atmosphere, the physical process that non-magnetized atmospheres interact with external magnetic field should be the same. Based on the results of this paper, we verify that in the region above the altitude of the magnetic frozen boundary, the recovery time of the induced magnetosphere depends on the thickness of the induced magnetosphere and the plasma bulk velocity within the induced magnetosphere. This leads to the assumption that the recovery time of the magnetic field within the induced magnetosphere of Venus and Titan should also depend on the thickness of the induced magnetosphere and the plasma bulk velocity. The difference is that the atmospheres of Venus and Titan are denser and have different parameters for both the external magnetic field and the upstream plasma flow. If the thickness of the induced magnetosphere and the corresponding plasma flow velocity can be determined, the recovery time of the induced magnetosphere for Titan and Venus can be estimated by the same method. Once the magnetic field reaches below the magnetic freezing boundary by convection, the magnetic diffusion term plays a major role and dissipates the magnetic field in the form of ohmic dissipation.

We added these discussions of comparison to other planets in Line 327-341 in the revised manuscript.

4. Data and methodology

The data used is appropriate. Please note that http://202.106.152.98:8081/marsdata/ is unreachable at the time of the reviewer's report.

Thanks for the referee's reminding. The data source was updated in the revised manuscript.

5. Analytical approach

There are several minor issues regarding the approach that could be addressed with additional text.

- *It is concerning that the correlation coefficient peaks in the positive time scales. More details would be useful in deciphering this.*
- *There are a few different types of correlation coefficients, it would be useful to add context to*

this discussion to understand the assumptions being made.

- *More details on the empirical model would assist in interpretation, for example this is a gridded product in the associated supplementary, but is used for continuous estimation in the plots.*

Detailed descriptions of the linear correlation method are provided in section Method in the revised manuscript.

The local minimum in Figure 2c suggests that there may be a correlation between these two data in terms of waveform characteristics but it requires one of the data series to be inverted. However, the corresponding positive time delay ($l > 0$) means that the magnetospheric data (measurements from MAVEN) needs to be shifted forward on the time axis. Physically, any correlation coefficient with a positive time lag is meaningless, since it is clear that the magnetospheric magnetic field is influenced by the IMF and not the other way around. Thus, we decided to delete the results with positive time lag in the revised manuscript in order not to confuse readers.

In the revised manuscript, we added more descriptions about linear correlation method in the section Method and descriptions about the empirical model of magnetic field in the Supplementary Information. The limitations on the method were added in the section Method in the revised manuscript.

6. Suggested improvements

- *Most of the comments above (and below, see clarity and context) can be addressed with additional details. There are several issues however that will require additional pursuit including potentially: assessing the impact of crustal field in these analyses and/or using non MSE coordinate systems for these analyses.*
- *Additional grammar edits (see clarity and context).*

Thanks for the referee's comment and suggestions. The impact of crustal field in the analyses is assessed and discussed in the response of section "2. Validity".

In the original manuscript we used non MSE coordinate systems for analyses as well. We compared the clock angle of magnetic field observed by MAVEN and Tianwen-1 in the MSO coordinate (blue and red curves in Figure 2d; red curve in Figure 2e). One can see that before the IMF rotation (23:02-23:08 UT), there was little difference between these two magnetic fields; Since the IMF rotation (23:08-23:15 UT), the difference went up to 80 degrees suddenly and decreased gradually to nearly 10 degrees. The recovery time in this process is less than 3.5 minutes. It suggests the rapid response of Martian induced magnetosphere to the IMF rotation. It is clear that these analyses are based on MSO coordinate system and non MSE coordinate systems are involved.

7. Clarity and context

The motivation behind this work is unclear with respect to the connection between Titan, Venus, and Mars. For example, it is expected that these should be significant differences within the theory of induced magnetospheres but such differences are not discussed.

Thanks for the referee's suggestion! Previous studies showed different response or recovery timescales of Mars, Venus and Titan's induced magnetosphere. Since the evolution of magnetic field is described by the same equation, i.e., magnetic induction equation, the theory of induced

magnetosphere in the unmagnetized planets with atmosphere/ionosphere should be the same but it is expected that there exist different features since different planets/stars have different physical parameters and probably encounter external plasma environments. Due to the capability of simultaneous observations in the solar wind and in the induced magnetosphere, it is now available for us to directly investigate the response process of induced magnetosphere and we wanted to discuss the implications of these results for other planets/stars. In this manuscript, we discussed the response/recovery timescales at different spatial regions of different planets, using magnetic induction equation and combining it with analysis on the results. We added more introductions and discussions of significance of our study to other unmagnetized planets like Venus and Titan in the revised manuscript.

8. References

A discussion of induced magnetospheres is warranted in this work but not provided. Several substantial papers regarding fundamental review of induced magnetospheres in space physics are not included such as:

- *Luhmann+2004, https://www.sciencedirect.com/science/article/abs/pii/S0273117704000158*

Thanks for the referee's suggestion! We added introductions of induced magnetosphere and associated discussions in the revised manuscript and more papers, such as Luhmann et al. (2004) and Luhmann & Cravens (1991, doi:10.1007/978-94-011-3300-5_4), about induced magnetospheres are also cited.

Reply to the Reviewers

We greatly thank the reviewers for the valuable comments/suggestions that we considered in the resubmitted manuscript. All revised parts are marked in red in the text. Detailed answers to the comments are given below:

--

Reply to Reviewer #1:

I thank the authors for addressing my comments. Although the manuscript is definitely interesting, I find the employed methodology is still not sufficiently justified. Please find below my additional comments.

‘ Line 29: I suggest adding a bit more information about the IMF rotation. Around what axis does the IMF rotation take place? Does it imply changes in the IMF cone angle, clock angle or both? The IMF rotation took place on the X axis and it means the change of IMF clock angle. We referred it as ‘the IMF clock angle changed’ in the revised manuscript.’
Thank you for the added information. However, I believe this sentence is incorrect. The IMF primarily changed in the Bx and Bz components (see Figure 1b). Therefore, the main rotation seems to occur around the Y-axis, which is associated with changes in both the IMF cone and clock angles.
Thanks for the referee’s comment and reminding. Figure A1 shows the cone and clock angles of interplanetary magnetic field (IMF) in this event. The IMF cone angle changed from ~51 degrees and became stable at ~67 degrees. And the IMF clock angle changed from ~291 degrees to ~213 degrees. It is clear that both the IMF cone angle and clock angles changed. We added description about the change of IMF cone angle in the revised manuscript.

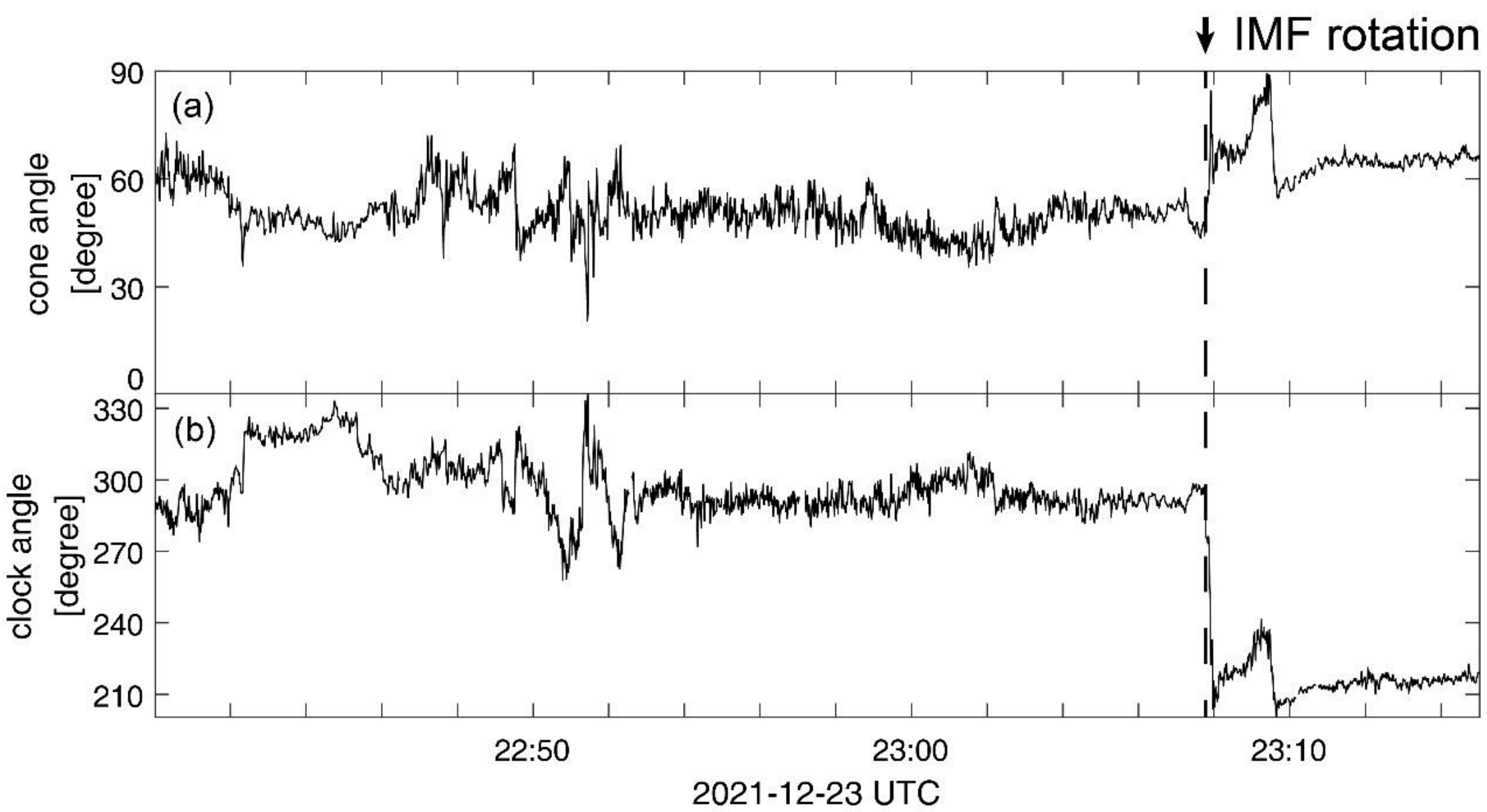

Figure A1. Cone angle and clock angle of interplanetary magnetic field measured by Tianwen-1 in the time interval between 22:50-23:15 UT, on December 23, 2021.

'...And the variation of the Bx component depends mainly on the changing characteristics of the IMF.'

I do not agree with this statement. Bx, just like By and Bz display variability as MAVEN traverses different regions of the Martian magnetosphere. I still think the manuscript does not provide a justification for the employed methodology. Thus, I find the interpretation and conclusions questionable.

Thanks for the referee's comment.

According to the draping field scenario, the direction of magnetic field in the induced magnetosphere is mostly parallel to the surface (Figure A5a). As it goes to the terminator or magnetotail region, the B_x component of magnetic field dominants. This causes the interconnected changes in the B_y and B_z components as the orbiter traverses different positions in the magnetosphere. The changes in both ultimately reflect the changes in the clock angle.

This methodology is employed in order to confirm whether there exists correlation between the magnetic field in the induced magnetosphere and the IMF and to roughly estimate the response/recovery time, if these two signals are correlated. We chose Bx component of magnetic field to do the calculation because of the B_x components of these two signals both showed the most distinct feature of variation.

However, it did not provide with other information as the second methodology did. And as the reviewer pointed out, it is difficult to quantify the variation between the three components of magnetic field. Since by employing the second methodology, we had already obtained enough information to draw our conclusions, we decided to remove the first methodology from the manuscript.

'Lines 146-149 and Figure 2: The MSE average employed to compute the steady-state empirical model of magnetic field does not appear to consider other factors, such as variability due crustal field rotation. What about the effects due to local time? Should not be these considered when analyzing two case studies? What is the average Bx of the IMF for all cases used in this averaged picture? Is it consistent with the Bx component shown in Figure 1?

Magnetic field measurements above the crustal fields are mostly removed from the dataset of the empirical magnetic field model. About the first case on December 23, 2021, we checked the geographic coordinates corresponding to the event and found that this event occurred over the southern hemisphere but with a very weak crustal field (as shown in the Figure A4). As for the other case on November 16, 2021, the orbiters are at much higher altitude than the areas that are influenced by crustal fields. Thus, the crustal field effect does not affect either the empirical magnetic field model or the results. There are separate datasets for each local time and each altitude. The average of magnetic field in each dataset makes up the model. Therefore, the empirical magnetic field model contains local time effect. We used the average cone angle to represent the average Bx of the IMF. Measurements of the small cone angle (less than 30 degrees) of IMF degrees are removed from the dataset. In the first case shown in the Figure 1, the cone angles of IMF are mostly larger than 45 degrees and the average cone angle is about 54.'

In my opinion, the use of the MSE average map is not sufficiently justified. Firstly, crustal magnetic

fields can globally influence the Martian magnetosphere (e.g., Dubinin et al. 2023, DiBraccio et al. 2022). Additionally, there is no clear justification for excluding observations with IMF cone angles smaller than 30 degrees when creating the MSE map. While I understand that foreshock processes might impact the results, this exclusion seems arbitrary.

Thanks for the referee's comment.

As for the influence of crustal field on the Martian magnetosphere, we provide more descriptions and references to justify the method.

Firstly, the Martian magnetosphere can be roughly summarized as an induced magnetosphere (Venus-type) with crustal field or dipole-field at some fixed location (e.g., Acuña et al., 1998, doi: 10.1126/science.284.5415.790; Dubinin et al., 2023, doi:10.1029/2022JA030575). Recent studies investigated the "mini-magnetosphere" that formed in the interaction between the crustal field and solar wind/IMF (Fan et al., 2023, doi:10.1029/2023GL103999). However, it should be noted that the mini-magnetosphere is restricted at locations of crustal fields and at the locations of no/weak crustal fields the Martian magnetosphere should be regarded as the induced magnetosphere (Venus-type). Secondly, according to Dubinin et al. (2023), the crustal field is reconnected to the IMF and thus affects the original draping field, which means that the effect of the crustal field on the Martian magnetosphere is limited to regions close to the crustal field and, as an important prerequisite, it must be reconnected to the IMF. This suggests that the effect of the crustal field is not dominant and can be ignored when it is geographically far from the crustal field. There already existed numerous case studies and statistical studies that evaluated the effect of crustal field by identifying whether the event is occurred close to the geographical region of crustal field (e.g., Zhang et al., 2024, doi:10.1038/s41586-024-07959-z; Dubinin et al., 2019, doi:10.1029/2019gl084387; Dubinin et al., 2021, doi:10.1029/2021ja029542; Chai et al., 2019, doi: 10.3847/2041-8213/aaff6e). It suggests that the influence of crustal field can be ignored and the Martian magnetosphere should be regarded as the induced magnetosphere (Venus-type) at the locations of no/weak crustal fields.

To a first approximation, Mars' interaction with the solar wind is similar to that of an unmagnetized body where the solar wind magnetic field drapes around the planet and geometries of draping on the dayside propagate downtail (Crider et al., 2004, doi:10.1023/B:SPAC.0000032714.66124.4e; Luhmann, 1992, doi:10.1016/0273-1177(92)90331-Q; Luhmann & Brace, 1991, doi:10.1029/91RG00066; Luhmann & Cravens, 1991, doi:10.1007/BF00177138). It suggests that the global effect of the crustal field on the Martian magnetosphere is much less important than draping field or induced field. The solar wind interaction with Mars is primarily ionospheric-atmospheric interaction similar to that at Venus (Cloutier et al., 1999, doi:10.1029/1999GL900591), where there exists no crustal field or dipole field.

As for the reported event in this study, the region of the event is clearly far away from the crustal field region (Figure A2), which suggests that analysis associated to this event should not focus on the effect of the crustal field. Besides, the magnetic field measurements are in quite good agreement with the empirical model that excludes the global effect of the crustal field, both before and after the IMF deflection (blue and green lines in Fig. 2d in the main text). And the empirical model

represents the induced magnetosphere without crustal fields. In order to investigate whether the effect of crustal magnetic fields has been removed from this model. A second empirical model is obtained in which the magnetic field measurements are removed where the crustal magnetic field magnitude by model (Morschhauser et al., 2014, doi:10.1002/2013JE004555) at altitude of 185 km (Figure A2) is larger than 50 nT. The results are shown in Figure A3 and A4. Note that clock angles of magnetic field measurements in MSE coordinates by virtual satellite in this steady-state empirical model (Figure A4c) are almost the same as those in the first empirical model (Figure 2c in the revised manuscript). It suggests that the effect of crustal fields has been removed from the first model and this model can represent steady-state magnetic field in the Mars' induced magnetosphere. All these evidences suggest that the draping field plays a dominant role and the effect of crustal field can be ignored in our event.

Last but not least, the subject of this study is the response of the Martian "induced magnetosphere" to the interplanetary magnetic field. This study focuses on the induced magnetosphere and not on the crustal field or mini-magnetosphere. Therefore, the influence of the crustal field is a factor to be avoided as much as possible in this study. The MSE coordinate, which excludes the effect of crustal fields to some extent, is a very suitable coordinate for analyzing the induced magnetosphere. This has been demonstrated in many past studies (e.g., Dubinin et al., 2023, doi:10.1029/2022JA030575). And the empirical model excludes the effect of crustal field by excluding dataset that near the crustal field.

We agree with the reviewer that the effect of crustal fields on the Martian magnetosphere is indeed a very interesting topic, and the answer remains open to the community. However, the subject of this study does not involve crustal fields, and that this study circumvented the effect of crustal field on the Martian magnetosphere by selecting events that do not involve crustal field, and by excluding the effect of crustal field when creating empirical models. It actually helps excluding other factors but the main object of our study, the Martian induced magnetosphere.

We added more descriptions in the capabilities and limitations of the employed methods in the Method section to justify for excluding the effect of crustal field when creating the MSE map.

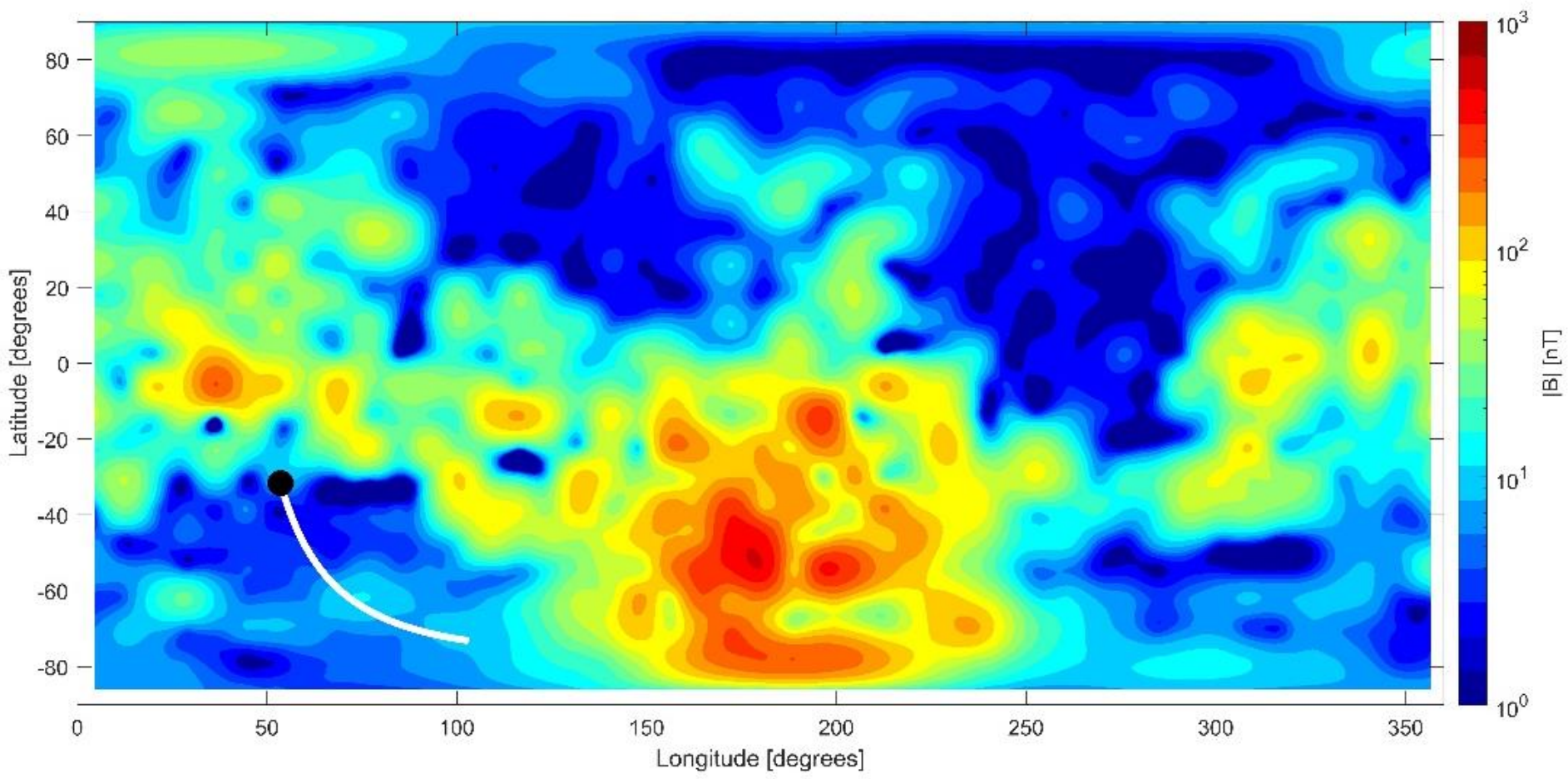

Figure A2. The orbit of MAVEN in the geographic coordinate and the crustal magnetic field magnitude. The contour map represents the magnitude of crustal magnetic field at altitude of 185 km by model (Morschhauser et al., 2014, doi:10.1002/2013JE004555). The black dot represents the location of MAVEN at 23:00 UT and the white line represents the orbit in the time interval of 23:00-23:15 UT on December 23, 2021.

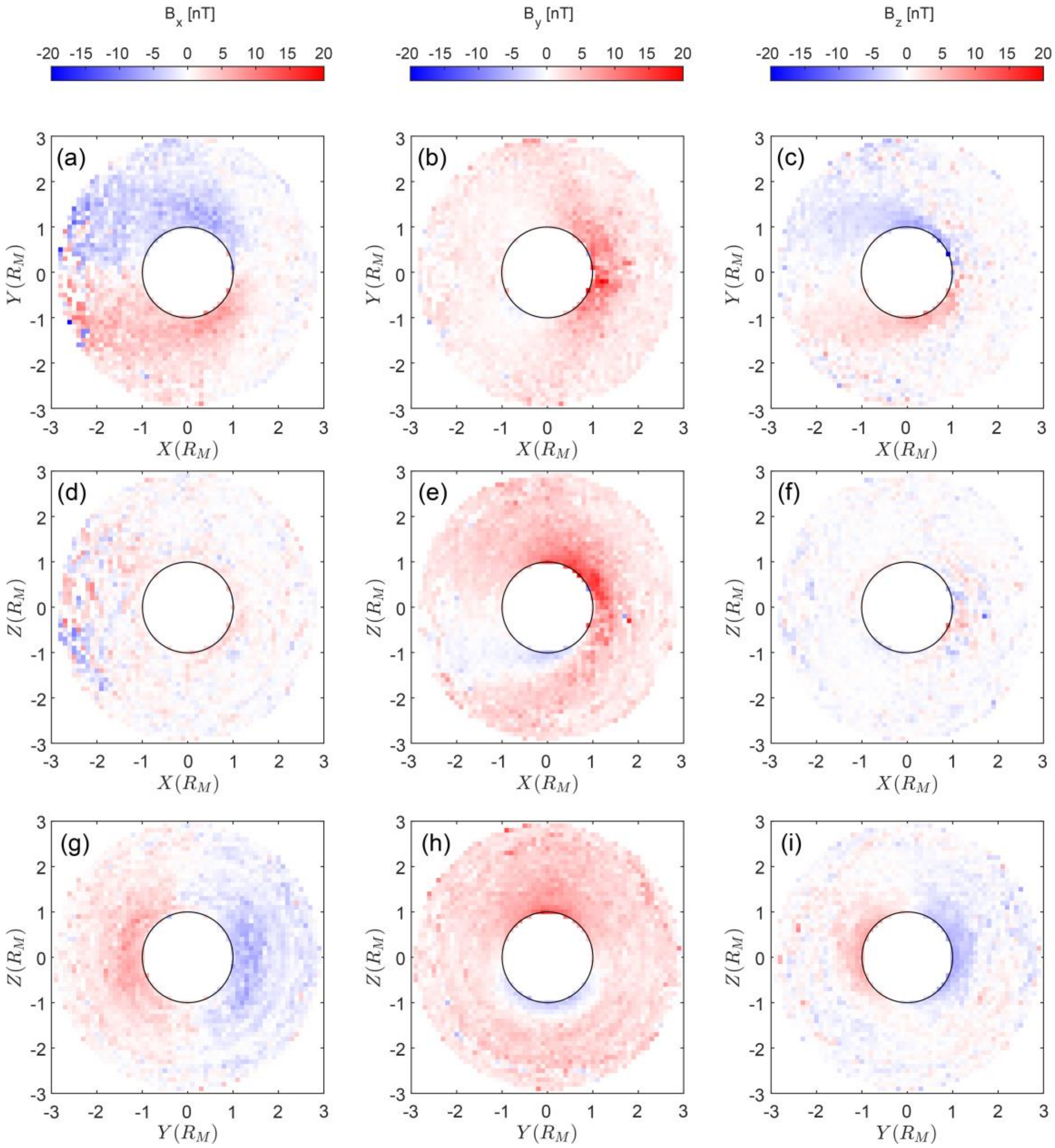

Figure A3. Slices through the steady-state empirical model of magnetic field in the MSE coordinates at Mars. Three components of magnetic field are shown in equatorial plane (a–c), in the day–night plane (d–f), and in the dawn–dusk plane (g–i). The grid spacing is 0.10 R_M. All slices are 0.10 R_M thick. Each column of panels is colored by the components of the magnetic field in MSE coordinates, respectively. The thin black circle in each panel represents the Mars. The magnetic field measurements were removed from the dataset where the crustal magnetic field magnitude by model[2] at altitude of 185 km is larger than 50 nT.

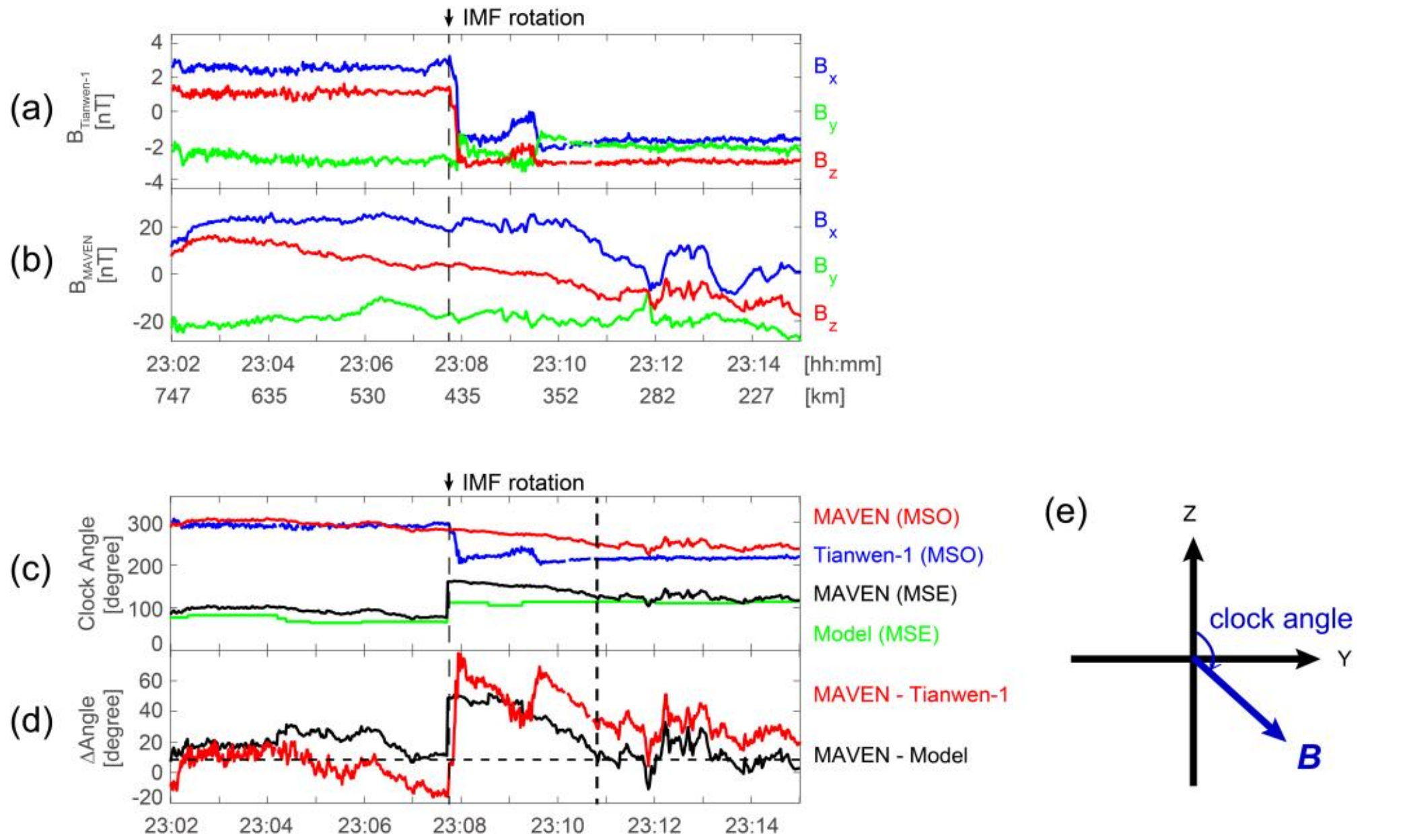

Figure A4. Magnetic field and plasmas measurements by MAVEN in the magnetosphere, accompanying by the interplanetary magnetic field measurements by Tianwen-1 in the solar wind. (a-b) Magnetic field in MSO coordinates by Tianwen-1 and by MAVEN, (c) clock angle of magnetic field measurements and steady-state empirical model, (d) difference of clock angles in MSO and MSE coordinates, and (e) cartoon of description of clock angle in y-z plane in MSO coordinates. In panel (c), red and blue lines represent magnetic field measurements in MSO coordinates by MAVEN and by Tianwen-1, whereas black and green lines represent magnetic field measurements in MSE coordinates by MAVEN and by virtual satellite with the same orbit of MAVEN in the steady-state empirical model. The MSE coordinates are obtained respectively by the averaged clock angle of IMF in the time interval between 23:02-23:07 UT and 23:11-23:15 UT. The former clock angle is 294 degrees and the latter one is 211 degrees. In panel (d), red line represents the difference of clock angles between MAVEN's and Tianwen-1's in the MSO coordinates and black line represents the difference of clock angles between MAVEN's and virtual-satellite's in the MSE coordinates. Cartoon at the bottom right corner of the figure shows the definition of clock angle. The magnetic field measurements in the model were removed from the dataset where the crustal magnetic field magnitude by model at altitude of 185 km is larger than 50 nT.

As for excluding observations with IMF cone angles smaller than 30 degrees when creating the MSE map, we provide more descriptions and evidence to justify the reasons.

The effect of foreshock processes is one of the reasons, and there are other more important reasons.

There are two typical models of magnetic field in the induced magnetosphere. One is dominant by draping field when the cone angle of IMF is large (Figure A5a) and the other is dominant by induced magnetic field when the cone angle of IMF is small (Figure A5b). One can see that it is totally different between these two models and the associated IMF conditions are different. As for the case in our study, the averaged IMF cone angle is 54 degrees, which indicates the draping field is dominant. And it refers to the model shown by Figure A5a. Therefore, cases of small cone angle should be excluded from the statistical MSE map for that these cases are not consistent with the

case in our study.

The other concern is why we chose 30 degrees as the lower limit of IMF cone angle? Theoretically, the IMF cone angle in the dataset should be close to 54 degrees as much as possible. However, it is impossible since there are no sufficient data available if we set up a narrow range of IMF cone angle, for example, from 50 to 60 degrees.

To compromise between these two concerns, we set up a lower limit of IMF cone angle to exclude the 'radial IMF' cases. The lower limit is 30 degrees we chose in this study. In practice, there is no much difference of the lower limit of IMF cone angle to be 20 degrees (Figure A6), 30 degrees (Supplementary Figure 2), or 40 degrees (Figure A8). The statistical results are mostly the same since the number of cases of small IMF cone angle is much smaller than that of large IMF cone angle. Figure A7 shows the clock angles in MSO and MSE coordinate with lower limit of IMF cone angle at 20 degrees and Figure A9 shows those at 40 degrees. Recovery timescales are estimated to be approximately 185 seconds (Figure A7b & Figure A9b) which is consistent with that in the manuscript.

As a result, magnetic field measurements are in quite good agreement with the empirical model that excludes the effect of the crustal field, both before and after the IMF deflection (blue and green lines in Fig. 2d in the manuscript). It suggests that the empirical model descripts the steady-state induced magnetosphere well.

We added more descriptions in the Supplementary Information to justify for excluding observations with IMF cone angles smaller than 30 degrees when creating the MSE map.

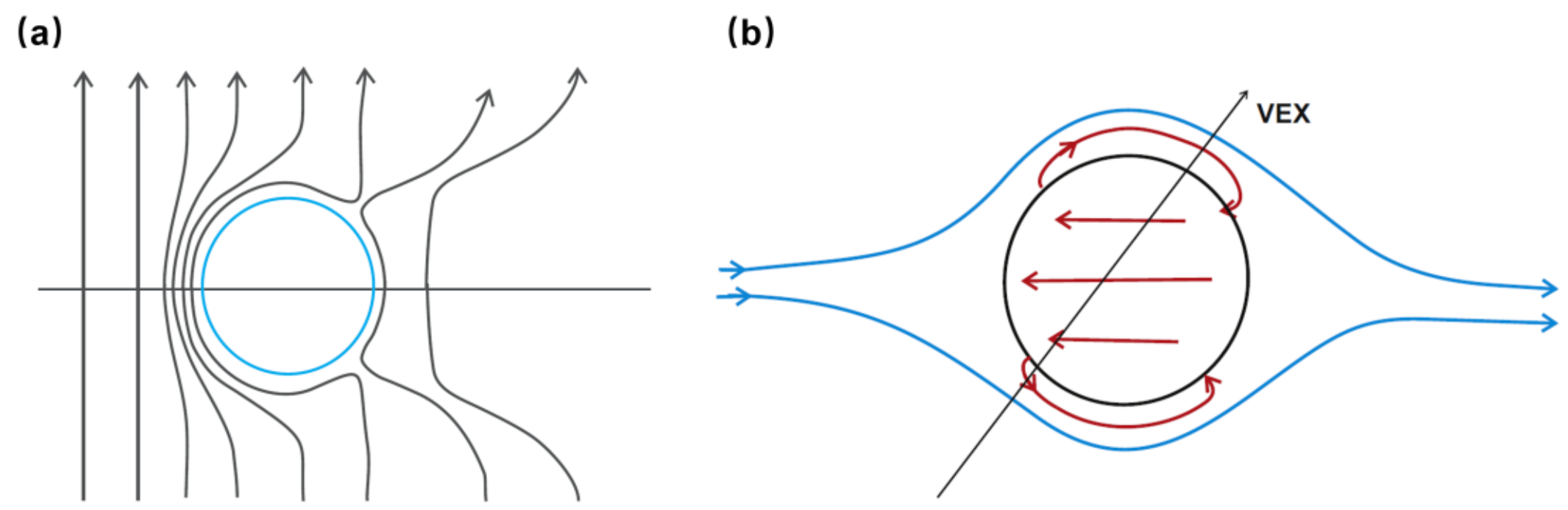

Figure A5. Sketch of possible field configurations (a) associated with 90 degrees of IMF cone angle, and (b) under radial IMF (adapted from Dubinin et al., 2013, doi:10.1016/j.pss.2012.12.003).

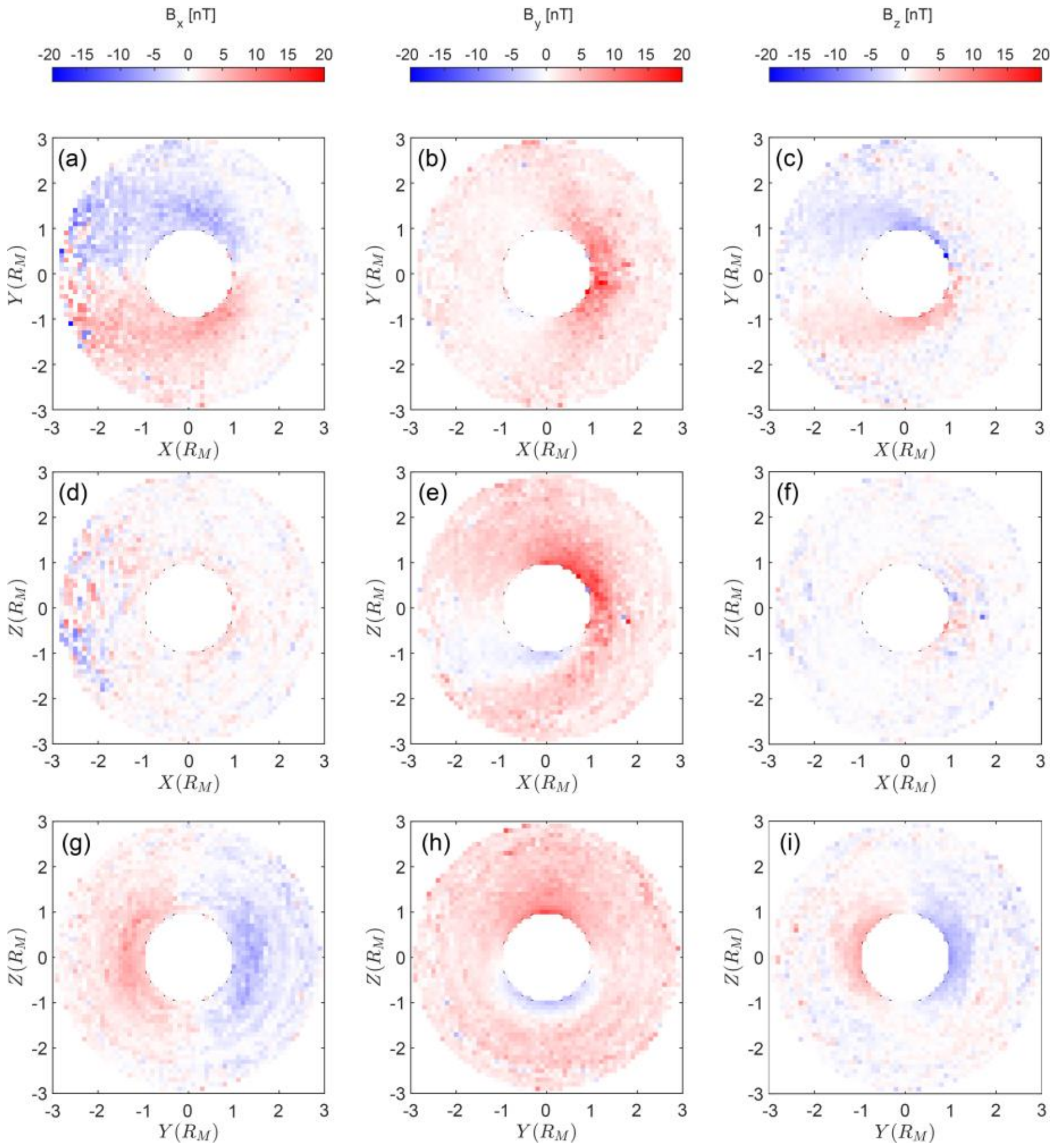

Figure A6. Slices through the steady-state empirical model of magnetic field in the MSE coordinates at Mars. Three components of magnetic field are shown in equatorial plane (a–c), in the day–night plane (d–f), and in the dawn–dusk plane (g–i). The grid spacing is 0.10 R_M. All slices are 0.10 R_M thick. Each column of panels is colored by the components of the magnetic field in MSE coordinates, respectively. The thin black circle in each panel represents the Mars. The lower limit of IMF cone angle is 20 degrees.

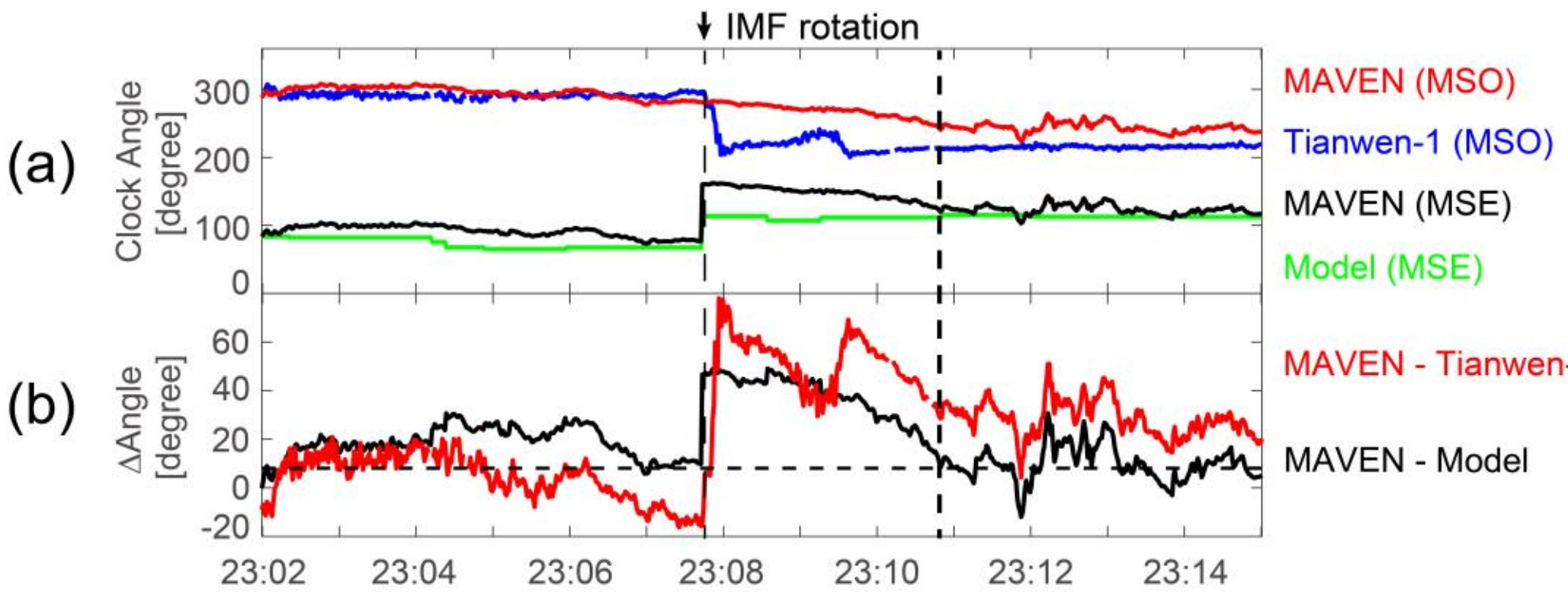

Figure A7. (a) Clock angle of magnetic field measurements and steady-state empirical model and (b) difference of clock angles in MSO and MSE coordinates. In panel (a), red and blue lines represent magnetic field measurements in MSO coordinates by MAVEN and by Tianwen-1, whereas

black and green lines represent magnetic field measurements in MSE coordinates by MAVEN and by virtual satellite with the same orbit of MAVEN in the steady-state empirical model. The MSE coordinates are obtained respectively by the averaged clock angle of IMF in the time interval between 23:02-23:07 UT and 23:11-23:15 UT. In panel (b), red line represents the difference of clock angles between MAVEN's and Tianwen-1's in the MSO coordinates and black line represents the difference of clock angles between MAVEN's and virtual-satellite's in the MSE coordinates. The lower limit of IMF cone angle when creating the MSE model is 20 degrees.

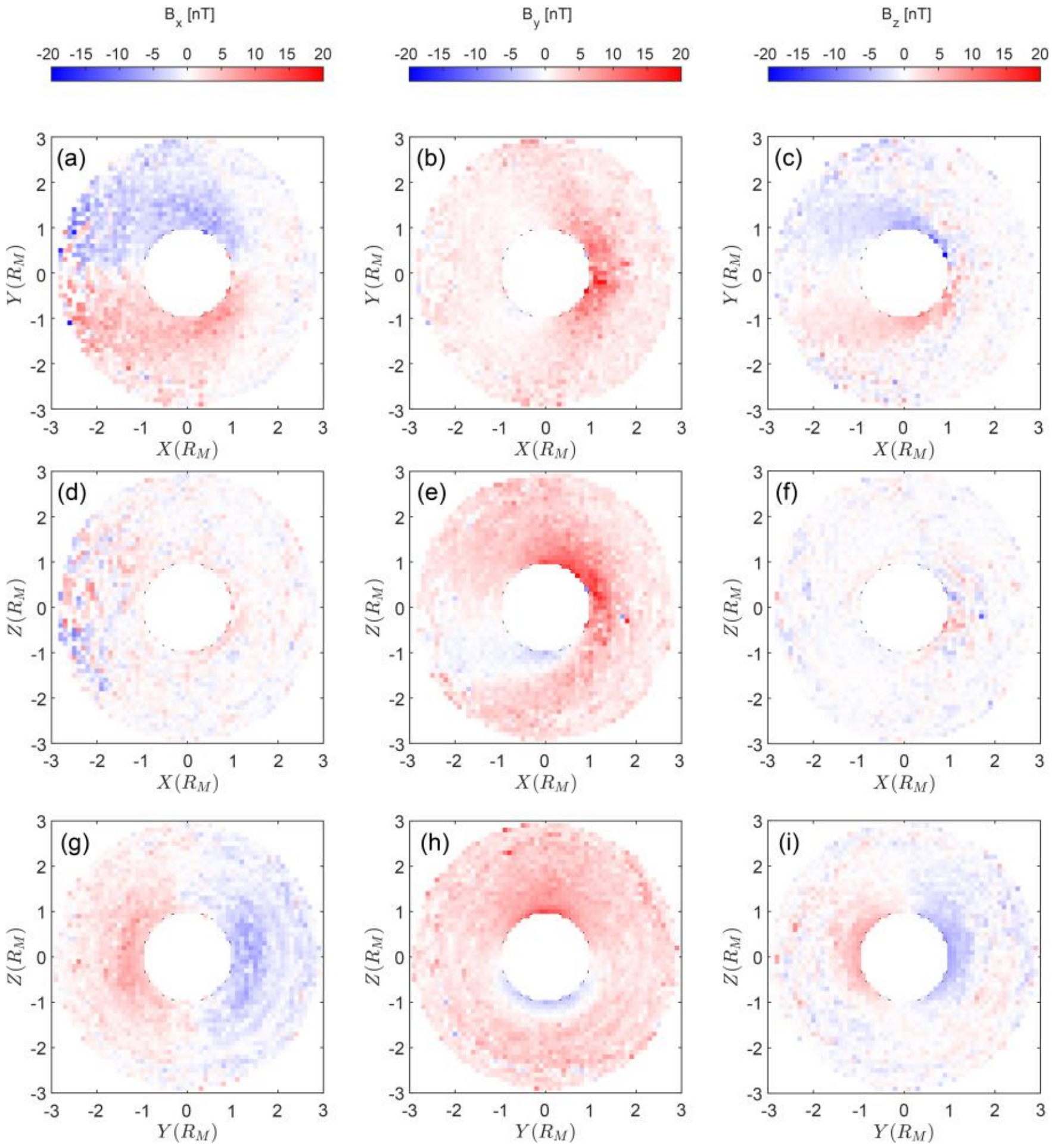

Figure A8. Same format as Figure A6 but lower limit of IMF cone angle is 40 degrees.

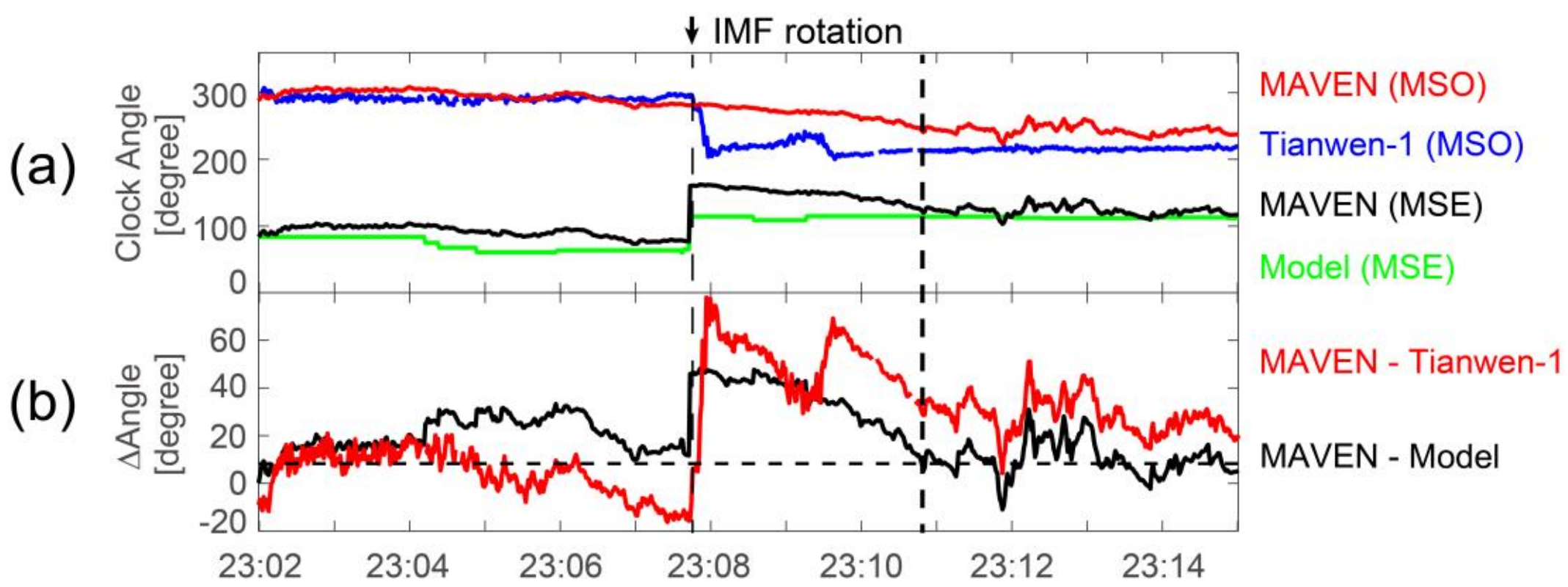

Figure A9. Same format as Figure A7 but lower limit of IMF cone angle is 40 degrees.

Furthermore, the manuscript lacks a thorough discussion of asymmetries observable in the MSO coordinate system, such as draping or tail twist, that would be missed in the MSE coordinate system.

Thanks for the referee's comment.

Analysis of magnetic field variation has been present in the MSO coordinate in the manuscript. And there are no valid measurements in the magnetotail in this event. This study is limited to an event analysis for which no observations of draping or tail twist asymmetry can be obtained.

Most of previous studies from single-orbiter measurements are generally based on the assumption that the IMF remains constant within a single orbit. However, given that the period of satellites is usually several hours, this assumption is very limited obviously. Our results suggest that the Martian induced magnetosphere is a rapidly dynamic system that is highly sensitive to change of direction of external interplanetary magnetic field. It indicates that conclusions from previous studies, such as draping and tail twist asymmetries in the magnetotail, should be reconsidered carefully if the effect of IMF rotation is involved.

We added more descriptions about these asymmetries in discussions in the revised manuscript.

Reply to Reviewer #2:

The authors have addressed all of the scientific comments from my previous review. Although there are still numerous grammatical errors and some statements with unclear allusion, the paper is scientifically ready for publication upon careful proof-reading and appropriate editorial corrections.
Thanks for the referee's comments and suggestions. The manuscript was carefully revised under the help of American Journal Experts (AJE).

Minor additional comment:

Line 45-47: Note that this only describes the AC component of the electrodynamic interaction, however, only the DC component will be left when averaging over time (such as the "steady-state" magnetic field structure in the empirical model used in the manuscript).
Thanks for the referee's reminding. As pointed out by the referee, the induced field is related to the AC component and the draping field is related to the DC component or 'steady-state' component, which has not been introduced in the last manuscript. In the revised manuscript, we added the descriptions of the draping field (DC component) in the Introduction section.

Reply to Reviewer #3:

Round 2 Review of Lin et al., 2024 "Direct Observations of Rapid Response of Martian Induced

Magnetosphere to Interplanetary Magnetic Field Rotation"

Overview:

I thank the authors for their response to my concerns. The following are remaining items that I still recommend addressing for review.

Major - -

1. The manuscript still has several syntax issues throughout the current manuscript. I urge the authors to consider pursuing an external review solely for syntax. The current manuscript is difficult to review fully in its current iteration. This was similarly suggested by other reviewers. For example:

§ Direct Observations of Rapid Response of Martian Induced Magnetosphere to Interplanetary Magnetic Field Rotation

• Martian should be replaced with Mars', an 'a' should be inserted before Rapid Response, an "an" should be inserted before Interplanetary

§ Induced magnetosphere in non-magnetized planets or moons, formed by the interaction between its atmosphere/ionosphere and stellar wind or planetary plasma flows, is generally affected by external magnetic field.

• This sentence is very unclear and should be rewritten for content and syntax.

• It could be rewritten as "Induced magnetospheres of non-magnetized planets or moons are formed by the interaction between a planet's atmosphere and ionosphere and solar wind."

Thanks for the referee's comments and suggestions. The manuscript was carefully revised under the help of American Journal Experts (AJE).

2. Line 131, double check these times and claims?

The solar wind is still disturbed (IMF Bx spike) at 23:09 and MAVEN seems to respond to this at 23:12. This is worth additional text as it's one of the most obvious 'by-eye' correlations.

Thanks to the reviewer's suggestions. As pointed out by the referee, there is a small perturbation in the B_x component of the IMF between 23:09-23:10 UT, and a small perturbation in the B_x component of the magnetic field in the magnetosphere between 23:09-23:10 UT. The latter can be identified as the result of the response to the former based on the similarity of the two waveforms. The time difference between these two small amplitude perturbations can be roughly regarded as their recovery time (~188-221 seconds), which is very close to our estimate of the recovery time of the main change. This is another proof that our estimated recovery times are plausible.

The relevant description was added to the revised manuscript at Lines 196-202.

Minor - - -

Line 22 is unclear; I am unsure of what this sentence is conveying.

Thanks for the referee's comment. We rewrote the sentence in the revised manuscript.

The revised sentence is "Interaction between ionosphere of the non-magnetized planets/moons and stellar wind/planetary plasma flows results in induced magnetosphere. The induced magnetosphere is generally affected by external magnetic field."

Line 47, on the surface? What is this referring to?

Thanks for the referee's comment. This refers to those planets without atmosphere but with metal surface. Theoretically, the metal surface can induce electric currents when the external or interplanetary magnetic field changes.

Line 609, citation?

Thanks for the referee's reminding. It refers to several studies (e.g., Chai, L., et al. The Induced Global Looping Magnetic Field on Mars. The Astrophysical Journal 871, L27 (2019); Dubinin, E., et al. Induced magnetic fields and plasma motions in the inner part of the Martian magnetosphere. Journal of Geophysical Research: Space Physics 126, e2021JA029542 (2021); Azari, A. R., et al. Magnetic field draping in induced magnetospheres: Evidence from the MAVEN mission to Mars. Journal of Geophysical Research: Space Physics 128, e2023JA031546 (2023)).

It should be mentioned that we removed the correlation method in the revised manuscript according to the reviewer #1's comment.

Reply to the Reviewers

We greatly thank the reviewers for the valuable comments/suggestions that we considered in the revised manuscript. All revised parts are marked in blue in the text. Detailed answers to the comments are given below:

Reply to Reviewer #4:

This study provides two case studies of how the Mars magnetosphere and ion plume respond to the upstream IMF rotation. One case was to characterize the magnetic field response at 300-500 km altitude (dayside ionosphere), and the other case was to characterize the ion plume at >2000 km (magnetosheath). This study concludes the response time for the two cases is about 160-200 s.

Main comments:

Regarding the first case, I have a couple of concerns.

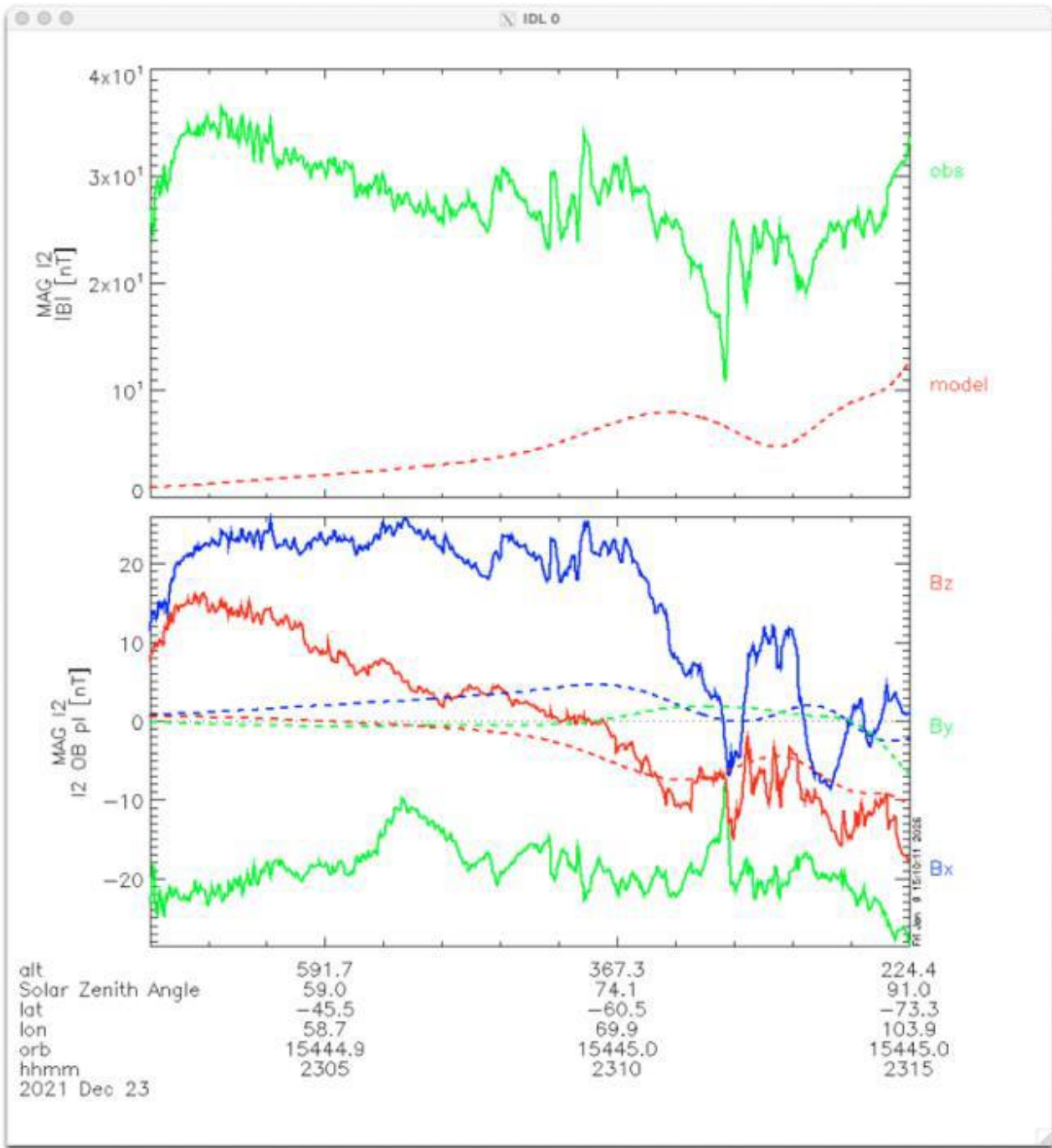

(1) The manuscript states that because this orbit is over a very weak crustal field, the crustal field effect can be ignored. This is not necessarily true when the region of interest is down to 300 km altitude. The figure below is the comparison of the measured magnetic field and the magnetic field components from the MAVEN observations (solid lines) and a crustal field model [Morschhauser et al, 2014, JGRE] (dashed lines). Particularly, in panel 2, the Bz component from the observation (red-solid) matches decently with the modeled Bz component (red dashed) from 23:10 and onward. The clock angle (tan-1By/Bz) variation in the data is mainly caused by the change in Bz, as By remains roughly constant at -20 nT for the time of interest. In this case, the Bz change might be mainly because the crustal field starts to dominate, rather than the upstream IMF rotation.

Thank you for this important comment. We agree that the original manuscript did not provide

sufficient evidence to justify the treatment of the crustal magnetic field effect in this altitude range. In particular, we agree that at altitudes of a few hundred kilometers, the crustal magnetic field cannot be dismissed a priori. We have therefore substantially revised this part of the manuscript.

To directly assess whether the observed magnetic-field variation in this event is dominated by the upstream IMF rotation or significantly affected by the crustal magnetic field, we performed two sets of new 3-D hybrid simulations using the RHybrid platform (Jarvinen et al., 2022; Zhou et al., 2026): one set including Mars' crustal magnetic field and one set without it. For each set, we carried out two runs corresponding to the upstream conditions before and after the IMF rotation. A description of the simulation setup has been added to the Methods section of the revised manuscript in lines 647-674, and the corresponding results are shown in Figure A1.

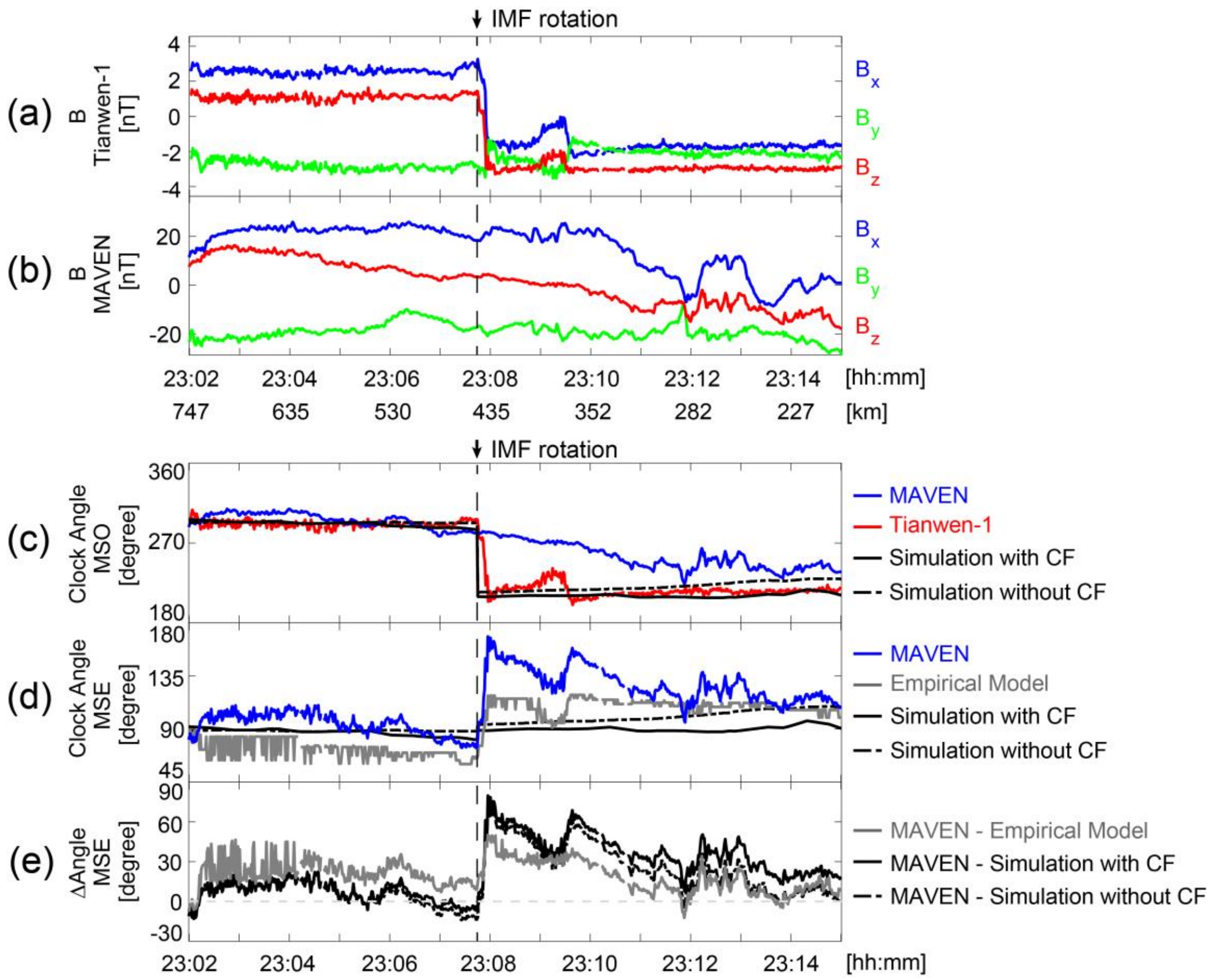

Figure A1. Magnetic field measurements by MAVEN in the magnetosphere accompanying by the interplanetary magnetic field measurements by Tianwen-1 in the solar wind, and associated results from the empirical model and the hybrid simulations. (a-b) Magnetic field in MSO coordinates by Tianwen-1 and by MAVEN, (c) clock angle of magnetic field measurements and the hybrid simulations in MSO coordinates, (d) clock angle of MAVEN measurements, the empirical model and the hybrid simulations in MSE coordinates, (e) difference of clock angles in MSE coordinates. In panel (c), red and blue lines represent magnetic field measurements in MSO coordinates by Tianwen-1 and by MAVEN, and the black solid and dashed lines represent magnetic field from the hybrid simulation with crustal fields and that with no crustal fields, respectively, in MSO coordinates. In panel (d), the blue line represents magnetic field measurements by MAVEN in MSE coordinates,

the grey line represents magnetic field from the empirical model in MSE coordinates, and the black solid and dashed lines represent magnetic field from the hybrid simulation with crustal fields and that with no crustal fields, respectively, in MSE coordinates. In panel (e), the grey line represents difference of clock angle of magnetic field between MAVEN and the empirical model in MSE coordinates, the black solid and dashed lines represent difference of clock angle of magnetic field between MAVEN and the hybrid simulation with crustal fields and that with no crustal fields, respectively, in MSE coordinates.

These simulations (Figure A1c-A1d) show that:

(1) Both simulation sets reproduce the observed change in the magnetospheric magnetic-field direction between the pre- and post-rotation intervals. This indicates that the field deflection observed by MAVEN is consistent with the upstream IMF change.

(2) Above ~200 km altitude, the simulation results with and without crustal magnetic fields are very similar, and both reproduce the same overall field-direction change inferred from the observations. Their close agreement indicates that, in this event and in this altitude range, the crustal-field contribution is smaller than the IMF-driven contribution.

(3) After 23:10:25 UT, the direction of the magnetic field in the simulation included the crustal magnetic fields starts to exhibit a different variation trend from that without the crustal magnetic fields. This confirms that the crustal magnetic field indeed exerts non-local effects on Mars' induced magnetosphere, yet these effects remain secondary to the IMF-driven variation above ~200 km altitude in this case.

We further compared the magnetic-field components and normalized magnetic-field components from the simulations with the MAVEN observations (Figure A2/Supplementary Fig. 2). This comparison shows that the two simulation sets remain close to each other in both field magnitude and direction over most of the interval, further supporting the conclusion that the crustal magnetic field is not the dominant factor above ~200 km in this event. The remaining difference that develops after 23:10:25 UT in the simulation including crustal fields is consistent with a secondary non-local modulation by the crustal magnetic field.

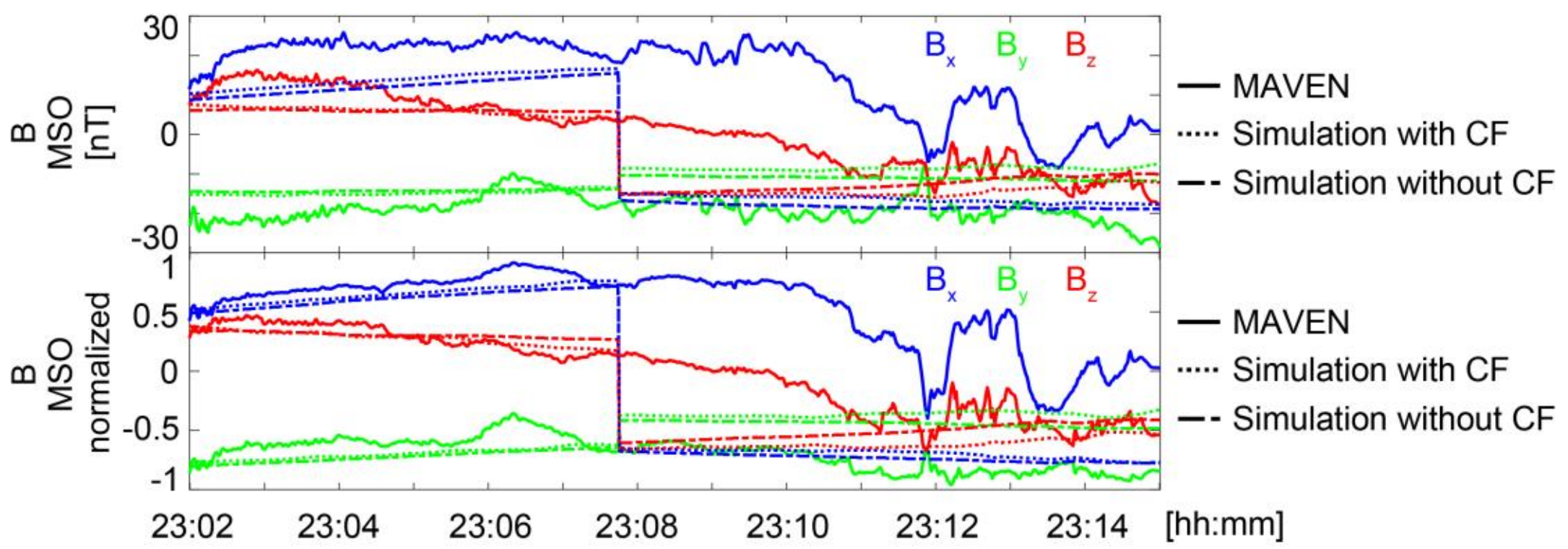

Figure A2. Comparison of magnetic field and normalized magnetic field components in MSO coordinates between MAVEN observations and the simulations. The solid curves represent the magnetic field components of MAVEN observations. The dotted curves represent the magnetic field components of the simulation with the crustal magnetic fields. The dashed curves represent the magnetic field components of the simulation without the crustal magnetic fields.

Based on these evidences and analyses, we no longer make the broad statement that the crustal-field effect can simply be ignored at low altitude. Instead, **we now state more carefully that, for this specific event, the crustal-field contribution is not the dominant factor and the observed magnetic field variations above ~200 km are indeed driven by the IMF rotation above ~200 km in this case, whereas at lower altitudes it could become significant.**

The apparent correspondence between the observed B_z and the crustal-field model after ~23:10 UT deserves caution. However, based on the hybrid simulations, we now interpret that resemblance not as evidence that the crustal field is driving the transition at altitudes above ~200 km, but rather as a partial similarity that does not represent the dominant cause of the field variation over the interval analyzed here.

We thank you for raising this issue. It has led us to significantly strengthen the manuscript and clarify the altitude-dependent role of the crustal magnetic field.

(2) L164-165, L172-173, and Figure 2c: Why does the modeled clock angle in Fig 2c have ~30 deg change in clock angle while the upstream clock angle changed ~100 deg? There are two things to check: (1) what does the model predict for this segment if there is no IMF rotation, i.e., using the IMF values before the rotation for 23:02-23:15; (2) It would be also informative to compare the actual magnetic components in MSE, not just the angles, for both MAVEN and the model.
Thanks for the comments and suggestions. We have provided an additional clarification about the applicability of the empirical model and added explanations for the smaller modeled clock-angle change. Following your suggestion, we performed both of the proposed checks and used the resulting comparisons to reassess the applicability of the empirical model in this event.

(a) Model behavior if there is no IMF rotation:
Following your suggestion, we recalculated the empirical-model result over the same interval using the averaged pre-rotation IMF condition as a fixed input, i.e., assuming no IMF rotation occurred. The result shows no abrupt change comparable to that observed near 23:08 UT; only a smooth variation associated with the spacecraft motion through the background field structure remains (Figure A3). This shows that the transition seen in the original model result is associated with the change in IMF orientation rather than being an artifact of the model itself. This result is now included in the revised manuscript in lines 693-703 and the supplementary information (Supplementary Fig. 10).

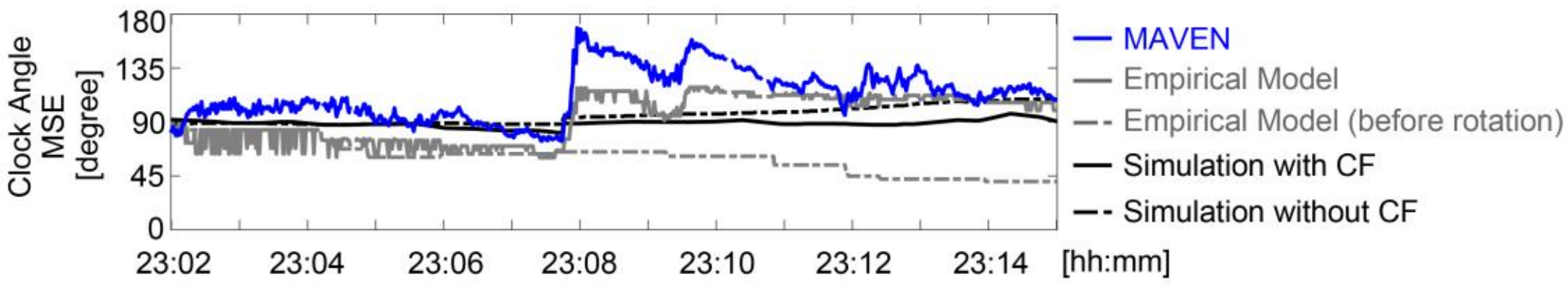

Figure A3. Clock angles of magnetic field of MAVEN observations, the empirical model and the hybrid simulations. The blue curve represents MAVEN observations. The grey solid curve

represents the empirical model and the grey dashed curve represents the empirical model assuming no IMF rotation. The black solid curve represents the hybrid simulation with crustal magnetic fields and the black dashed curve represents the hybrid simulation with no crustal magnetic fields.

(b) Comparison of magnetic-field components in MSE coordinates:

We also added a comparison of the normalized magnetic-field components (B_x, B_y, B_z) in MSE coordinates between the MAVEN observations and the empirical model (Figure A4). It should be noted that a comparison of the absolute values of the magnetic-field components is not informative in this case, because the empirical model only incorporates the direction of the upstream IMF through the coordinate transformation and the IMF magnitude is different between this case and the model. For this reason, we focus primarily on the comparison of the **normalized magnetic-field components**, with each component normalized by the corresponding total magnetic-field magnitude. The comparison shows that the empirical model captures the steady-state trend of the induced magnetospheric magnetic field reasonably well and provides a useful reference for determining when the system approaches its new steady configuration after the IMF rotation. This comparison is now included in the revised manuscript in lines 703-718 and the supplementary information (Supplementary Fig. 11). This comparison supports the use of the empirical model as a steady-state reference for the altitude range sampled in this event. In addition, the variations in the B_x and B_z components from empirical model are associated with the spacecraft traversal between the eastern and western hemispheres in the MSE coordinate system (Zhang et al., 2022, doi:10.1029/2022JE007334), and the looping magnetic field structure reported by Chai et al. (2019, doi:10.3847/2041-8213/aaff6e), respectively.

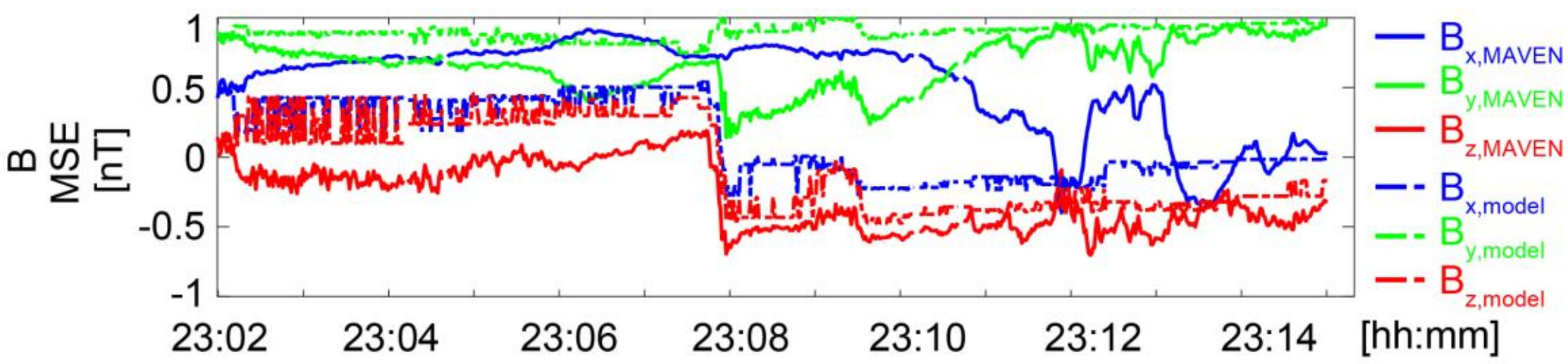

Figure A4. Comparison of normalized magnetic field components in MSE coordinates between MAVEN observations and the empirical model. The solid curves represent the magnetic field components of MAVEN observations. The dashed curves represent the magnetic field components of the empirical model.

(c) Physical reason for the smaller modeled clock-angle change:

The empirical model is constructed under the statistical constraint of a nearly steady upstream IMF, meaning that the model-predicted magnetic field values can theoretically represent the steady-state magnetic field of Mars' induced magnetosphere in the MSE coordinate. When the IMF undergoes rapid variations, the observed magnetic field in the induced magnetosphere exhibits abrupt changes in the MSE coordinate; this is not a reflection of the actual physical variation of the magnetic field itself, but rather a result of the rapid rotation of the basis vectors of the MSE coordinate. **The steady state of the magnetosphere is thus disrupted, which is the primary reason for the rapid change in the magnetic field clock angle observed by MAVEN at 23:07:45 UT.** Since the empirical

model consistently characterizes the steady-state magnetic field of the induced magnetosphere, it does not predict a large angular change at this specific time. As the perturbed IMF propagates towards Mars, the magnetic field in the induced magnetosphere begins to adjust and converges to a new steady state. Identifying the transition from the non-steady state to this new steady state requires a clear reference for the steady-state magnetic field, which the empirical model precisely provides. The empirical model is therefore not intended to reproduce the transient jump itself, but to provide the expected steady-state reference toward which the observed field evolves after the IMF rotation. This constitutes the fundamental rationale underlying the application of our empirical model approach.

We have added a clearer explanation of this point to the revised manuscript. We have also further clarified the role of the empirical model: it is intended to represent the steady-state induced magnetic field in MSE coordinates under nearly steady upstream conditions. During a rapid IMF rotation, the observed magnetospheric field temporarily departs from that steady state. The empirical model is therefore used as a reference to identify when the field has adjusted toward the new steady configuration, rather than to reproduce the transient response itself. In addition, we compared the empirical model with the new hybrid simulation results and found good agreement, especially with the simulation without crustal magnetic fields. This further supports the reliability of the empirical-model approach for this event.

L257-258: The response time should/could be different for different regions of the Mars magnetosphere, as shown in model results [Romanelli et al., 2018, JGRA]. This statement is too generalized.
Thanks for the comment. We agree with you and the original statement was too general. We have revised the manuscript to clarify that the response times reported in this study apply only to the two regions examined in the present events in lines 313-315.

L292-294: Not sure why the plume response time is calculated using the magnetic induction equation. MAVEN MAG data show that the magnetic field "stabilizes" within ~1 min in the sheath. If the ion plume does not originate from in situ production but rather from the ionosphere, the time for ions being accelerated from the ionosphere to the observational altitude also needs to be considered. More clarification is needed.
The discussions of the magnetic induction equation only focus on the response time of magnetic field but not for ion plume. Perhaps the original explanation was not sufficiently clear and could be misleading. In the revised manuscript, we now distinguish more clearly between: the response of the magnetic field configuration to the upstream IMF rotation, and the observable response of the O^+ plume at the spacecraft location. Our interpretation is that the appearance and rotation of the O^+ plume in the magnetosheath are controlled by the reconfiguration of the convection electric field associated with the IMF rotation, but the observed plume response does not depend solely on the local magnetic-field adjustment. It also involves the finite time required for planetary ions originating from lower altitudes to be redirected and transported into the observational region. This point has been clarified in the revised manuscript in lines 370-375.

As pointed out by previous reviewers, the manuscript has quite a few grammatical errors.

Thank you for the reminding. We have carefully proofread the revised manuscript and corrected numerous grammatical and stylistic issues throughout the text to improve clarity and readability.

Regarding Response to Previous Reviewers' Comments:

(1) The discussion using perturbations in Bx to estimate the recovery time, which I assume is about the paragraph in L196-202, is not quite convincing to me either. If to talk about "similar wave forms", the variations in Bx actually have Bx flip signs (Fig2b, or the 2nd panel in the figure above) a couple of times, while the upstream IMF Bx only decreases from -2 nT to ~0 nT and then back to -2 nT, no sign change involved at all (Figure 2a). Also, I assume this is a typo: "a small perturbation in the Bx component of the magnetic field in the magnetosphere between 23:09 and 23:10 UT." It should be "between 23:12 and 23:14 UT"?
Thank you for the comment. We agree that the previous discussion based on the B_x perturbation was not sufficiently convincing. In the revised manuscript, we have therefore de-emphasized this argument and no longer use it as evidence for determining the response time. Instead, the response-time estimate is now based primarily on the comparison among the MAVEN observations, the empirical steady-state model, and the hybrid simulations with and without crustal magnetic fields.

And thank you for identifying the typo. The time interval has been corrected from 23:09 – 23:10 UT to 23:12 – 23:14 UT in the revised manuscript.

(2) Regarding the discussion on crustal field effects, I have mixed feelings about both the reviewer's comments and the authors' responses. Here are my takes. I think some of Reviewer 1's questions/comments are a little nitpicking. But I also disagree with the authors' argument that crustal field effects only take place at locations at or near crustal fields. Dubinin et al. [2023] show crustal fields have a global effect, which could be minimized in MSE coordinates. This is why there is no tail twist in MSE but only in MSO. Furthermore, lots of referenced papers in the response are either pre-MAVEN or mainly focus on the global-scale structures, while low-altitude results are not thoroughly examined. I'm not against using the averaged magnetic field in MSE to approximate the induced field at Mars, but I'm concerned about its validity in low altitudes. Just as pointed out in my main point (1), it is very likely that the variation in Bz is because of crustal fields.
Thanks for the comment. We agree that crustal magnetic fields can affect Mars' plasma environment on a global scale rather than only locally, and we have revised the manuscript accordingly. We also agree that the validity of the empirical model in the MSE coordinate system, especially at low altitudes, should not be assumed without further verification. This was precisely why we introduced the new hybrid simulations in the revised analysis.

As addressed in our responses to the first two major comments, we carried out new hybrid simulations to quantify the role of the crustal magnetic field in this event and to assess the applicability of the empirical model. Based primarily on the hybrid simulations and the observations, we conclude that above ~200 km altitude the magnetic-field variation in this event is primarily controlled by the upstream IMF rotation, whereas the influence of the crustal magnetic field becomes increasingly important at lower altitudes. Under this event-specific condition, the empirical model reproduces the IMF-controlled magnetic-field configuration well above ~200 km and is

consistent with both the observations and the hybrid simulations in that altitude range.

Accordingly, we use the empirical model as a steady-state reference only in the altitude range where the IMF is dominant, while treating its applicability at lower altitudes more cautiously. These points have been clarified in the revised manuscript; please also see our responses to the major comments for further details.

Minor comments:

L22 and throughout the text: The manuscript uses both “nonmagnetized” and “unmagnetized”. To my knowledge, “unmagnetized” is more widely used. Please change it to use one term only.
Thank you for pointing this out. We have corrected this typo by replacing all instances of “nonmagnetized” with “unmagnetized” throughout the manuscript.

L46-48: This statement is only partly true. Currents can exist without the temporal variations in the upstream IMFs. In a steady state, currents flow at boundary layers.
Thanks for the reminding. We have added the description of steady-state currents in the revised manuscript in lines 42-45.

L63-64: “The induced magnetospheres of …Titan) share similar formation mechanisms but have
Corrected.

L131: “disturbance caused by in the magnetic field”
Corrected.

We have further re-evaluated the response time for the first event using the observations, the empirical model, and the newly added hybrid simulations. We define the response time as the time when the clock-angle difference between the observed field and the corresponding steady-state reference field equals zero. Using the empirical model and the hybrid simulation without crustal magnetic fields as steady-state references, the first zero crossing of the clock-angle difference occurs at 244-247 s after the IMF rotation is observed by Tianwen-1. After accounting for the estimated ~11 s propagation delay from Tianwen-1 to the induced magnetosphere boundary, the response time is revised to 255-258 s. For the simulation including crustal magnetic fields, the clock-angle difference remains finite throughout the interval, likely because the crustal field, although weaker than the IMF-driven contribution, still introduces a modest local modulation. These results suggest that the response time of the dayside induced magnetosphere near the dawn-dusk sector is on the order of ~4 min, confirming that Mars’ induced magnetosphere responds rapidly to upstream IMF changes.

We sincerely appreciate your detailed comments and constructive suggestions. They have greatly improved the rigor, clarity, and physical interpretation of our study.

Reply to Reviewer #5:

The manuscript titled "Direct Observations of the Rapid Response of the Mars' Induced Magnetosphere to an Interplanetary Magnetic Field Rotation" uses simultaneous observations from MAVEN and Tianwen-1 to estimate the response time of Mars's magnetosphere to rotations in the interplanetary magnetic field (IMF). They focus on two case studies in which Tianwen-1 was observing the solar wind and MAVEN was either within the induced magnetosphere or the magnetosheath. In both case studies, Tianwen-1 observed a rotation in the IMF, and MAVEN observed a delayed response. The first case study focuses on the magnetospheric response to this rotation by comparing MAVEN measurements to an empirical model of the induced magnetosphere in the MSE coordinate system. The second case study focused on the O+ plume response measured by MAVEN, as the rotation in the upstream IMF shifted the direction of the convective electric field, and, as a result, rotated the O+ plume into STATIC's field of view.

Reviewer 1:
In the previous iteration of the manuscript, Reviewer 1 voiced concerns mainly regarding the methodology of the empirical model of Mars' induced magnetosphere. They argue that by averaging the magnetic field observations in the MSE coordinate system in order to generate the empirical model, this methodology ignores the global impact of crustal magnetic fields on Mars' induced magnetosphere. In response to this concern, the authors have provided an argument for the use of the MSE coordinate system and the construction of the steady-state empirical model, including showing multiple global empirical maps in which measurements above strong crustal fields have been removed.

After considering both arguments, it is not clear to me that the authors have adequately addressed this concern from Reviewer 1, and I share similar reservations about this methodology. As Reviewer 1 points out, the crustal fields have a global impact of Mars' magnetosphere extends beyond their local sphere of influence (Dubinin et al., 2023, Dibraccio et al., 2022, and Guo et al., 2025 (https://doi.org/10.1029/2024GL114441). The authors have shown that their empirical maps in the MSE coordinate system do not change when "the magnetic field measurements are removed where the crustal magnetic field magnitude by model (Morschhauser et al., 2014, doi:10.1002/2013JE004555) at altitude of 185 km (Figure A2) is larger than 50 nT." If I understand this correctly, the authors have removed all MAVEN MAG measurement that fall directly above these strong crustal field regions. However, the influence of crustal fields also extends in latitude and longitude (Guo et al., 2025, de Oliveira et al., 2021 (https://doi.org/10.1029/2021JA029242)., as well as out in the magnetotail (DiBraccio et al., 2022).

The authors argue that, according the Dubinin et al., (2023), "the crustal field is reconnected to the IMF and thus affects the original draping field, which means that the effect of the crustal field on the Martian magnetosphere is limited to regions close to the crustal field and, as an important prerequisite, it must be reconnected to the IMF." I would argue that because crustal fields reconnect with the IMF, this ensures that they have an explicitly non-local effect on the draping, since the generation of open field lines, which also drape around the planet, must have one end attached to

the crust of the planet, altering the draping pattern of the field lines.

In short, I also have concerns regarding the use of this steady-state empirical model, and do not think this methodology is properly justified. In the current form of this study, the estimate of the response time of the magnetosphere heavily depends on the "agreement vs. disagreement" between MAVEN MAG measurements and this empirical model. This agreement is based on a model that ignores some key physics of the system, and I would argue the difference between measurement and model is likely quite sensitive to factors other than upstream IMF rotation. For instance, even in the small time series shown in Figure 2d, the clock-angular difference between MAVEN and the model exceeds 20 degrees at ~23:05, which is only slightly less than the change measured at 23:08, which theoretically is the solely the result of the IMF rotation. One suggestion to improve the study could be a comparison between MAVEN measurements and an MHD simulation of Mars' magnetosphere under these two different IMF orientations (pre-and post-rotation). This would ensure that the local time and crustal field effects are captured in the study and would enable more convincing methodology.
We sincerely thank you for your insightful comments and suggestions. We appreciate your concerns and agree with your perspectives. Based on your comments and suggestions, we have conducted a series of hybrid simulation experiments and supplementary analyses. It should be noted that although we did not add an MHD simulation in the revised study, we performed new 3-D hybrid simulations, which are also well suited for investigating the response of Mars' induced magnetosphere to IMF variations. The hybrid simulations were implemented based on the RHybrid three-dimensional hybrid simulation platform (Jarvinen et al., 2022; Zhou et al., 2026), and a detailed description of this platform has been added to the Methods section of the revised manuscript in lines 647-674. For the study of Mars' magnetospheric response to IMF variations, hybrid simulations provide an appropriate physics-based framework.

To investigate whether the crustal magnetic field exerted a significant effect in this event, we conducted two sets of hybrid simulations, one set including Mars' crustal magnetic fields and the other without it. Each set consists of two simulation runs corresponding to the pre- and post-IMF rotation states, with the pre-rotation results referring to the period before 23:07:45 UT and the post-rotation results to that after 23:08:00 UT. The corresponding results are shown in Figure B1.

Both sets of simulations reproduce the observed change in the magnetospheric magnetic-field direction between the pre- and post-rotation intervals, indicating that the field deflection observed by MAVEN is consistent with the upstream IMF rotation. The two simulation sets are also close to each other above ~200 km altitude, which shows that in this event and in this altitude range the crustal-field contribution is secondary to the IMF-driven variation. After 23:10:25 UT, the simulation including crustal magnetic fields begins to deviate slightly from that without crustal magnetic fields, indicating an additional weak crustal modulation that does not dominate the overall transition. These results suggest that, for this specific event, the observed magnetic-field variation above ~200 km is primarily driven by the IMF rotation, whereas the crustal-field contribution may become more important at lower altitudes.

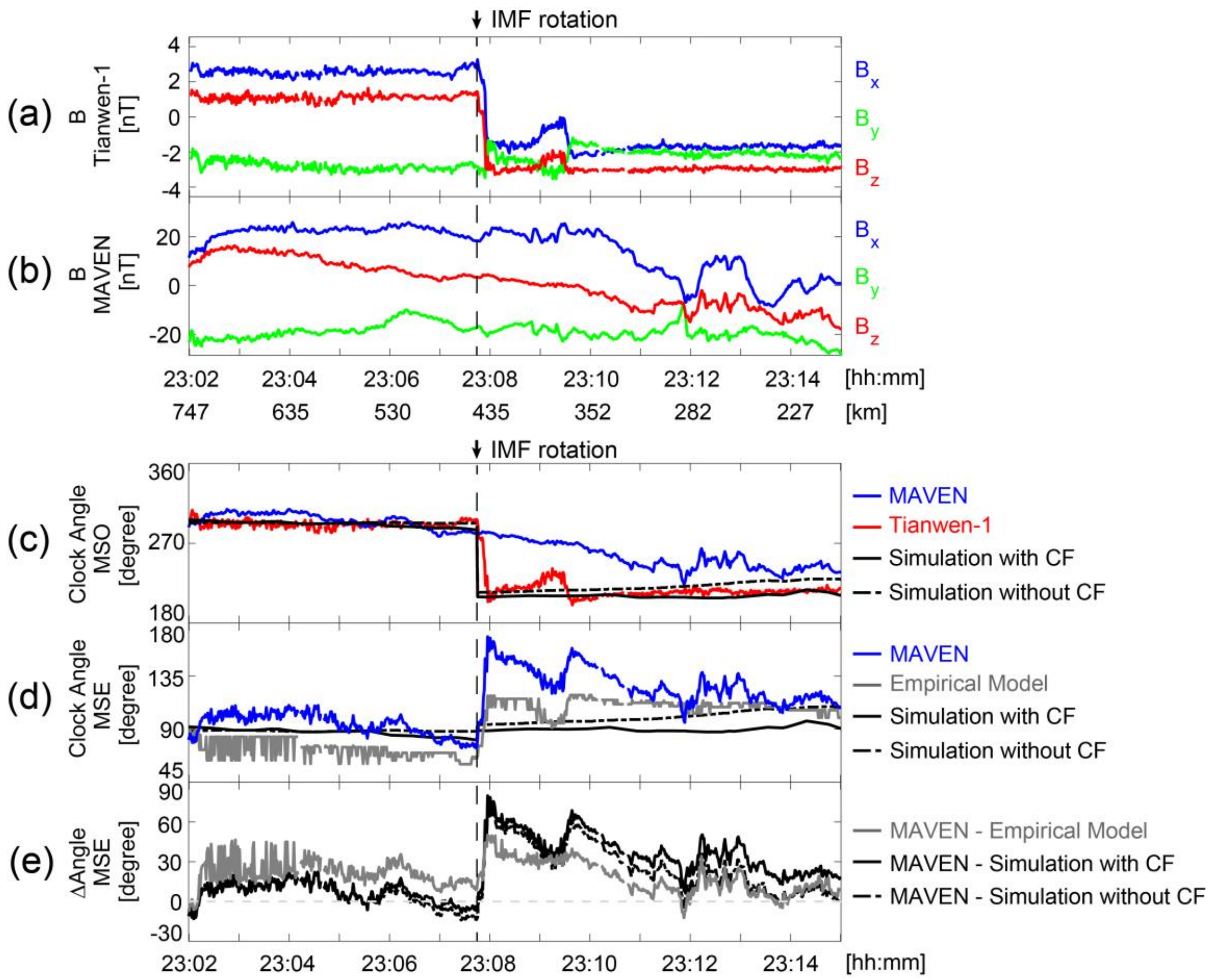

Figure B1. Magnetic field measurements by MAVEN in the magnetosphere accompanying by the interplanetary magnetic field measurements by Tianwen-1 in the solar wind, and associated results from the empirical model and the hybrid simulations. (a-b) Magnetic field in MSO coordinates by Tianwen-1 and by MAVEN, (c) clock angle of magnetic field measurements and the hybrid simulations in MSO coordinates, (d) clock angle of MAVEN measurements, the empirical model and the hybrid simulations in MSE coordinates, (e) difference of clock angles in MSE coordinates. In panel (c), red and blue lines represent magnetic field measurements in MSO coordinates by Tianwen-1 and by MAVEN, and the black solid and dashed lines represent magnetic field from the hybrid simulation with crustal fields and that with no crustal fields, respectively, in MSO coordinates. In panel (d), the blue line represents magnetic field measurements by MAVEN in MSE coordinates, the grey line represents magnetic field from the empirical model in MSE coordinates, and the black solid and dashed lines represent magnetic field from the hybrid simulation with crustal fields and that with no crustal fields, respectively, in MSE coordinates. In panel (e), the grey line represents difference of clock angle of magnetic field between MAVEN and the empirical model in MSE coordinates, the black solid and dashed lines represent difference of clock angle of magnetic field between MAVEN and the hybrid simulation with crustal fields and that with no crustal fields, respectively, in MSE coordinates.

We would like to provide an additional clarification regarding the validity of the empirical model approach. We use the empirical model only as a reference for the expected steady-state magnetic-field configuration in MSE coordinates. When the IMF rotates rapidly, the abrupt change seen in the observed magnetic-field clock angle in MSE coordinates mainly reflects the sudden rotation of

the MSE basis vectors, rather than an instantaneous physical reconfiguration of the local magnetospheric field. The role of the empirical model is therefore not to reproduce this transient jump, but to provide the steady-state reference toward which the observed field evolves after the IMF rotation.

To assess the applicability of the empirical model in this event, we compared its results with those of the 3D hybrid simulations (Figure B1d). The empirical model is in good agreement with the hybrid simulation outputs, especially with the simulation without crustal magnetic fields, indicating that it can represent the steady-state magnetic-field configuration associated with the corresponding IMF orientation. Within the altitude range relevant to this event, this comparison suggests that the empirical model is mainly controlled by the IMF rather than by crustal magnetic fields. These results support the use of the empirical model as a steady-state reference in the present event.

Reviewer 3:
Reviewer 3 had concerns about syntax. The examples this reviewer provided have been essentially fixed in the new manuscript, but some issues in syntax and language still persist. The paper is readable, so this may be able to be fixed by the editor, but it is still worth noting that there are still several syntax issues that should be resolved.

Reviewer 3 also argued “The solar wind is still disturbed (IMF Bx spike) at 23:09 and MAVEN seems to respond to this at 23:12. This is worth additional text as it’s one of the most obvious ‘by-eye’ correlations.”

The authors address this in Lines 198-204. However, I believe there is still a mistake here. The perturbation in the Bx component in the magnetosphere occurs at 23:12-23:13, not 23:09 as the current manuscript claims.
Thank you for identifying the typo. The time interval has been corrected from 23:09 – 23:10 UT to 23:12 – 23:14 UT in the revised manuscript.

We have further re-evaluated the response time for the first event using the observations, the empirical model, and the newly added hybrid simulations. We define the response time as the time when the clock-angle difference between the observed field and the corresponding steady-state reference field equals zero. Using the empirical model and the hybrid simulation without crustal magnetic fields as steady-state references, the first zero crossing of the clock-angle difference occurs at 244-247 s after the IMF rotation is observed by Tianwen-1. After accounting for the estimated ~11 s propagation delay from Tianwen-1 to the induced magnetosphere boundary, the response time is revised to 255-258 s. For the simulation including crustal magnetic fields, the clock-angle difference remains finite throughout the interval, likely because the crustal field, although weaker than the IMF-driven contribution, still introduces a modest local modulation. These results suggest that the response time of the dayside induced magnetosphere near the dawn-dusk sector is on the order of ~4 min, confirming that Mars’ induced magnetosphere responds rapidly to upstream IMF changes.

We sincerely appreciate your insightful comments and suggestions. They have greatly improved the rigor, clarity, and physical interpretation of our study.

Reply to the Reviewers

We greatly thank the reviewers for the valuable comments/suggestions that we considered in the revised manuscript. All revised parts are marked in blue in the text. Detailed answers to the comments point-by-point are given below:

--

Reply to Reviewer #4:

(1) The results of the hybrid simulations are not quite convincing. The clock angle in MSO from the simulations with crustal fields only has a very smooth long-time-scale variation. I'm not convinced that these simulations can reproduce the fine structures of crustal fields at low altitudes. It's likely that: either the grid resolution of the hybrid model is too coarse to capture the small-spatial-scale variations or the crustal field model used is not accurate enough.

We thank the reviewer for this important comment. To address the two possible explanations suggested by the reviewer, namely the limited spatial resolution of the hybrid model and the possible uncertainty of the crustal field model, we re-examined the crustal magnetic field implemented in the hybrid simulations.

Firstly, we compared the initial magnetic field used in the hybrid model along the virtual spacecraft trajectory with the magnetic field directly predicted by the crustal field model, before significant interaction with the solar wind develops, as shown in Figure A1. This comparison allows us to evaluate how well the crustal field is represented in the simulation itself. At low altitudes, especially below about 500 km, the magnetic field strength in the hybrid model is slightly smaller and smoother than that predicted by the full crustal field model. This is expected because the crustal field model has a maximum spherical harmonic degree of 110, whereas the crustal field implemented in the hybrid simulation is effectively truncated to approximately degree 60–80 and is also represented on a finite numerical grid. Therefore, the smallest-scale crustal magnetic structures are inevitably smoothed in the hybrid simulation.

Nevertheless, the main magnetic field components and their large- to intermediate-scale variations along the virtual trajectory are generally consistent between the hybrid model and the crustal field model. More importantly for the present study, the simulated B_z component and the MSO clock angle agree reasonably well with those calculated directly from the crustal field model. We note that after approximately 23:14 UT, corresponding to altitudes below about 230 km, relatively large differences appear in the magnetic field direction, especially in the B_x component. However, because the MSO clock angle is mainly determined by the transverse field components in the Y–Z plane, this discrepancy in B_x has only a limited influence on the clock angle comparison.

Secondly, to test whether the result depends strongly on the choice of crustal field model, we compared the magnetic fields predicted by three independent crustal field models, M2014 (Morschhauser et al., 2014, doi:10.1002/2013JE004555), L2019 (Langlais et al., 2019, doi:10.1029/2018JE005854), and G2021 (Gao et al., 2021, doi:10.1029/2021EA001860), along the same virtual trajectory (Figure A3). The three models produce very similar magnetic field variations

for this event. This indicates that the G2021 crustal field model used in our simulations provides a reliable representation of the crustal magnetic field along this trajectory, and that the smoothness seen in the hybrid simulation is not primarily caused by an inaccurate crustal field model.

Therefore, **we agree with the reviewer that the hybrid model cannot fully reproduce all scale crustal magnetic structures at very low altitudes** because of the finite grid resolution and the truncation of the crustal field representation. **However, our comparison shows that the model does reproduce the main crustal field configuration and the clock-angle variation relevant to this event.**

We have revised the manuscript to clarify this limitation and to avoid implying that the hybrid model resolves all fine-scale crustal field structures. The revised text now states that the simulation captures the large- and intermediate-scale crustal field effects important for the present analysis, while the highest-order small-scale features may be smoothed.

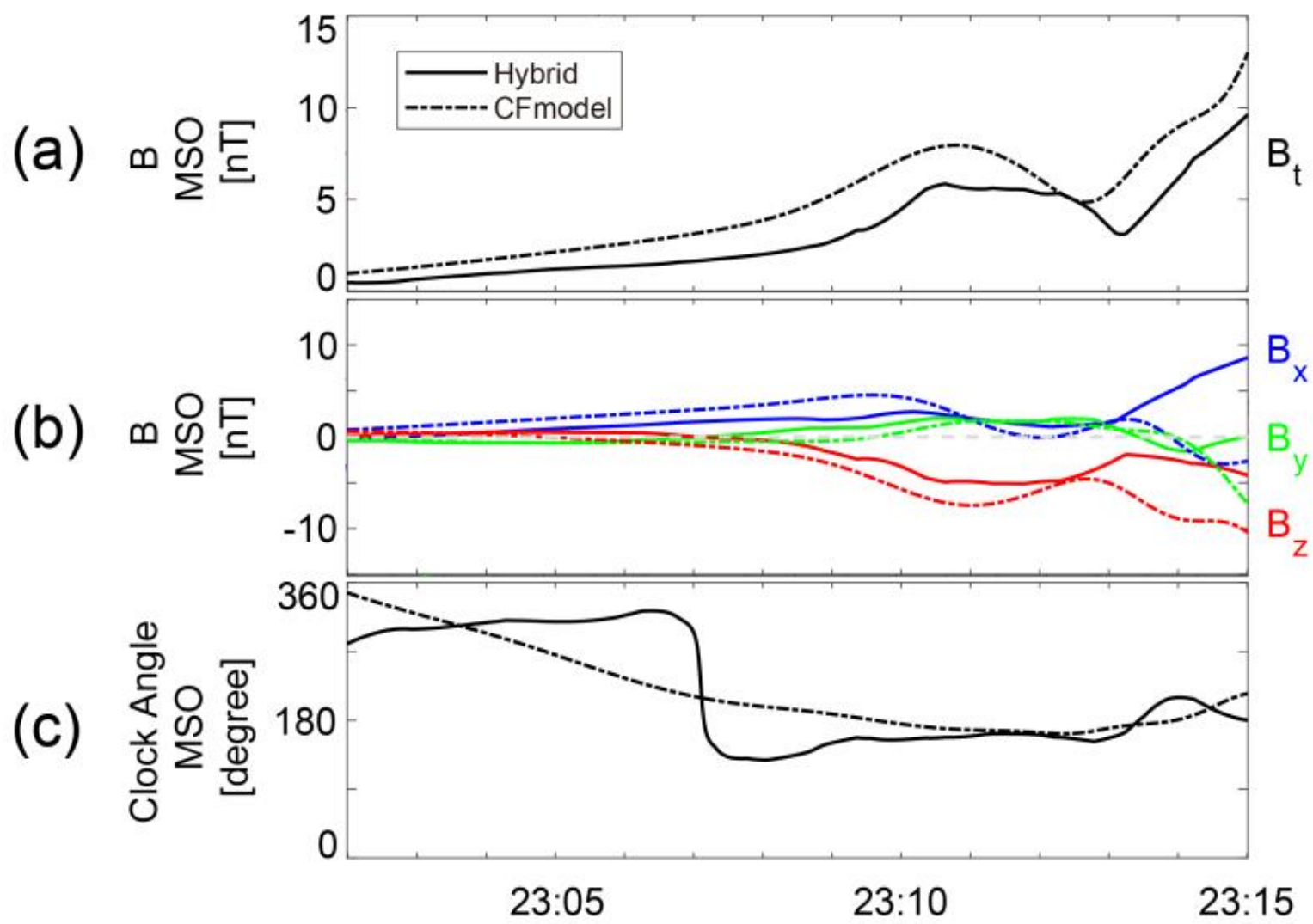

Figure A1. Comparison between the initial magnetic field in hybrid model along the virtual spacecraft trajectory with magnetic field predicted by the crustal field model M2014 (i.e., CF model here).

(2) To examine the crustal field effect, I can simply use the crustal field model Bz component to calculate the clock angle. The 3rd panel in the figure below shows the calculated clock angle using the measured By & measured Bz (Bclock, blue) and measured By & Bz from the crustal model (Bclock_crustal, orange). Bclock_crustal matches well with Bclock from ~23:10 onward, suggesting that the clock angle past 23:10 is dominated by crustal field Bz. I still think the effect of crustal fields makes it extremely difficult to determine the recovery time for this case study.
We thank the reviewer for this comment. To further evaluate whether the observed magnetic signature after ~23:10 UT was mainly caused by the crustal field, we carried out additional analyses using three independent lines of evidence: (1) statistical comparisons of repeated MAVEN observations at the same location and altitude, (2) direct comparison between the observations and several crustal field models, and (3) hybrid simulations with and without crustal fields. These analyses consistently indicate that the crustal field contribution at this location is minor, and that the

observed magnetic variation is primarily driven by the upstream IMF change rather than by local crustal fields.

1. A single-event agreement in B_z is not sufficient to demonstrate crustal-field dominance

We agree that the similarity between the observed $B_{z,\mathrm{MAVEN}}$ and the crustal-model $B_{z,\mathrm{model}}$ after ~23:10 UT is interesting and deserves examination. However, this agreement alone is not sufficient to conclude that the field during this interval was dominated by crustal magnetism.

Crustal magnetic fields are fixed with respect to Mars and should therefore produce a persistent signature at the same geographic location and altitude. Based on this idea, we examined MAVEN magnetic field measurements from repeated passes through the same region and compared them with the predicted crustal field. If the local field were strongly controlled by the crustal field, then the statistical mean or median of the observed field at that location should be close to the crustal-field prediction.

For this comparison (Figure A2), the local coordinate system is more appropriate than MSO coordinate, because the crustal field is naturally organized with respect to the local radial/tangential directions. In our event, the dominant component of the modeled crustal field is the radial component B_r. The crustal field model predicts a positive B_r of about 5 nT, whereas the mean and median of the observed B_r at the same location and altitude are both close to zero, with a broad range of variability. Therefore, the statistical characteristics of the observations are not consistent with a crustal-field-dominated environment at this location.

This suggests that the apparent agreement between the observed and modeled B_z in this single case is most likely coincidental, rather than evidence that the magnetic field was controlled by the crustal field.

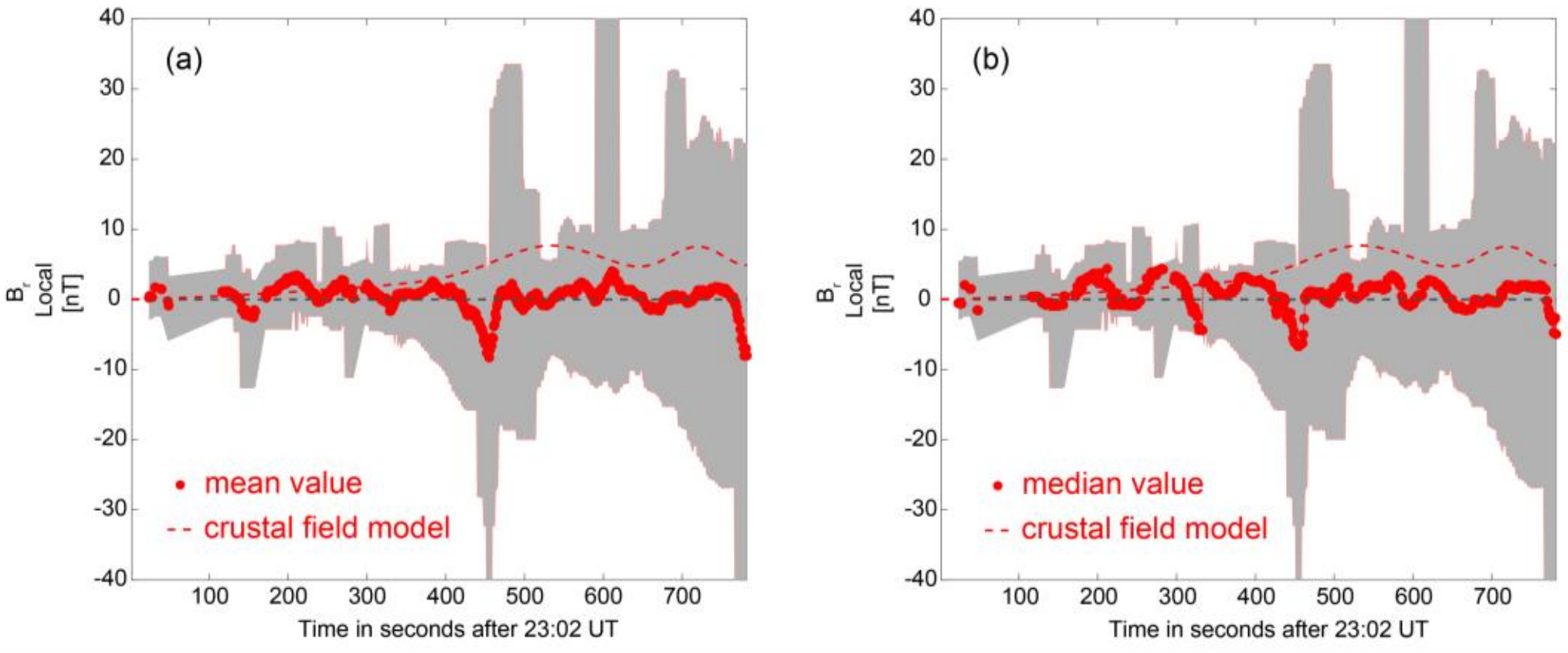

Figure A2. Statistical results of MAVEN magnetic field at the same geographic location and altitude. The shadowed regions represent the range of all data. The longitude range is within ±0.5 degrees, the latitude range within ±0.5 degrees, and the altitude range within ±10 km.

2. The crustal field models do not reproduce the overall observed magnetic vector

We also reproduced the magnetic field predictions from three crustal field models: M2014, L2019, and G2021 (Figure A3). All three models give similar results along this trajectory, so the conclusion

does not depend on the particular model selected.

The comparison with observations shows the following:

(a) Magnetic Field magnitude:

The observed magnetic field magnitude is more than twice the modeled crustal-field magnitude. This indicates that the dominant contribution comes from draped or induced magnetic fields rather than from the crustal field alone.

(b) Magnetic Field components:

In the crustal field models, B_z is the main component and B_y is very small. In contrast, the observed field is dominated by B_y. In addition, the modeled B_x and B_y components have polarities opposite to those observed. Thus, the crustal field models do not reproduce the main vector characteristics of the measured magnetic field.

(c) Clock angle interpretation:

The clock angle is determined jointly by B_y and B_z. The reviewer suggested that one may use the crustal-model B_z together with the measured B_y to calculate the clock angle. We agree that this construction can serve as a useful qualitative test, but by itself it cannot demonstrate crustal-field dominance. Because the modeled B_y is both very small and opposite in polarity to the observed B_y, the apparent agreement in clock angle mainly reflects the similarity in B_z only, rather than agreement of the full magnetic vector. Therefore, the similarity in clock angle does not necessarily imply that the observed field after ~23:10 UT was controlled by crustal fields.

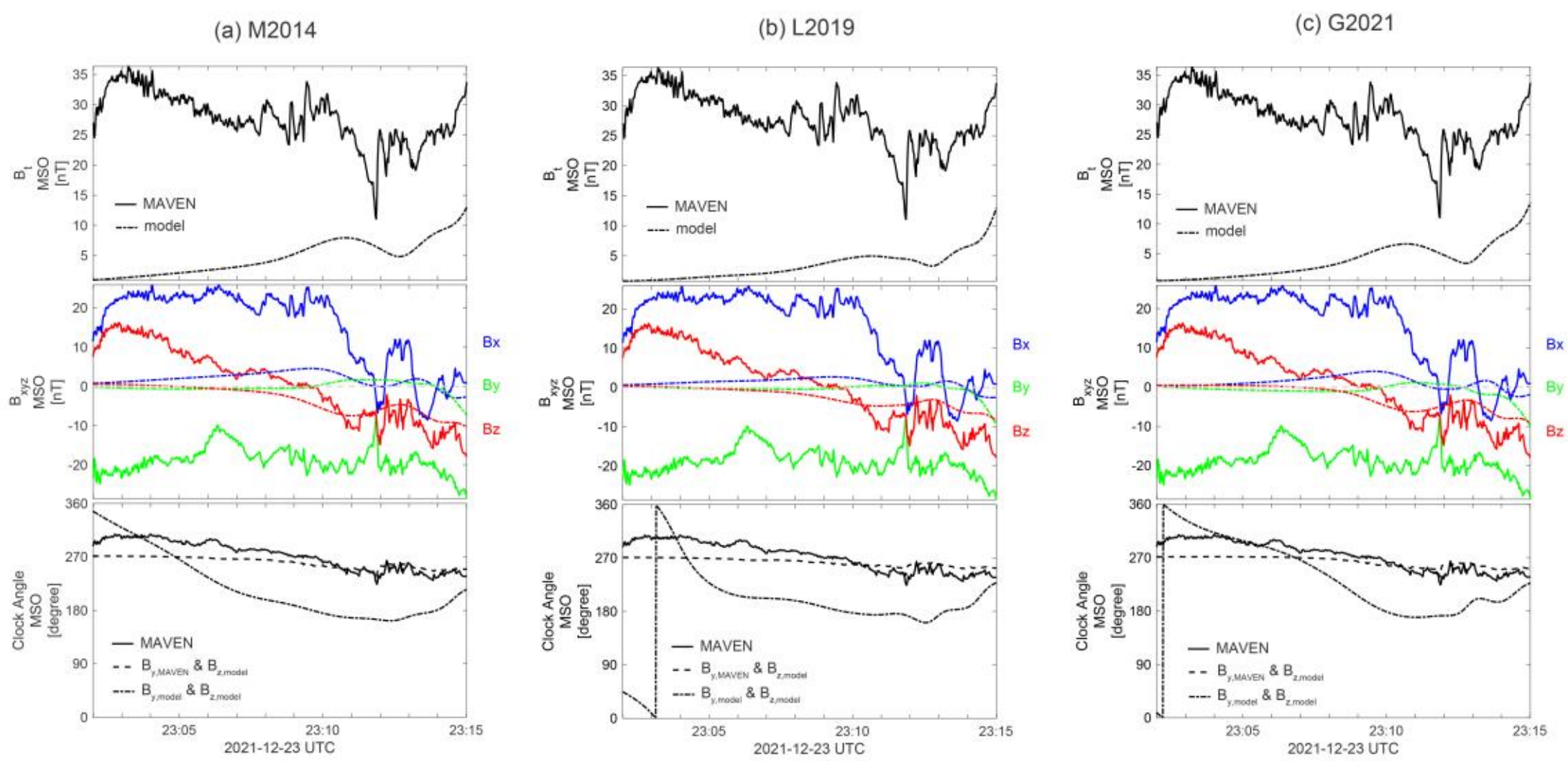

Figure A3. Comparison of results from different crustal field models.

3. Simulations with and without crustal fields show that the crustal contribution is small

Finally, our hybrid simulations directly test the crustal-field effect. We compared simulation results obtained with crustal fields included and excluded. The two runs show that the magnetic variation at the spacecraft location changes only slightly when crustal fields are removed (Figure 2c-2e), demonstrating that the crustal contribution in this event is limited. Instead, the main magnetic variation is reproduced as a response to the IMF rotation. This conclusion is further supported by

the steady-state MSE model results.

Based on all of the above evidence, we conclude that:
1. The crustal field models do not reproduce the observed magnetic field well at this location, especially for the B_y component, whose magnitude and polarity are both inconsistent with the observations. Since the clock angle depends on both B_y and B_z, the apparent similarity in clock angle is likely due to the partial agreement in B_z alone and does not demonstrate crustal-field dominance.

2. The comparison between hybrid simulations with and without crustal fields clearly shows that the crustal field is not the primary contributor in this event. The magnetic variation, including the recovery behavior, is mainly caused by the IMF turning. The MSE model results support the same interpretation.

We have revised the manuscript to clarify this point and to better explain why the recovery time in this case is not controlled by crustal field contamination, but instead reflects the response to the upstream IMF change.

(3) I'm still very confused by the clock angle change in MSE using the empirical model. From what I understand, the empirical model is a 3-D steady-state magnetic vector field in the MSE coordinates. When you apply an IMF clock angle change, isn't it just rotate the whole vector field by the same degree for every given point in the empirical model? I do not expect it to be like the clock angle change with MAVEN data but it should be similar to the IMF clock angle change? Also, Figure A4 is very puzzling. At 23:08, the Bx and By components from the empirical model, similar to Bz,maven, but By,model stays about the same. Isn't IMF clock angle change mainly causing changes to By & Bz components but not Bx?
We thank the reviewer for pointing out this confusion. We agree that our previous description of the empirical MSE model was not sufficiently clear, and we have revised the text to better explain how the model is applied to the MAVEN observations.

The empirical model is a steady-state three-dimensional magnetic vector field organized in MSE coordinates. Applying a different IMF clock angle does not simply rotate the whole magnetic vector field by the same angle at each fixed model point. Instead, **for each MAVEN measurement, we first use the observed upstream IMF to define the corresponding MSE coordinate system. The spacecraft position measured in MSO coordinates is then transformed into this IMF-dependent MSE coordinate system. The empirical magnetic field is obtained by sampling the model at this transformed MSE position.**

Therefore, when the IMF clock angle changes, two effects occur simultaneously. First, the orientation of the MSE coordinate system changes. Second, the same spacecraft position in MSO coordinates corresponds to a different location in the MSE coordinate. As a result, the model samples different regions of the three-dimensional steady-state magnetic field structure. **The modeled magnetic variation is therefore not equivalent to a simple rotation of the magnetic vector at a fixed point, and the modeled clock angle is not expected to exactly follow the IMF clock angle.**

This also explains why changes can appear in the modeled B_x component. If one only rotated a magnetic vector at a fixed MSE position, the IMF clock-angle change would mainly affect the B_y and B_z components. However, in our empirical-model calculation, the sampled MSE position changes with the IMF direction, and the empirical magnetic field has spatial differences in all three components. Thus, **B_x, B_y, and B_z may all vary depending on the local three-dimensional structure of the model field.**

For this event, the IMF rotates significantly at ~23:08 UT. As a result, the MSE coordinate system is rapidly redefined, and the mapped MAVEN position in MSE coordinates changes rapidly from the $Y_{MSE} < 0$ hemisphere to the $Y_{MSE} > 0$ hemisphere (Figure A4). This change in the sampled model location leads to the component variations shown in Figure A5.

1. The relatively small change in $B_{y,model}$ is due to the fact that, along this trajectory, the draped magnetic field structure in the empirical model produces similar B_y values at the sampled locations before and after the IMF rotation. The abrupt change in $B_{y,MAVEN}$ at ~23:08 UT was caused by the rapid change of the MSE coordinate system and the corresponding rapid change of the mapped spacecraft position in MSE coordinates. Subsequently, $B_{y,MAVEN}$ gradually approaches $B_{y,model}$, which is consistent with the response to the IMF rotation.

2. $B_{x,model}$ changes more clearly because the spacecraft samples different sides of the draped magnetic field structure in MSE coordinates (Figure A4a). The observed $B_{x,MAVEN}$ component later shows a similar polarity change, which is consistent with the response to the IMF rotation. The multiple polarity changes in $B_{x,MAVEN}$ may also be related to the spacecraft's approaching the current sheet.

3. The variation in $B_{z,model}$ reflects the non-draping component of the magnetic field, which is the looping magnetic field component in Y-*Z* plane reported in previous studies (Figure A6; Chai et al., 2019, doi:10.3847/2041-8213/aaff6e; Dubinin et al., 2019, doi:10.1029/2019GL084387; Zhang et al., 2022, doi:10.1029/2022JE007334). If the magnetic field were purely draped in the MSE frame, then $B_{z,model}$ should be zero. In this event, the mapped MAVEN position changes from the $Y_{MSE} < 0$ hemisphere to the $Y_{MSE} > 0$ hemisphere. Therefore, the empirical model predicts a polarity reversal of $B_{z,model}$, from positive to negative, as shown in Figure A4b & A5.

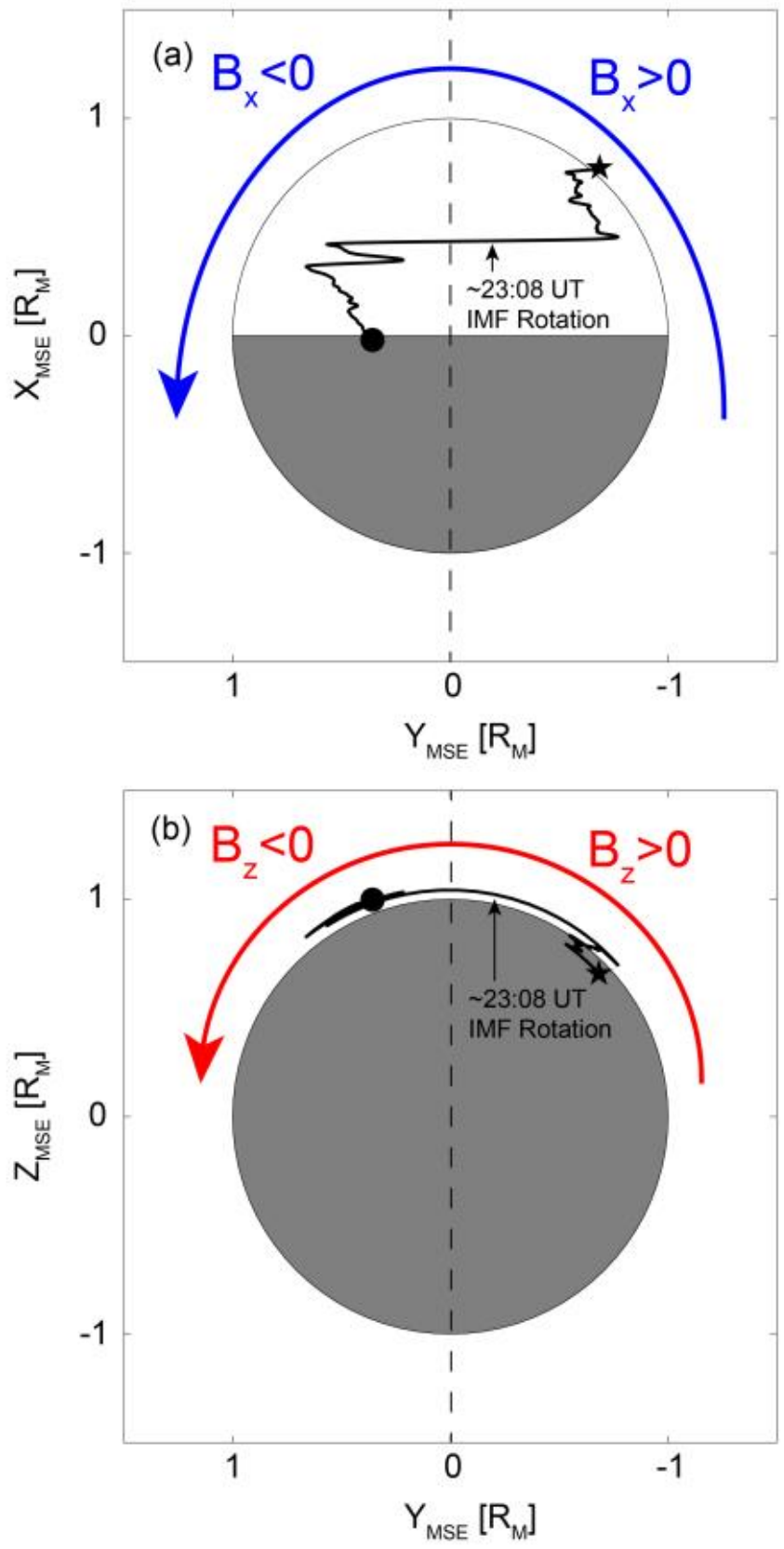

Figure A4. Position of MAVEN and illustration of magnetic field in MSE coordinate (a) in Y-X plane and (b) in Y-Z plane. The time interval of the orbit is 23:02-23:15 UT. The blue star represents the start of the orbit. The blue dot represents the end of the orbit. The black curves with arrow represent magnetic field lines.

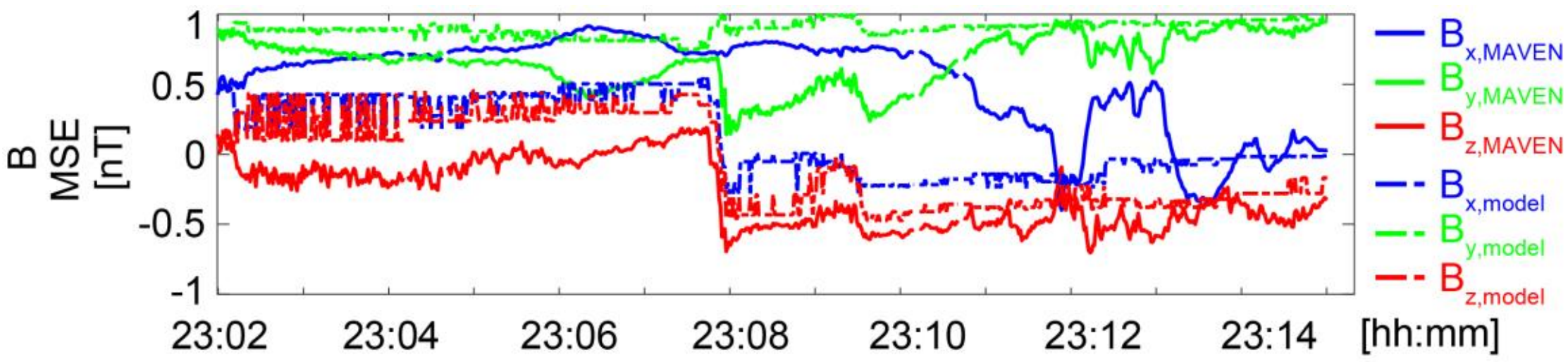

Figure A5. Comparison of normalized magnetic field components in MSE coordinates between MAVEN observations and the empirical model. The solid curves represent the magnetic field components of MAVEN observations. The dashed curves represent the magnetic field components of the empirical model.

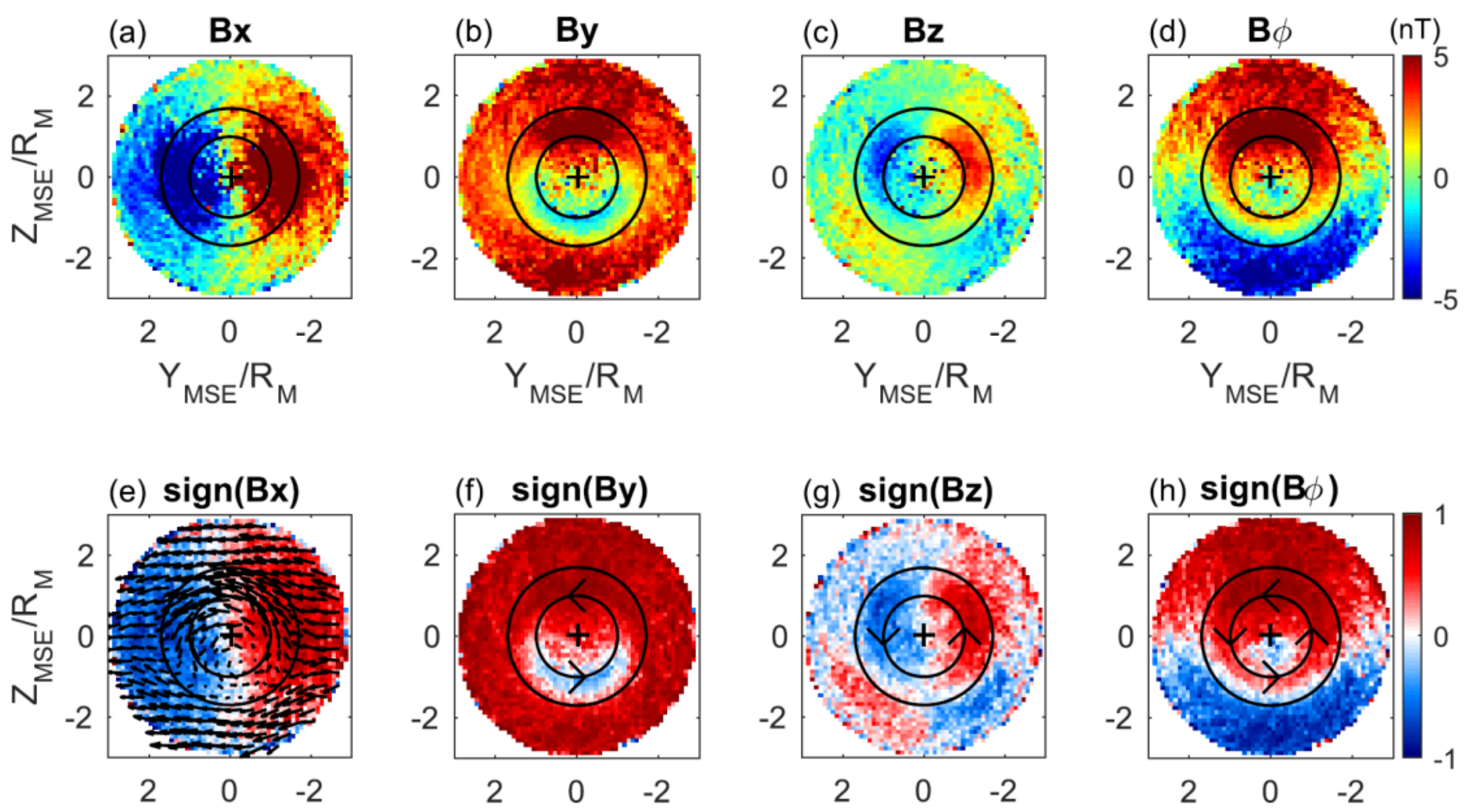

Figure A6. Magnetic field structure and the solar wind transverse velocity observed on the nightside of Mars in the MSE coordinate system, adapted from Chai et al. (2019).

We added more explanations on this model and analysis of magnetic field variations responding to changing of MAVEN position in MSE coordinate to clarify this point, and to avoid implying that the empirical-model result represents a simple rotation of the magnetic vector field.

(4) Regarding the plume response time, the revised text is still a little confusing. If I understand it correctly, the calculation in Lines 357-364 is for the time scale of ion transport from the ionosphere to the IMB before ions "see" the main convectional electric fields. If so, I would suggest putting Lines 371-376 before Line 353 and simplifying the text a little.

We thank the reviewer for the helpful suggestion. Following the reviewer's suggestion, we have moved the explanation originally in Lines 371–376 to before Line 353 and simplified the text in Lines 387-403 in the revised manuscript.

Reply to Reviewer #5:

Reviewer #5 (Remarks to the Author):

The latest version of this manuscript is an improvement to the previous versions. The inclusion of the hybrid simulations provides a useful reference for the impact of crustal magnetic fields on the low-altitude measurements. Based on these simulations, the authors provide a more convincing argument that the magnetic field rotation measured by MAVEN is the result of the upstream IMF rotation detected by Tianwen-1, rather than an encounter with a crustal field. Furthermore, the inclusion of the magnetic field trace for an unperturbed IMF (Supplementary Figure 10) also demonstrates that the transition in the clock angle in the MSE coordinates predicted by the empirical

model is a shift in IMF orientation, rather than being an artifact of the model itself.

That being said, there are still some concerns that remain. These should be considered minor suggestions, but definitely need to be addressed before I would recommend this study for publication.

(1) The details of the global hybrid simulation and the results of the simulations are lacking clarity. Lines 658-659 discuss some of the inputs into the simulation, but these values (solar wind velocity, density, temperature) require some justification. How long was the simulation run for (in simulation time?). Did the simulation reach stability? Was it first run in steady-state before time-accurate mode was introduced? These details should be included in the methodology section.
We thank the reviewer for the careful evaluation and for these constructive suggestions. We address each sub-point below.

Justification of solar wind input parameters: The upstream solar wind in the hybrid simulations is characterized by a number density of 3.3 cm^{-3}, a temperature of 1×10^5 K, and bulk velocity of 360 km/s directed along the -X direction (Lines 690-692). These values were derived from Tianwen-1, obtained in the undisturbed upstream solar wind during the analyzed event period. These values are consistent with the statistical properties of the solar wind upstream of Mars reported by Liu et al. (2021), based on MAVEN observations. They also correspond to the typical solar wind conditions adopted in previous simulation studies of the Martian plasma environment (Ma et al., 2019; Zhou et al., 2026). We have added these justifications with the corresponding references to the revised manuscript.

Simulation duration and steady-state verification: The total simulation duration is 450 s of physical time. Each simulation case was initialized with a uniform solar wind flow and a fixed upstream IMF throughout the entire domain, then run continuously with constant upstream boundary conditions. The initial flow-through phase allows the solar wind to fill the simulation domain and establish the bow shock, magnetic pileup boundary, and draped magnetic field configuration around Mars. We verified the attainment of a quasi-steady state by monitoring the time evolution of global quantities—including the total energy within the simulation domain, the positions of the bow shock and the magnetic pileup boundary, and the total ion escape flux. These quantities stabilize after approximately 300 s, with relative variations below 5% thereafter. The simulation results presented in this study are extracted at t = 400 s, well within the quasi-steady phase. No time-accurate mode was adopted in our simulations. Both simulation cases were solved exclusively in the steady-state framework and were continuously evolved until a quasi-steady state was reached. We have clarified these details in the revised manuscript (Lines 708-711).

Furthermore, a figure showing the global simulation output would be useful for readers to check the accuracy and plausibility of the draped magnetic field. This current manuscript only contains spacecraft traces through the simulation, but a global picture of the output for the two IMF cases with a sample MAVEN and Tianwen-1 trajectory should also be included, perhaps in the supplementary information. This could also help demonstrate visually that the crustal fields are not the dominant source of magnetic field within the simulation domain.

We thank the reviewer for this helpful suggestion. Following the recommendation, we have added a new figure (Figure B1/Supplementary Fig. 4, Line 169) presenting global views of the hybrid simulation output for both IMF cases. This figure displays the three-dimensional magnetic field lines and draped magnetic field patterns in representative cut planes (the equatorial plane, the noon – midnight meridian plane, and the dawn – dusk plane. The magnetic field magnitude distributions are in the X-Y and X-Z planes under the two upstream IMF conditions: [Bx, By, Bz] = [2.30, -2.38, 1.06] nT and [Bx, By, Bz] = [-1.94, -1.74, -2.88] nT. The projected trajectories of MAVEN and Tianwen-1 are overlaid in each panel. This global view demonstrates that: (i) the large-scale draped magnetic field configuration is consistent with expectations for the given upstream IMF orientations; (ii) the crustal magnetic fields produce only localized perturbations and do not dominate the magnetic field along the spacecraft trajectories; and (iii) the change in the global magnetic field topology between the two IMF cases is clearly visible.

We have included this figure in the Supplementary Information and added corresponding references in the revised manuscript.

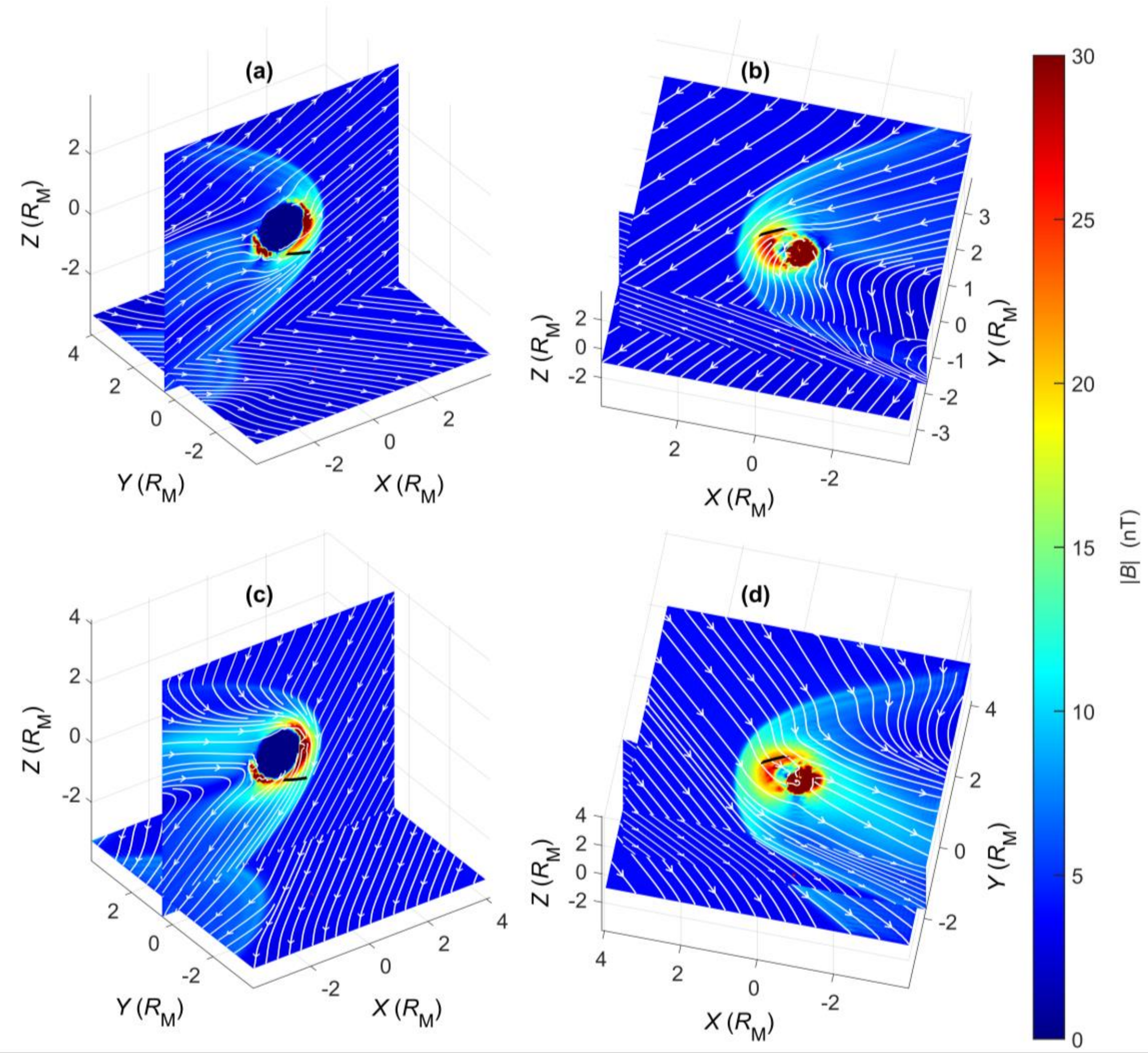

Figure B1. Distribution of magnetic field magnitude in the X-Y and X-Z planes under two upstream IMF conditions: (a – b) [Bx, By, Bz] = [2.30, -2.38, 1.06] nT and (c – d) [Bx, By, Bz] = [-1.94, -1.74, -2.88] nT in MSO coordinates. The black and red curves in each panel denote the trajectories of MAVEN and Tianwen-1, respectively. The Martian crustal magnetic field is included in the

simulation. This global view demonstrates that: (i) the large-scale draped magnetic field configuration is physically reasonable and consistent with the corresponding upstream IMF orientations; (ii) the crustal magnetic fields introduce only localized perturbations and do not dominate the magnetic field structure along the spacecraft trajectories, along the spacecraft trajectories, where the magnetic field is primarily characterized by the draped configuration; and (iii) the large-scale magnetic topology differs significantly between the two IMF cases.

(2) The pointing direction/field-of-view of STATIC should be accounted for in the plume example. Given that the inferred response timescale relies on the apparent onset of the plume, the authors should more rigorously demonstrate that this onset is not driven by changes in viewing geometry. For example, a comparison between the expected plume direction and the instrument field of view before and after the IMF rotation could demonstrate that the plume onset was the result of the change in convectional electric field, not a change in viewing geometry.
We thank the reviewer for this helpful comment. We agree that the STATIC viewing geometry should be considered when identifying the plume onset.

To address this issue, we added a comparison between the STATIC field of view and the expected plume direction. Since the O^+ plume is generally along the solar wind convection electric field direction, we projected the solar wind electric field direction into the STATIC instrument frame and compared it with the STATIC field of view before and after the IMF rotation. The new analysis is shown in Figures B2d & B2e, where the white curves indicate the projected solar wind electric field direction within the STATIC field of view.

The result shows that the solar wind convection electric field direction remained within the STATIC field of view throughout the interval of interest, both before and after the IMF rotation. Therefore, the observed plume onset is unlikely to be caused by a change in viewing geometry. Instead, this supports our interpretation that the plume onset was associated with the change in the convection electric field caused by the IMF rotation.

We have added this analysis to the revised manuscript and included the corresponding figure in the Supplementary Information.

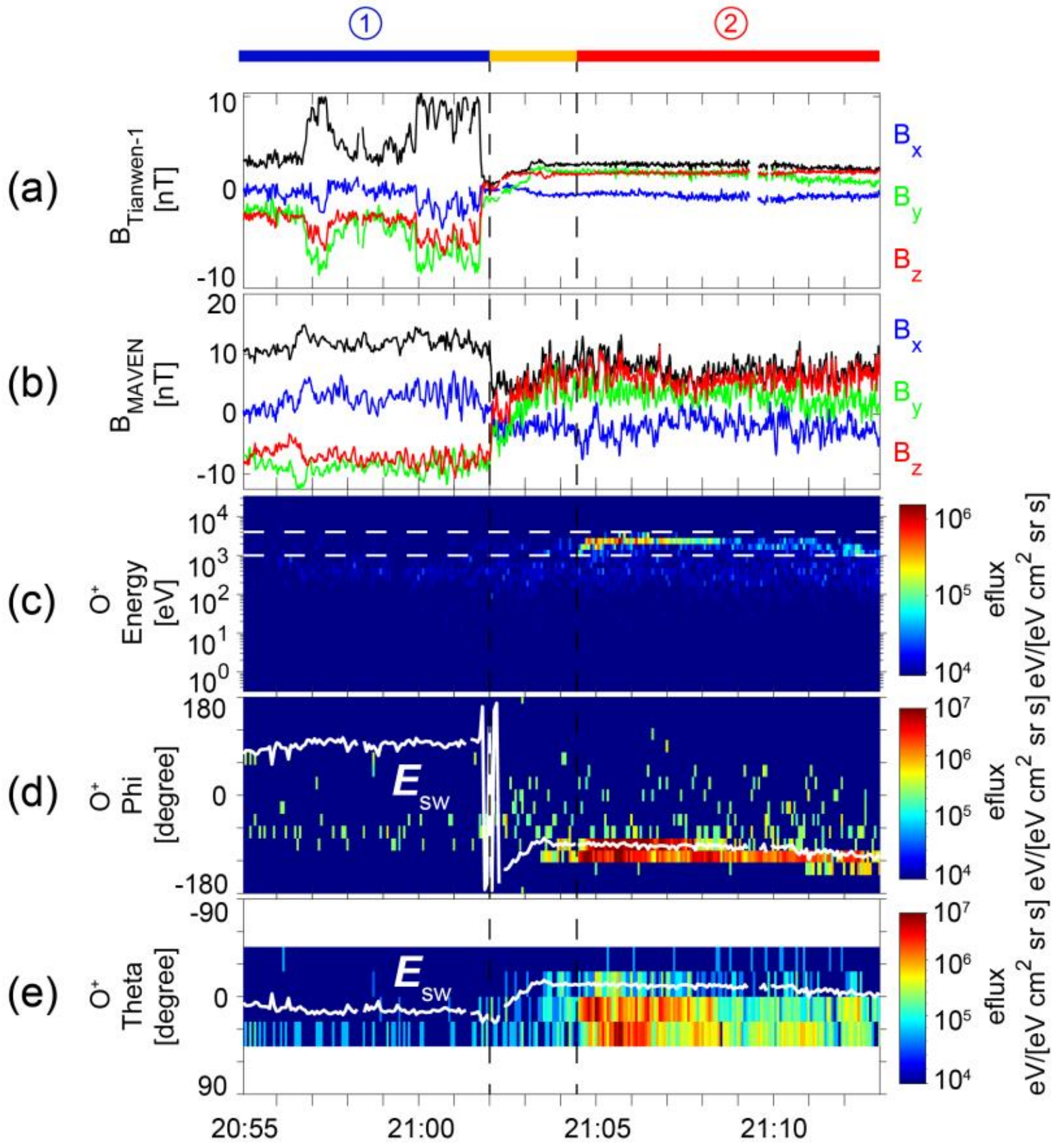

Figure B2. The field of view of STATIC/MAVEN and solar wind convective electric field in the time interval of 20:55-21:13 UT on November 16, 2021. The White curves in panel d and e represent the solar wind electric field direction in the STATIC instrument frame.

Line 177: Not sure what you mean by “recovery” here. Should be more specific like “recovery of the draped magnetic field to this rotation”

Corrected.

Lines 601-612: Some typos with “ref” instead of just placing the references.

Corrected.

Reply to the Reviewers

We greatly thank the reviewers for the valuable comments/suggestions that we considered in the revised manuscript. All revised parts are marked in blue in the text. Detailed answers to the comments point-by-point are given below:

--

Reply to Reviewer #4:

First of all, I would like to express my appreciation for all the hard work that the authors put in to address my comments. They have addressed most of them. However, my concern about the crustal field influence remains, despite the extended arguments from the authors. See my reasoning below. At this point, I think we are at an impasse, and I' m not sure what more the authors could do to convince me that there is no crustal field effect in Case 1.

Here is my interpretation of this case study. After the upstream IMF rotation at ~23:08, the magnetic field measurements in the magnetosphere/ionosphere have similar induced-like structures as before, up to ~23:10:30. This suggests that there is a delay in the response of the system to the upstream IMF rotation, which is an interesting and valid result. However, after 23:10:30, the spacecraft enters a region where crustal magnetic fields start to have an effect (does not have to be the dominant term), which makes it very difficult to put an end time for the recovery phase.

Therefore, I would recommend revising the manuscript as follows: (a) provide a lower limit of ~2.5 min for the recovery time for this case study and (b) explain that the unfortunate interference of crustal fields at low altitude makes it difficult to pinpoint the exact duration of the recovery phase. In this case, relevant arguments about having no crustal effects will need to be removed from the main text and SI.

My counterarguments to the responses from the authors:

1. Re: "A single-event agreement in Bz is not sufficient to demonstrate crustal-field dominance." To me, whether crustal fields dominate is actually a case-by-case phenomenon, even for the same spatial location (altitude, latitude, and longitude). Considering only pressure balance, it is roughly determined by the relative strength of the normal solar wind dynamic pressure and the magnetic pressure of the crustal fields, ignoring ionospheric thermal pressure, or possible magnetic reconnection with IMF, etc. First, naturally, whether crustal fields dominate depends on the specific upstream solar wind dynamic pressure (Pdyn). Second, the orbit segment of interest (23:08 – 23:16) takes place near the terminator, with SZA varying from 68° to 95°. At these SZAs, normal Pdyn is much smaller than in subsolar regions, and even weaker crustal fields can sometimes dominate at low altitudes. I assume Figure A2 is averaged over all possible SZA and upstream Pdyn conditions? This figure cannot disprove that in this particular case, crustal fields are not dominant. For this reason, I do not think you should include this argument in the SI.

2. Re: "The crustal field models do not reproduce the overall observed magnetic vector."
I should have clarified in my previous comments that I do not think that there is no induced magnetic

field at 23:08 – 23:16. Quite the opposite, I think there is a persistent induced magnetic field in the BY (and in the BX, at least before 23:10). This orbit segment is near the terminator and the south pole, where the draped magnetic fields should be mainly in the X-Y direction for a Parker IMF. In this draping geometry, Bz roughly corresponds to the radial component and should be the "least induced" component, compared to the other two components. This is why I used the measured BY and crustal field BZ to calculate the clock angle. Additionally, I noticed that the Bx component approaches BX, model from crustal field models at 23:14 UT, to be nearly 0, suggesting that it is not induced-like either. The structures in Bx between 23:12 and 23:14 could be a result of the interaction between the crustal fields and induced fields. In fact, the more I think about it, the more convinced I am that the magnetic field observations (Bz and, arguably, Bx) past ~23:11 are strongly influenced by the local crustal fields.

3. Re: "Simulations with and without crustal fields show that the crustal contribution is small."
I looked at the modeled results again from the previous version of the response (copy and paste above). The model is not reproducing the magnetic field components well after the IMF rotation (past 23:08). As you can see, the dominant component past 23:12 is -BX, while the data shows the dominant component is BY. Additionally, the modeled clock angle agrees better with data for the simulation without CF than the one with CF, comparing the blue and black lines in Figures 2c and 2d. It seems odd that the comparison is worse when more realistic inputs are used in the model. I'm not saying that the model results are incorrect, but at least it deserves some doubts.

We thank the reviewer for the in-depth physical analysis and constructive comments/suggestions on crustal magnetic field contamination, and we sincerely appreciate the reviewer's recognition of the scientific value of this work.

We fully agree with your key point that the crustal magnetic fields can affect the low-altitude portion in our event. Some statements in the previous version of the manuscript may not have expressed this point sufficiently clearly and may therefore have caused misunderstanding. In the revised manuscript and Supplementary Information, we have corrected the relevant wording and now explicitly acknowledge the presence of crustal magnetic field effects in Case 1.

After carefully reconsidering the reviewer's comments, we agree that the available evidence does not allow the crustal and induced contributions to be separated unambiguously after approximately 23:10:30 UT. In particular, the agreement between the observed B_z component and that predicted by the crustal magnetic field model prevents us from completely excluding a significant crustal-field contribution during the later low-altitude interval.

Accordingly, the revised manuscript no longer relies on the assumption of negligible crustal magnetic field effects to establish the primary conclusion of Case 1. Instead, the simultaneous Tianwen-1 and MAVEN observations are used to demonstrate a delayed, rapid magnetic response of Mars' induced magnetosphere to the upstream IMF rotation. The magnetic field measured by MAVEN retained induced-like structures similar to those before the IMF rotation until approximately 23:10:30 UT. Measured from the onset of the IMF rotation observed by Tianwen-1 at 23:07:45 UT, this interval provides an observational lower limit of 165 s for the magnetic recovery

time. Because local crustal-field effects cannot be excluded after approximately 23:10:30 UT, the exact completion time of the magnetic recovery cannot be determined unambiguously from this event.

After carefully considering the reviewer's suggestions, we have revised the manuscript as follows:

(a) In the Results section, Case 1 is now used to demonstrate the rapid and delayed response of the induced magnetospheric magnetic field, and we provide an observational lower limit of 165 s for the magnetic recovery time. We no longer assign a unique recovery endpoint to this event.

(b) The original estimate of the possible full recovery time has been moved from the Results section to the Discussion section. This estimate is now explicitly presented as an assumption-dependent reference value obtained only under the additional assumption that crustal magnetic field effects are negligible.

(c) We now explain that the crustal magnetic field model at low altitude prevents us from excluding a significant crustal-field contribution. Consequently, the exact duration of the recovery phase cannot be determined reliably in this event.

(d) In both the main text and the Supplementary Information, we have revised statements that could previously have been interpreted as claiming that crustal magnetic field effects were absent. The revised text now explicitly acknowledges their presence and clarifies the respective roles of the induced and crustal magnetic-field contributions.

We believe that these revisions preserve the robust observational result, i.e., the rapid response of Mars' induced magnetosphere, while acknowledging the uncertainty introduced by crustal magnetic fields at low altitude.

Reply to Reviewer #5:

I appreciate the authors' continued efforts to address these my concerns. My specific questions have now been largely answered, and the manuscript contains a substantial amount of additional analysis. However, in addressing my comments and those of the other reviewers, the manuscript has become difficult to follow. In its current form, the main scientific argument regarding the magnetospheric response to the IMF rotation has been obscured by such a large number of qualifications and supplementary analysis and figures.

The current presentation makes the main result more difficult to navigate. In several places, references in the main manuscript to supplementary figures do not provide enough context to explain how those figures support the overall argument being made in the main text. For example, it is not clear to me what Supplementary Figure 6 represents. The vague caption and brief reference in the main text make the figure difficult to interpret, and similar issues arise elsewhere in the Supplementary Information. The Supplementary Information now contains 16 figures, many of which deserve more explanation in the main text, or or require clearer justification for their inclusion as supplementary material.

My concern is now whether the manuscript in its current form presents a coherent and accessible case for its main conclusions. I think the work would benefit from a more substantial restructuring, in which the authors identify the more essential evidence needed to support the central claim, which should be included more in the main text rather than briefly described in the Supplementary Information.

Given the extent of restructuring required, I cannot recommend publication in its current form. I would support either a major revision focused specifically on clarity and prioritization of evidence, or consideration of resubmission to a journal with a format better suited to the length of the study.

We thank the reviewer for the constructive comments and suggestions, and we also appreciate the reviewer's recognition of this work.

We agree that the previous version of the manuscript had become overly complex and that the central conclusion and supporting line of evidence were not presented with sufficient clarity. In the revised manuscript, we have substantially restructured the presentation around the central conclusion that Mars' induced magnetosphere responds rapidly to changes in the interplanetary magnetic field.

In addition, we have substantially reduced the Supplementary Information by removing redundant figures and analyses and retaining only the material that is essential for supporting the main conclusions. The captions of the remaining supplementary figures have been expanded and clarified, and the corresponding figures are now cited and explained at appropriate locations in the main text. Detailed descriptions of the empirical model, the IMF-dependent MSE sampling procedure, and the limitations of the analysis have been moved to the Methods section so that the Results section remains focused and easier to follow.

The Discussion section has also been reorganized. It first addresses the assumption-dependent recovery-time estimate and the possible influence of crustal magnetic fields, and then discusses the broader implications of the rapid response for the variability of the Martian space environment and for the importance of multi-spacecraft observations in monitoring Mars' space weather.

In addition, to address the comments from Reviewer #4, we have separated the observational evidence for rapid response from the assumption-dependent estimate of the full recovery time. The Results section now reports only the robust timing constraints: the 165 s lower limit for the magnetic recovery time in Case 1 and the plume-response time in Case 2. The possible full recovery time derived from comparisons with the empirical model and hybrid simulations is discussed separately in the Discussion section and is explicitly identified as conditional on the assumption that crustal magnetic field effects are negligible.

We believe that the revised manuscript now presents a clearer hierarchy of evidence, a more coherent line of reasoning, and a more focused distinction between the robust observational conclusions and the assumption-dependent interpretations.

Reply to the Reviewers

We greatly thank the reviewers for the valuable comments/suggestions that we considered in the revised manuscript. All revised parts are marked in blue in the text. Detailed answers to the comments point-by-point are given below:

--

Reply to Reviewer #4:

The authors have addressed all of my comments. I have no further comments.

Thanks again for your previous valuable comments and suggestions!

--

Reply to Reviewer #5:

I would like to reiterate my appreciation for the authors' efforts to address my concerns regarding the structure of the manuscript. This latest version presents a much clearer and more focused argument, and the authors have clearly gone to considerable effort to improve the manuscript's readability. In particular, the removal of the numerous qualifications concerning the influence of crustal fields on the MAVEN measurements has substantially strengthened the study.

I now have only one minor comment regarding a supplementary figure, as described below.

Once this comment has been addressed, I would be happy to recommend the manuscript for publication.

Supplementary Figure 2: The caption states that the red curve represents the Tianwen-1 trajectory, but the trajectory is very difficult to identify in the figure. The figure could be improved by zooming in closer to Mars and more clearly indicating the spacecraft's location. I suggest including multiple 2D slices of the modeled magnetosphere, similar to Supplementary Figure 5, showing the XZ and XY planes with streamlines indicating the magnetic field direction. These views should be provided both before and after the IMF rotation. This would make the simulation results significantly clearer and easier for the reader to interpret.

We appreciate the reviewer's constructive suggestions and support. We have revised Supplementary Figure 2 to make the satellite trajectories in Supplementary Figure 2 clearer and more explicit. And following your suggestion, we have added 2D slices of the simulations (Supplementary Figure 3), in which satellite trajectories and streamlines are also labeled consistently.

Thanks again for your valuable comments and suggestions!

Review of Lin et al., 2024 “Direct Observations of Rapid Response of Martian Induced Magnetosphere to Interplanetary Magnetic Field Rotation”

Please note this review follows the directed format of https://www.nature.com/ncomms/for-reviewers/writing-your-report and does not make line by line suggestions regarding grammar.

1. **Key results**
   Your overview of the key messages of the study, in your own words, highlighting what you find significant or notable. Usually, this can be summarized in a short paragraph.

   This manuscript presents a novel study that investigates a comparison between MAVEN and Tianwen-1 data. This is a worthwhile pursuit; comparisons studies at Mars are rare even though multiple spacecraft obtaining plasma and magnetic field data are present. The manuscript aims to combine these two datasets to estimate various global responses (planetary ion plume, magnetic field draping) from MAVEN to an upstream IMF rotation as measured by Tianwen-1. A main argument of this paper is the relevance of these results to both Venus, and Titan studies. It is argued that these findings are to some extent, universal in induced magnetospheres. This reviewer understands the challenges inherent in cross-mission studies and finds that this undertaking provides a useful comparison for the field. As written however, the manuscript could be improved in: validity, approach, and clarity to enhance the accuracy, impact, and accessibility of these findings to the wider Mars and induced magnetospheres communities.

2. **Validity**
   Your evaluation of the validity and robustness of the data interpretation and conclusions. If you feel there are flaws that prohibit the manuscript's publication, please describe them in detail.

   In general, approaching estimations of IMF response with multiple spacecraft is appropriate. However, there are limitations in the approach that raise significant doubts as to the interpretation of the findings. For example:

   - **Crustal Field and MSE:** At Mars it is well known that the crustal fields can influence observed magnetic fields at high (e.g. ~1000 km) altitudes and in particular will alter observations over the southern hemisphere as compared to the northern hemisphere. In addition, much has been written about the global draping morphology as unexpected, or hemi-spherically dichotomous due to either polar differences or hemi-spherically dependent draping fields in the magnetosheath. The approach of a ‘mean background field’ is a good idea, however using MSE obfuscates hemispheric differences including crustal fields and draping differences; IMF asymmetries, and low altitudes over the southern hemisphere are always circumspect to the crustal fields.
     - It is also worth noting, that basing analyses’ in MSE makes assumptions about the upstream IMF. It is unstated if these are mitigated in the author’s approach.
     - Taken in aggregate, it is unclear if the results presented here are truly dependent on the upstream IMF, or a result of crustal fields or other MSE effects.

- **Assumptions of Draping:** Throughout the manuscript it is assumed that the IMF upstream maps *directly* to the draped orientation. It is known that this is a limited assumption, especially under radial (or significant IMF Bx) conditions and this manuscript could benefit from additional discussion of how this limitation is addressed.
  - For example, the manuscript focuses on IMF along X but uses the clock angle in Y and Z, there is an unstated assumption as to this interpretation.

- Relevant (non-exhaustive) references for the above items:
  - Chai et al., 2019 https://iopscience.iop.org/article/10.3847/2041-8213/aaff6e/meta and relevant citations within incl. Dubinin about polar field effects from (assumably) plume dynamics
  - Zhang et al., 2022 https://agupubs.onlinelibrary.wiley.com/doi/abs/10.1029/2022JE007334, a more complete MSE based understanding of global magnetic fields
  - Azari et al., 2023 https://agupubs.onlinelibrary.wiley.com/doi/full/10.1029/2023JA031546, quantification of crustal fields effects at high altitudes, demonstration of non-direct draping
  - Liemohn et al. 2017 https://agupubs.onlinelibrary.wiley.com/doi/full/10.1002/2016JA023707 discussion of the effective limitations of both MSO and MSE
  - Langlais et al., 2019 https://agupubs.onlinelibrary.wiley.com/doi/full/10.1029/2018JE005854, crustal field model (one of a few including several previously)
  - Brain+2006 https://www.sciencedirect.com/science/article/abs/pii/S0019103505004902?via%3Dihub, demonstration of potential IMF based draping asymmetries
  - Fowler+2022, https://agupubs.onlinelibrary.wiley.com/doi/abs/10.1029/2022JA030726 discussion of radial IMF

3. Significance

Your view on the potential significance of the conclusions for the field and related fields. If you think that other findings in the published literature compromise the manuscript's significance, please provide relevant references.

- This study provides (to the reviewer's understanding) the first multi-spacecraft estimation of response rate of the IMF at Mars. However previous studies https://agupubs.onlinelibrary.wiley.com/doi/full/10.1029/2019GL084151 have provided such estimations and these are only superficially compared to the results.
- In addition, there are severe limitations for comparison to other planets (e.g. Venus, Titan) given the significantly different atmospheric constituents (and ionospheric

compositions), densities, and upstream conditions (e.g. Titan is in Saturn's magnetosphere). It is unclear how the author's considered such limitations and casts doubt as to the claim regarding unified theories of induced magnetospheric response rates to upstream conditions.

4. **Data and methodology**
Your assessment of the validity of the approach, the quality of the data, and the quality of presentation. We ask reviewers to assess all data, including those provided as supplementary information. If any aspect of the data is outside the scope of your expertise, please note this in your report or in the comments to the editor. We may, on a case-by-case basis, ask reviewers to check code provided by the authors (see this Nature editorial for more information).Reviewers have the right to view the data and code that underlie the work if it would help in the evaluation, even if these have not been provided with the submission (see this Nature editorial). If essential data are not available, please contact the editor to obtain them before submitting the report.

The data used is appropriate. Please note that http://202.106.152.98:8081/marsdata/ is unreachable at the time of the reviewer's report.

5. **Analytical approach**
Your assessment of the strength of the analytical approach, including the validity and comprehensiveness of any statistical tests. If any aspect of the analytical approach is outside the scope of your expertise, please note this in your report or in the comments to the editor.

There are several minor issues regarding the approach that could be addressed with additional text.
    - It is concerning that the correlation coefficient peaks in the positive time scales. More details would be useful in deciphering this.
    - There are a few different types of correlation coefficients, it would be useful to add context to this discussion to understand the assumptions being made.
    - More details on the empirical model would assist in interpretation, for example this is a gridded product in the associated supplementary, but is used for continuous estimation in the plots.

6. **Suggested improvements**
Your suggestions for additional experiments or data that could help strengthen the work and make it suitable for publication in the journal. Suggestions should be limited to the present scope of the manuscript; that is, they should only include what can be reasonably addressed in a revision and exclude what would significantly change the scope of the work. The editor will assess all the suggestions received and provide additional guidance to the authors.

- Most of the comments above (and below, see clarity and context) can be addressed with additional details. There are several issues however that will require additional pursuit

including potentially: assessing the impact of crustal field in these analyses and/or using non MSE coordinate systems for these analyses.

- Additional grammar edits (see clarity and context).

7. Clarity and context

Your view on the clarity and accessibility of the text, and whether the results have been provided with sufficient context and consideration of previous work. Note that we are not asking for you to comment on language issues such as spelling or grammatical mistakes.

- The motivation behind this work is unclear with respect to the connection between Titan, Venus, and Mars. For example, it is expected that these should be significant differences within the theory of induced magnetospheres but such differences are not discussed.

8. References

Your view on whether the manuscript references previous literature appropriately.

A discussion of induced magnetospheres is warranted in this work but not provided. Several substantial papers regarding fundamental review of induced magnetospheres in space physics are not included such as:

- Luhmann+2004, https://www.sciencedirect.com/science/article/abs/pii/S0273117704000158

9. Your expertise

Please indicate any particular part of the manuscript, data or analyses that you feel is outside the scope of your expertise, or that you were unable to assess fully.

N/A

Round 2 Review of Lin et al., 2024 "Direct Observations of Rapid Response of Martian Induced Magnetosphere to Interplanetary Magnetic Field Rotation"

Overview:

I thank the authors for their response to my concerns. The following are remaining items that I still recommend addressing for review.

Major

- The manuscript still has several syntax issues throughout the current manuscript. I urge the authors to consider pursuing an external review solely for syntax. The current manuscript is difficult to review fully in its current iteration. This was similarly suggested by other reviewers.
    - For example:
        - Direct Observations of Rapid Response of Martian Induced Magnetosphere to Interplanetary Magnetic Field Rotation
            - Martian should be replaced with Mars', an 'a' should be inserted before Rapid Response, an "an" should be inserted before Interplanetary
        - Induced magnetosphere in non-magnetized planets or moons, formed by the interaction between its atmosphere/ionosphere and stellar wind or planetary plasma flows, is generally affected by external magnetic field.
            - This sentence is very unclear and should be rewritten for content and syntax.
            - It could be rewritten as "Induced magnetospheres of non-magnetized planets or moons are formed by the interaction between a planet's atmosphere and ionosphere and solar wind."

- Line 131, double check these times and claims?
    - The solar wind is still disturbed (IMF Bx spike) at 23:09 and MAVEN seems to respond to this at 23:12. This is worth additional text as it's one of the most obvious 'by-eye' correlations.

Minor

- Line 22 is unclear; I am unsure of what this sentence is conveying.
- Line 47, on the surface? What is this referring to?
- Line 609, citation?

Reviewer #4
(Remarks to the Author)

Review for "Direct Observations of the Rapid Response of the Mars' Induced Magnetosphere to an Interplanetary Magnetic Field Rotation" by Lin et al.

(1) The results of the hybrid simulations are not quite convincing. The clock angle in MSO from the simulations with crustal fields only has a very smooth long-time-scale variation. I'm not convinced that these simulations can reproduce the fine structures of crustal fields at low altitudes. It's likely that: either the grid resolution of the hybrid model is too coarse to capture the small-spatial-scale variations or the crustal field model used is not accurate enough.

(2) To examine the crustal field effect, I can simply use the crustal field model Bz component to calculate the clock angle. The 3rd panel in the figure below shows the calculated clock angle using the measured By & measured Bz (Bclock, blue) and measured By & Bz from the crustal model (Bclock_crustal, orange). Bclock_crustal matches well with Bclock from ~23:10 onward, suggesting that the clock angle past 23:10 is dominated by crustal field Bz. I still think the effect of crustal fields makes it extremely difficult to determine the recovery time for this case study.

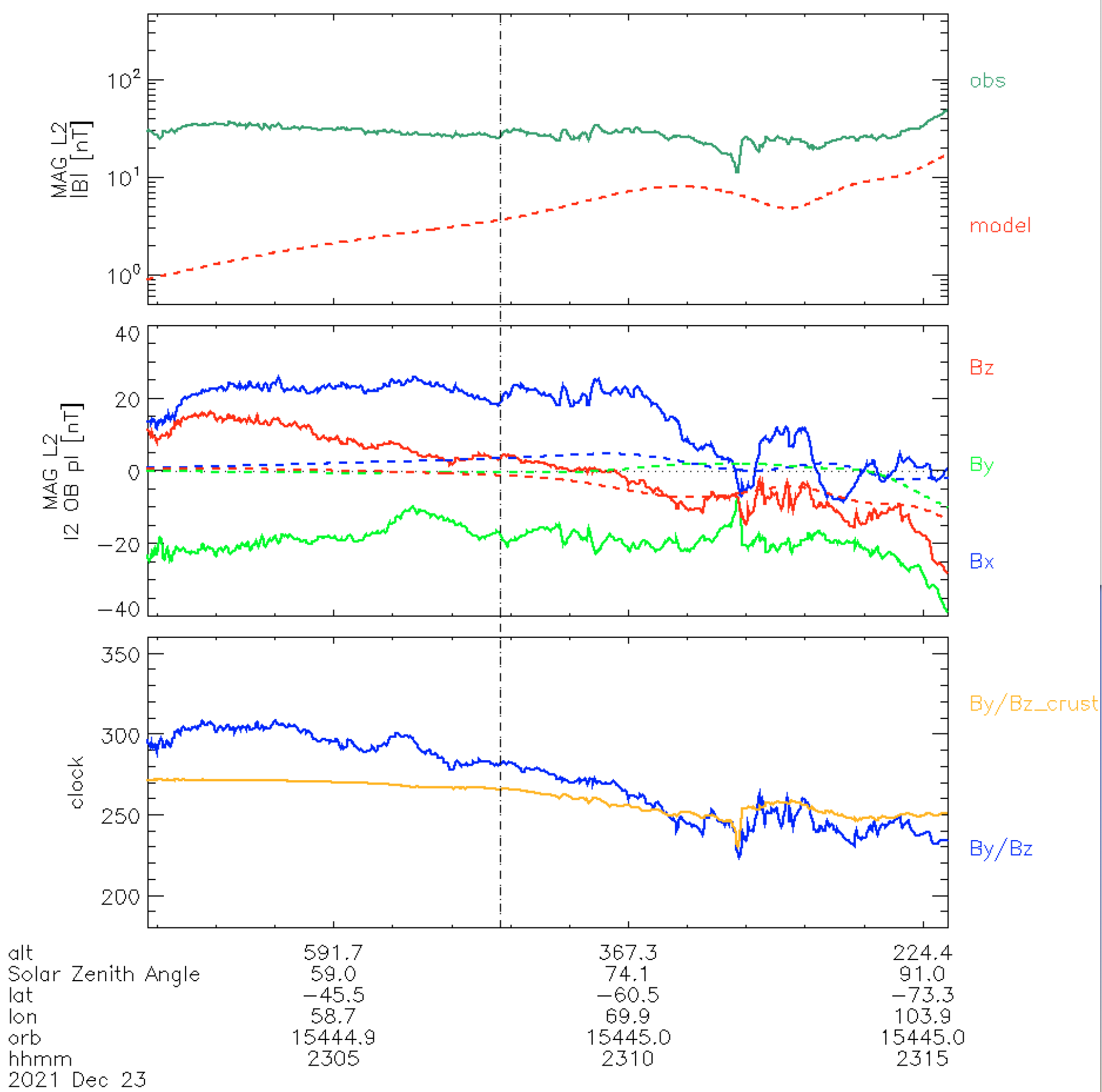

(3) I'm still very confused by the clock angle change in MSE using the empirical model. From what I understand, the empirical model is a 3-D steady-state magnetic vector field in the MSE coordinates. When you apply an IMF clock angle change, isn't it just rotate the whole vector field by the same degree for every given point in the empirical model? I do not expect it to be like the clock angle change with MAVEN data but it should be similar to the IMF clock angle change? Also, Figure A4 is very puzzling. At 23:08, the Bx and By components from the empirical model, similar to Bz,maven, but By,model stays about the same. Isn't IMF clock angle change mainly causing changes to By & Bz components but not Bx?

(4) Regarding the plume response time, the revised text is still a little confusing. If I understand it correctly, the calculation in Lines 357-364 is for the time scale of ion transport from the ionosphere to the IMB before ions "see" the main convectional electric fields. If so, I would suggest putting Lines 371-376 before Line 353 and simplifying the text a little.

Review for "Direct Observations of the Rapid Response of the Mars' Induced Magnetosphere to an Interplanetary Magnetic Field Rotation" by Lin et al.

First of all, I would like to express my appreciation for all the hard work that the authors put in to address my comments. They have addressed most of them. However, my concern about the crustal field influence remains, despite the extended arguments from the authors. See my reasoning below. At this point, I think we are at an impasse, and I'm not sure what more the authors could do to convince me that there is no crustal field effect in Case 1.

Here is my interpretation of this case study. After the upstream IMF rotation at ~23:08, the magnetic field measurements in the magnetosphere/ionosphere have similar induced-like structures as before, up to ~23:10:30. This suggests that there is a delay in the response of the system to the upstream IMF rotation, which is an interesting and valid result. However, after 23:10:30, the spacecraft enters a region where crustal magnetic fields start to have an effect (does not have to be the dominant term), which makes it very difficult to put an end time for the recovery phase.

Therefore, I would recommend revising the manuscript as follows: (a) provide a lower limit of ~2.5 min for the recovery time for this case study and (b) explain that the unfortunate interference of crustal fields at low altitude makes it difficult to pinpoint the exact duration of the recovery phase. In this case, relevant arguments about having no crustal effects will need to be removed from the main text and SI.

My counterarguments to the responses from the authors:

1. Re: "A single-event agreement in Bz is not sufficient to demonstrate crustal-field dominance."

To me, whether crustal fields dominate is actually a case-by-case phenomenon, even for the same spatial location (altitude, latitude, and longitude). Considering only pressure balance, it is roughly determined by the relative strength of the **normal** solar wind dynamic pressure and the magnetic pressure of the crustal fields, ignoring ionospheric thermal pressure, or possible magnetic reconnection with IMF, etc. First, naturally, whether crustal fields dominate depends on the specific upstream solar wind dynamic pressure (Pdyn). Second, the orbit segment of interest (23:08 – 23:16) takes place near the terminator, with SZA varying from 68° to 95°. At these SZAs, **normal** Pdyn is much smaller than in subsolar regions, and even weaker crustal fields can sometimes dominate at low altitudes. I assume Figure A2 is averaged over all possible SZA and upstream Pdyn conditions? This figure cannot disprove that in this particular case, crustal fields are not dominant. For this reason, I do not think you should include this argument in the SI.

2. Re: "The crustal field models do not reproduce the overall observed magnetic vector."

I should have clarified in my previous comments that I do not think that there is no induced magnetic field at 23:08 – 23:16. Quite the opposite, I think there is a persistent induced magnetic field in the B_Y (and in the B_X, at least before 23:10). This orbit segment is near the terminator and the south pole, where the draped magnetic fields should be mainly in the X-Y direction for a Parker IMF. In this draping geometry, Bz roughly corresponds to the radial component and should be the "least induced" component, compared to the other two components. This is why I used the measured B_Y and crustal field B_Z to calculate the clock angle. Additionally, I noticed that the Bx component approaches $B_{X,\, model}$ from crustal field models at 23:14 UT, to be nearly 0, suggesting that it is not induced-like either. The structures in Bx between 23:12 and 23:14 could be a result of the interaction between the crustal fields and induced fields. **In fact, the more I think about it, the more convinced I am that the magnetic field observations (Bz and, arguably, Bx) past ~23:11 are strongly influenced by the local crustal fields.**

3. Re: "Simulations with and without crustal fields show that the crustal contribution is small."

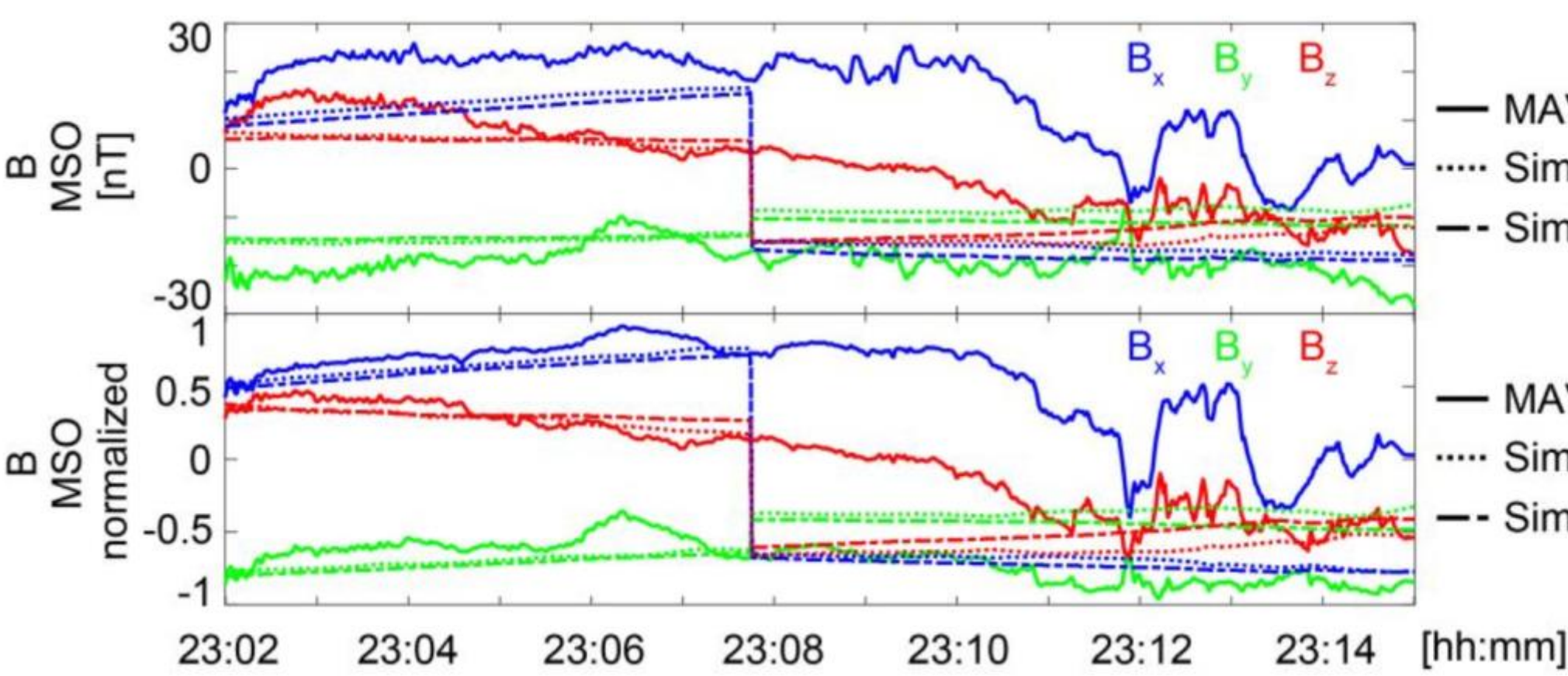

I looked at the modeled results again from the previous version of the response (copy and paste above). The model is not reproducing the magnetic field components well after the IMF rotation (past 23:08). As you can see, the dominant component past 23:12 is $-B_X$, while the data shows the dominant component is B_Y. Additionally, the modeled clock angle agrees better with data for the simulation without CF than the one with CF, comparing the blue and black lines in Figures 2c and 2d. It seems odd that the comparison is worse when more realistic inputs are used in the model. I'm not saying that the model results are incorrect, but at least it deserves some doubts.
